# An expeditious and efficient bromomethylation of thiols: enabling bromomethyl sulfides as useful building blocks†

**DOI:** 10.1039/c8ra04002h

**Published:** 2018-07-10

**Authors:** Carolina Silva-Cuevas, Ehecatl Paleo, David F. León-Rayo, J. Armando Lujan-Montelongo

**Affiliations:** Departamento de Química, Centro de Investigación y de Estudios Avanzados (Cinvestav) Av. Instituto Politécnico Nacional 2508, San Pedro Zacatenco 07360 Ciudad de México México jalujanm@cinvestav.mx; Facultad de Ciencias, Universidad Nacional Autónoma de México, Ciudad Universitaria 04510 Ciudad de México México

## Abstract

A facile and highly efficient method for the bromomethylation of thiols, using paraformaldehyde and HBr/AcOH, has been developed, which advantageously minimizes the generation of highly toxic byproducts. The preparation of 22 structurally diverse α-bromomethyl sulfides illustrates the chemo-tolerant applicability while bromo-lithium exchange and functionalization sequences, free radical reductions, and additions of the title compounds demonstrate their synthetic utility.

## Abstract 2

A new method for the bromomethylation of thiols using paraformaldehyde and HBr/AcOH, minimizes the generation of toxic byproducts. Synthetic utility of α-bromomethyl sulfides was demonstrated through umpolung and free radical chemistry.

## Graphical abstract

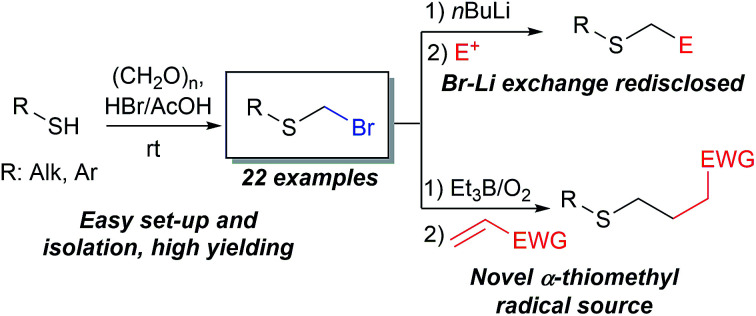

Heteroatom halomethylations^1^ have proven to be extremely useful for the generation of valuable synthetic intermediates.^2^ Halomethylation of thiols provides synthetically valuable chloromethylated intermediates (chloromethyl sulfides), which are typically prepared by condensation with bromochloromethane in basic media,^3^ or with HCl and a formaldehyde source (paraformaldehyde, polyoxymethylene, *etc.*).^4^ While chloromethyl sulfides have been traditionally used as alkylating reagents, the analogous bromomethyl counterparts offer superior electrophilicity, recognized since the earliest report describing their syntheses using hydrogen bromide and paraformaldehyde,^5^ yet they are often overlooked in this role. Moreover, the reactivity scope of bromomethyl thiol derivatives remains largely unexplored, despite a potentially broader synthetic range (*e.g.* for the generation of organometallics by metal–halogen exchange).^6^

Other methods for the generation of bromomethylated thiol derivatives consist of replacing hydrogen bromide gas with concentrated aqueous hydrobromic acid, along with a formaldehyde source (usually paraformaldehyde),^7^ or by using dibromomethane^8^ in basic media.^9^ Two or three-step procedures consisting of hydroxymethylation followed by substitution have also been developed.^10^ A desilylative rearrangement of α-TMS sulfides has also been used for the generation of bromomethylsulfides.^11^

As part of our interest in the preparation and application of structurally diverse sulfur-based building blocks,^12^ we investigated the preparation of benzyl(bromomethyl)sulfane (2a), previously used as an olefination reagent.^13^ However, several attempts to prepare 2a through exposure of benzylmercaptan (1a) to paraformaldehyde and hydrobromic acid,^7*b*^ led to a *ca.* 1.5 : 1 mixture of 2a (32%) and bis(benzylthio)methane 3a (21%, Scheme 1a). Iterations of the experiment always delivered important and variable amounts of the dithioacetal by-product 3a. On the other hand, an alternate approach to the bromomethylation of a cyclohexanethiol bromomethyl derivative 2b, using dibromomethane and K_2_CO_3_, resulted in trace amounts of the dithioacetal derivative 3b only (Scheme 1b).^14^

**Scheme 1:**
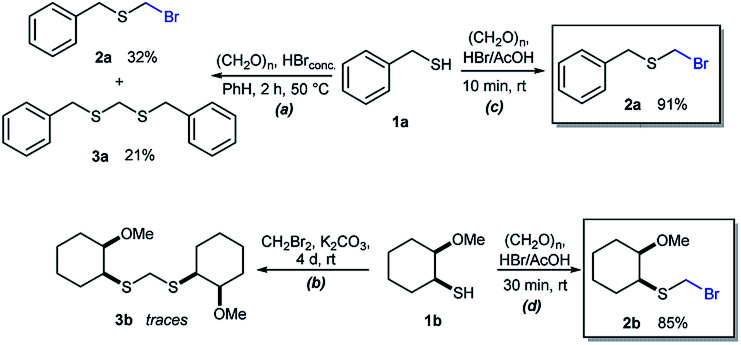
Attempts for the bromomethylation of 1a or 1b under (a) acidic or (b) basic media. (c) and (d) A highly efficient and direct approach for thiol bromomethylation (this work).

HBr/AcOH is a convenient hydrogen bromide source that minimizes exposure to risky set-ups and has been employed as a surrogate to highly corrosive and toxic hydrogen bromide gas in numerous applications.^15^ Although this reagent has been used previously in the generation of bromomethyl sulfides, installation of the methylene bridge required first a S-pivaloxymethylation of a mercaptan, followed by cleavage by HBr/AcOH.^16^

Surprisingly, sequential exposure of thiols 1a or 1b to paraformaldehyde and HBr/AcOH,^17^ rapidly delivered bromomethylated derivatives 2a and 2b with outstanding yields (Scheme 1). The simple experimental setup and straightforward purification procedure offer methodological utility; in most cases extraction with a low-boiling point hydrocarbon such as pentane or hexanes is sufficient to recover the material in high purity (>95%).^18^ Traces of impurities can be easily discarded through bulb-to-bulb vacuum distillation.

The reaction scope was explored with a series of structurally diverse thiols (Table 1). Aliphatic thiols yielded bromomethyl sulfides in excellent yields, although lower yielding 2g is attributed to its high volatility. *t*-Butyl bromomethyl sulfide (2i), a sterically challenging and volatile material that has been used as a synthetic equivalent of the methylmercaptan group (–CH_2_–SH),^19^ was prepared satisfactorily in 76% yield. Fluorinated bromomethyl 2e has been used for the preparation of fluorinated surfactants,^10*a*,20^ which some exhibit antimicrobial activity. As a previous method involves a 2-step sequence involving thiol hydroxymethylation and substitution by PBr_3_, our method directly delivered 2e in 88% yield.

**Table 1:** Thiol bromomethylation with HBr/paraformaldehyde^a^

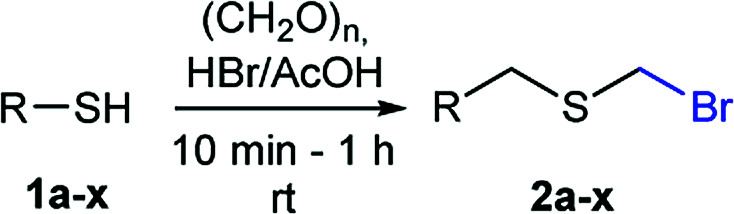			
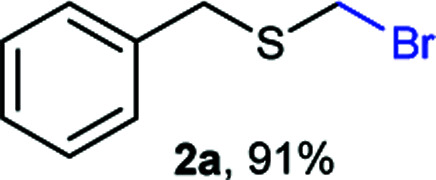	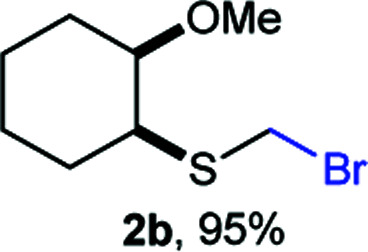	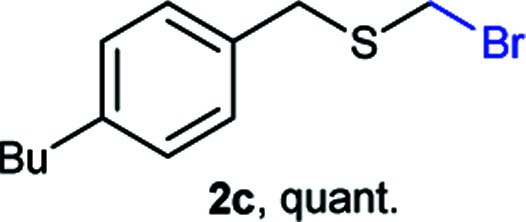	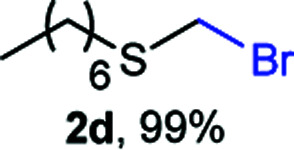
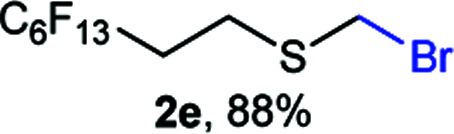	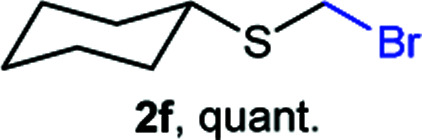	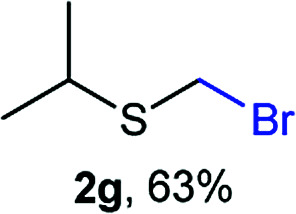	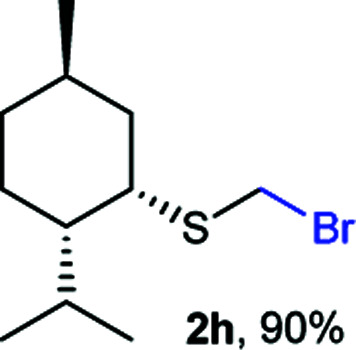
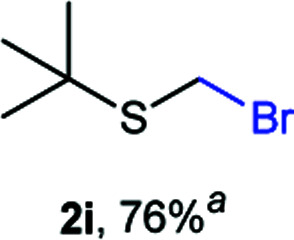	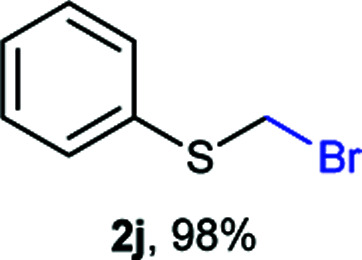	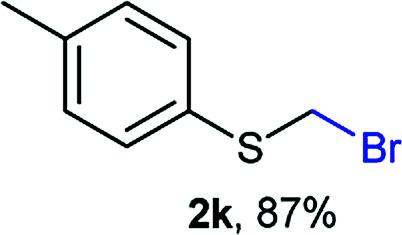	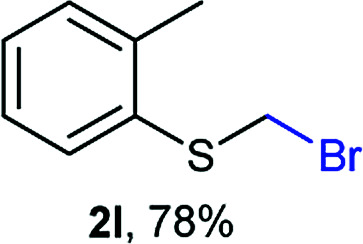
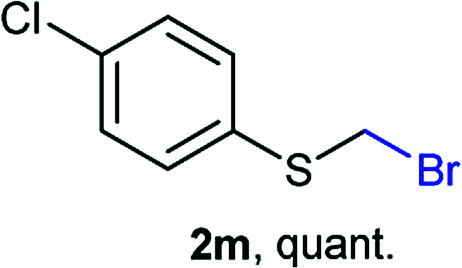	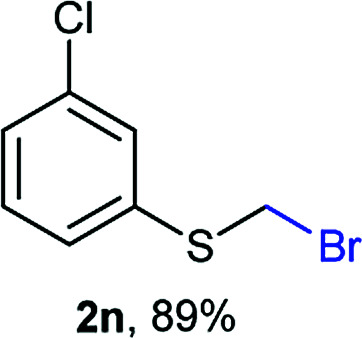	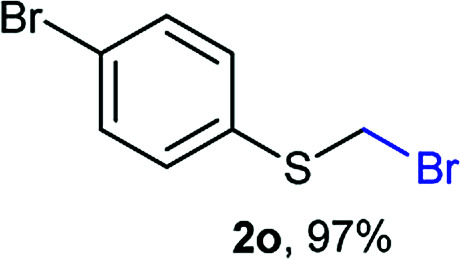	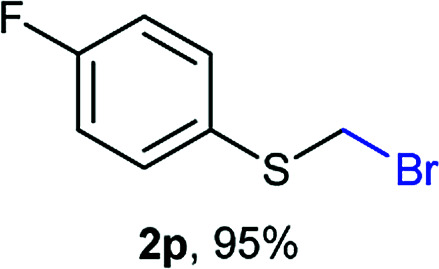
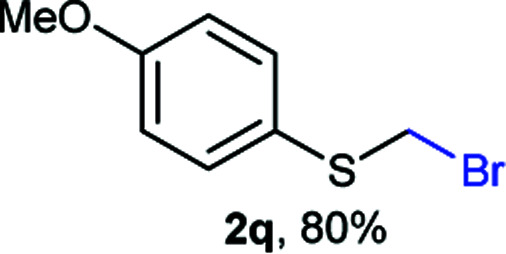	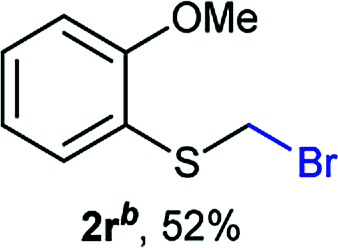	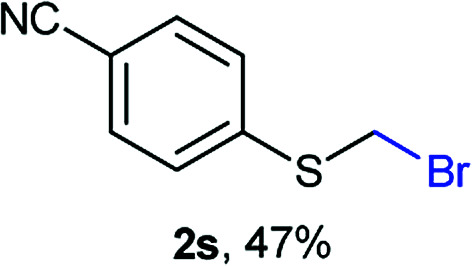	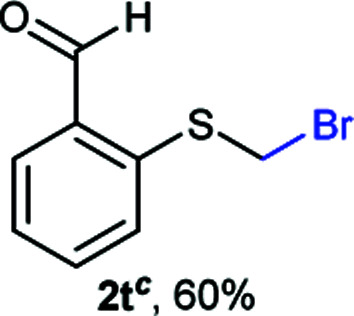
	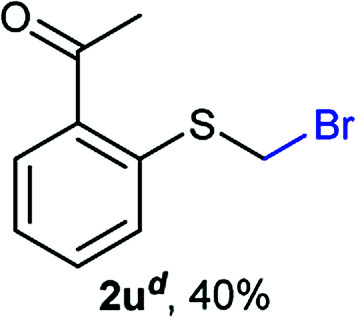	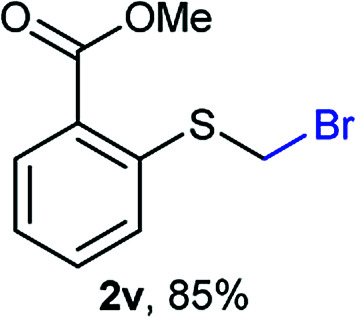	
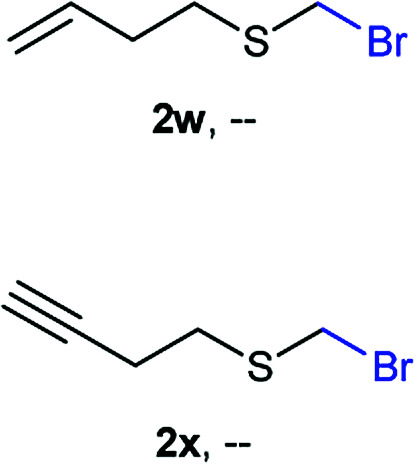	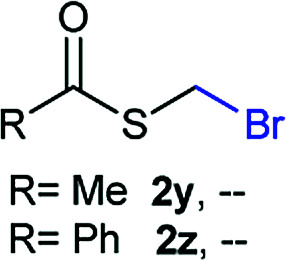	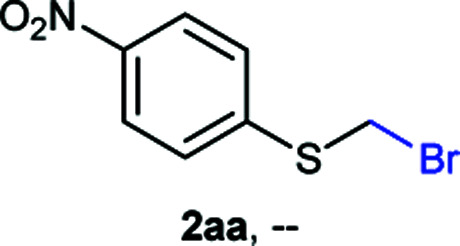	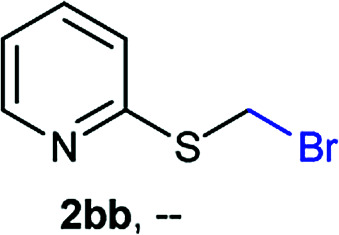

a Reaction was performed at −20 °C.

b Reaction was performed at 0 °C.

c Reaction was performed at 30 °C.

d Reaction was performed at 40 °C.

Aromatic substrates (2j–2v) were generally high yielding. For example, (bromomethyl)(phenyl)sulfane (2j), a useful electrophile and precursor to phenylthiomethyl azide^21^ and diethyl phenylthiomethane phosphonate, an olefination reagent,^22^ can be prepared in nearly quantitative yield. Aryl derivatives (bromomethyl)(4-methylphenyl)sulfane (2k) and (bromomethyl)(4-chlorophenyl)sulfane (2m), used in the preparation of [(*p*-phenylphenyl)oxy]methyl (POM) protective group,^23^ gave 87% and quantitative yields respectively. Comparatively, previously reported methods delivered 2k and 2m in 43% and 75% yield respectively.^11^ Anisyl thiol 1r was a challenging substrate, as the bromomethylation was highly exothermic and resulted in a near 1 : 1 mixture of bromomethylsulfide 2r and dithioacetal 3r. The yield of 2r was improved to 4 : 1 ratio, by cooling the reaction mixture to 0 °C. However, purification of 2r was also problematic as distillation led to partial decomposition. We speculate that integrity of 2r during preparation and purification is influenced by the neighbouring methoxy function. On the other hand, 2s–u modest yields are attributed to a decrease in S-nucleophilicity caused by the EWG groups. Interestingly, although thiol 1v bears an EWG at *ortho* position, methyl 2-((bromomethyl)thio)benzoate 2v was obtained in excellent yield (85%).

NMR analyses of a fresh mixture of paraformaldehyde and HBr/AcOH^24^ revealed a mixture consisting mainly of a component with an ^1^H-NMR 5.8 ppm signal, correlating to a ^13^C-NMR 68.2 ppm signal (HSQC). This species evolves mainly into two different components: one of them being bis(bromomethyl ether) as determined by a signal at 5.7 ppm (^1^H-NMR),^25^ and bis(bromomethoxy) methane (signals at 5.6 ppm and 5.0 ppm).^26^ The 5.8 ppm signal is presumed to belong to bromomethanol,^27^ which is consumed promptly by the thiol reagent. This is congruent with our observations, since the best results were obtained when the addition sequence consisted in adding the HBr/AcOH mixture to premixed thiol and paraformaldehyde (Scheme 2). Equimolar ratios of paraformaldehyde are enough for complete transformation, avoiding formation of potentially highly-toxic bis(bromomethyl ether).^28^ Alkenyl and alkynyl substrates (2w, 2x) were incompatible to this method as the bromomethylation procedure led to complex mixtures. Mercaptans featuring attenuated nucleophilicity such as thioacetic or thiobenzoic acids (2y, 2z), *p*-nitrothiophenol (2aa), and 2-mercaptopyridine (2bb) were unsuitable for this methodology.

**Scheme 2:**
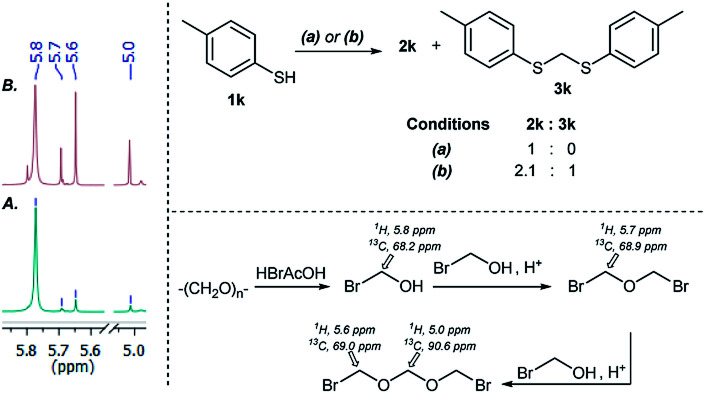
(A) ^1^H-NMR spectra of paraformaldehyde + HBr/AcOH (<1 min). (B) ^1^H-NMR spectra of paraformaldehyde + HBr/AcOH (after 5 min) (left). Conditions: (a) paraformaldehyde addition to 1k, 5 min, then HBr/AcOH addition, 45 min. (b) HBr/AcOH addition to paraformaldehyde, 5 min, then 1k, 45 min (top). Bromomethanol autocondensation decomposition pathway (bottom).

Attempts to diversify the α-alkyl component, found that exclusively highly reactive aldehydes underwent bromoalkylation with thiols (Table 2). Bromoalkylation yields using aldehydes is evidence that reaction efficiency is strongly dependent on the carbonyl reactivity, as bromo(4-nitrobenzylation) or bromoethylation of 4-methylbenzenethiol (1k) using electrophilic 4-nitrobenzaldehyde or acetaldehyde^29^ respectively, feature fair yields compared to the corresponding bromomethylation using paraformaldehyde (*cf.* entries 1, 3 and 4). Interestingly, thiol nucleophilicities have a larger impact in thiol bromoalkylations using aldehydes compared to bromomethylations with paraformaldehyde, as illustrated with superior reaction efficiency when benzyl mercaptan 1a was used instead of 1k (*cf.* entries 6, 7 and 9). Thiol bromoalkylation using ketones had no practical use as dithioketal 3k4 was the only product when acetone was used as the carbonyl component (entries 10 and 11) and acetophenone yielded a complex mixture (entry 12).

**Table 2:** Thiol bromoalkylation with selected carbonyl compounds

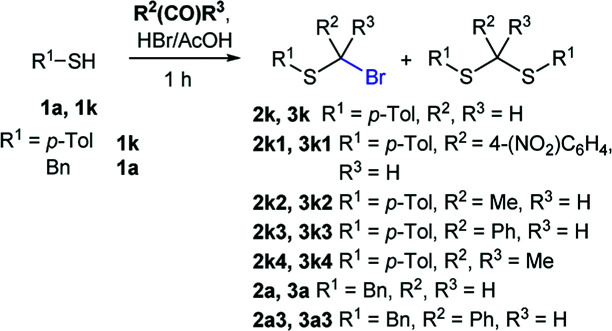						
Entry	*T*	R^1^-SH	R^2^	R^3^	2 yield (%)	3 yield (%)
1	rt	1k	H	H	2k (87%)	3k —
2	rt	1a	H	H	2a (91%)	3a —
3	rt	1k	4-(NO_2_)C_6_H_4_	H	2k1 (60%)	3k1 (21%)
4^a^	rt	1k	4-(NO_2_)C_6_H_4_	H	2k1 (67%)	3k1 (9%)
5	rt	1k	Me	H	2k2 (56%)	3k2 (36%)
6	rt	1k	Ph	H	2k3 (0%)	3k3 (69%)
7	30 °C	1k	Ph	H	2k3 (26%)	2k3 (69%)
8^a^	30 °C	1k	Ph	H	2k3 (46%)	2k3 (29%)
9	30 °C	1a	Ph	H	2a3 (61%)	3a3 (19%)
10	rt	1k	Me	Me	—	3k4 (52%)
11	40 °C	1k	Me	Me	—	3k4 (29%)
12	40 °C	1k	Ph	Me	Complex mixture	

a Reaction time 16 h.

To illustrate the versatility of bromomethyl sulfides as building blocks, we first carried out a polarity reversal through a halogen–metal exchange approach, a relatively rare procedure for the generation of α-sulfanylmethyl organometallics.^30–32^ This approach is underdeveloped, probably because of difficulties in synthesizing bromomethylsulfides.^33^ Classically, generation of α-sulfanylmethyl organolithiums has been carried out mainly by deprotonation.^34^ However, the deprotonation approach has important drawbacks, such as a substitution side-process that generate thiolates or regioselectivity issues when dialkyl sulfides are deprotonated.^35^ Sequentially exposing (bromomethyl)(cyclohexyl)sulfide (2f) or (bromomethyl)(*p*-tolyl)sulfide (2k) to *n*BuLi, generated nucleophilic organolithiums 4f and 4k, that were quenched by benzaldehyde thus assembling alkylated derivatives 5f and 5k in good yields (Scheme 3a). Using (+)-neomenthanethiol bromomethyl sulfide derivative (2h) for the bromo-lithium exchange and benzaldehyde in the electrophilic quench, generated β-hydroxysulfide 5h in good yield albeit low diastereoselectivity (*ca.* 1.4 : 1). This constitutes a novel approach for the application of sulfenyl methyllithium organometallics for the access of β-hydroxysulfides, valuable intermediates or fragments of natural products and biologically relevant compounds, usually prepared under acidic media or free radical oxidative conditions.^36^ On the other hand, preparation methods of mixed or unsymmetrical dithioacetals are scarce,^37^ some of them displaying selectivity limitations.^38^ Similar bromo-lithium exchange/functionalization procedures were also carried out on probes 2f and 2k using diphenyldisulfide as electrophile,^39^ delivering mixed thioacetals 6f and 6k respectively also with good yields (Scheme 3b). The exceptional electrophilicity of bromomethyl sulfides 2f and 2k, also enabled the access to mixed thioacetals 6f and 6k by simple exposure to sodium thiophenolate,^40,41^ thus demonstrating the versatility of bromomethyl sulfides either as electrophiles or nucleophiles after umpolung.

**Scheme 3:**
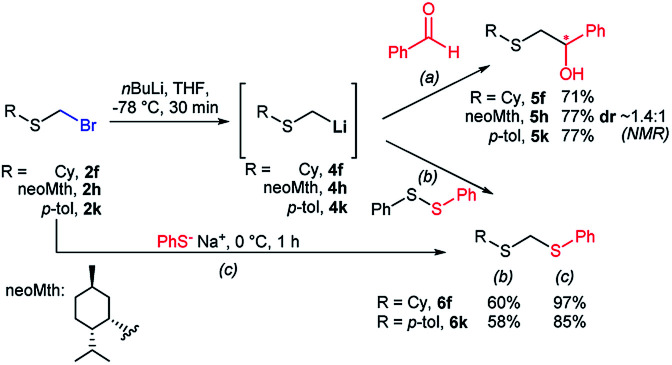
Br–Li exchanges on bromomethyl sulfides for the generation of nucleophilic sulfanylmethyllithiums: (a) β-hydroxysulfide syntheses, (b) unsymmetrical dithioacetal synthesis. (c) Alternate unsymmetrical dithioacetal synthesis by exploiting bromomethyl sulfides (2) electrophilicity.

To our knowledge, bromomethyl sulfides 2 have not been exploited for C–C bond construction through free radical chemistry. As far as we know, there is a single reference to an unrealized effort attempting an intramolecular free radical cyclization of an unavailable alkenyl bromomethylsulfide.^14,42^ Although our method unfortunately was not compatible with the direct preparation of alkenylsulfide bromomethyl derivatives (see Table 1), we could demonstrate exceptional reactivity of α-thiomethyl radical 7k (generated from 2k), towards (TMS)_3_SiH reduction,^43^ thus generating thioether 8k (Scheme 4a). On the other hand, Et_3_B initiated^44^ additions of nucleophilic radicals^40,45^7f and 7k on radical acceptors acrylonitrile and methyl acrylate led to the generation of γ-functionalized sulfides 9f, 9k, 10f and 10k (Scheme 4b). This constitutes a novel approach for the synthesis of γ-sulfanyl butanenitriles and esters, as an alternative to the thiol-ene reaction approach,^46^ and establishes bromomethyl sulfides as a new entry on the family of monothiomethyl radical sources.^47^

**Scheme 4:**
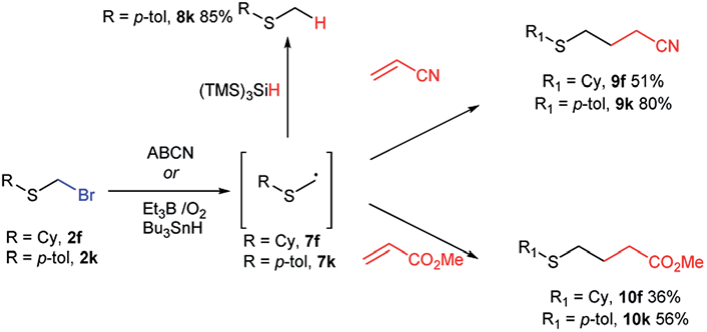
Unprecedented generation of α-thiomethyl free radicals from bromomethyl sulfides and their reduction and addition to acrylonitrile and methyl acrylate.

## Conclusions

Development of synthetic methods based on bromomethyl sulfides has been limited mainly by their ambiguous availability, as a consequence of methods lacking selectivity, efficiency, and perilous set-ups. We have developed a simple and high yielding method for thiol bromomethylation, that involves operational simplicity, and minimizes operational risk. The method has broad scope and good functional group tolerance but is unsuitable for low-nucleophilicity mercaptans. The bromomethylating reagent is suspected to be bromomethanol, obtained stoichiometrically and efficiently captured by thiols, thus preventing the formation of undesired toxic species such as bis(bromomethyl)ether. We re-disclosed the applicability of bromomethyl sulfides as precursors of lithiated organometallics and performed unprecedented free radical additions, that support the usefulness of these building blocks. More synthetic applications and derivatizations of bromomethyl sulfides are currently being developed in our laboratories.

## Conflicts of interest

There are no conflicts to declare.

## Supplementary Material

RA-008-C8RA04002H-s001
