# Supplementary material for: An expeditious and efficient bromomethylation of thiols: enabling bromomethyl sulfides as useful building blocks
Source: RSC Adv. 2018 Jul 10;8(43):24654–9. doi: 10.1039/c8ra04002h (PMC9092473; doi:10.1039/c8ra04002h)

Electronic Supplementary Material (ESI) for RSC Advances.
This journal is © The Royal Society of Chemistry 2018

An expeditious and efficient bromomethylation of thiols: enabling bromomethyl sulfides as useful building blocks

C. Silva-Cuevas,[1] E. Paleo,[2] D. F. León-Rayo,[1] and J. A. Luján-Montelongo[1]*

1. Departamento de Química, Centro de Investigación y de Estudios Avanzados del Instituto Politécnico Nacional (Cinvestav). Av. Instituto Politécnico Nacional 2508, San Pedro Zacatenco, 07360, México D. F., México.
jalujanm@cinvestav.mx

2. Facultad de Ciencias, Universidad Nacional Autónoma de México, Ciudad Universitaria, 04510, Ciudad de México, México.

All reagents and solvents were purchased from commercial sources, and were used as received, unless noted otherwise. Thin layer chromatography (TLC) qualitative analysis were performed using glass or aluminum backed silica gel plates (F_{254}). TLC plates were visualized by exposition to a UV light lamp (254 nm) and/or developed with a phosphomolybdic acid solution (20% in ethanol) or iodine. Flash column chromatography was performed on 230-400 mesh silica gel. Rotary chromatography was performed using a Chromatotron™ device, equipped with 1, 2, or 4 mm SiO_2 (high purity, 2-25 μm particle size) layer thickness glass rotors. ^{1}H NMR and ^{13}C NMR spectra were acquired on a Jeol Eclipse 270, Bruker DP300, Bruker Avance III HD 400, and ECA 500 spectrometers. ^{1}H NMR data is reported as follows: chemical shift, multiplicity (s = singlet, d = doublet or m = multiplet), coupling constants (Hz) and integration. Both ^{1}H and ^{13}C NMR data are reported in parts per million (ppm) on the δ scale. Infrared spectra were recorded on a Varian FT-IR 600IR spectrometer with an ATR sampling accessory. Mass spectra were obtained on an Agilent G1969A ESI-TOF or Jeol AccuTOF JMS-T100LC instruments. Elemental microanalysis was performed on a Thermo Finnigan FLASH EA 1112 series CHNS/O analyzer. **2a**, **2e**, **2s**, **5j**, **6e**, **8e**, and **8j** are known compounds with spectral data matching those previously reported. **2d**, **2f**, and **2k** molecular ions were confirmed by MS-DART+ and **2t**, **5e**, **6j**, **9e**, **9j**, **10e**, and **10j** molecular ions were confirmed by HRMS-ESI-QTOF or HRMS-DART+. Microanalysis confirmed elemental composition of **2o**. We couldn't gather elemental information for molecular ions of samples **2b-c**, **2g-j**, **2l-n**, and **2p-r**, and **2t-u** albeit $[M-Br]^+$ base peaks were present (HRMS-ESI-QTOF and/or HRMS-DART+).[1]

2. General procedure for the preparation of bromomethylsulfides from thiols.

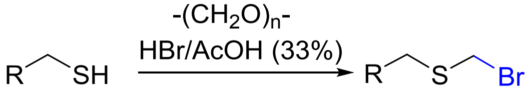

To a stirred mixture of the corresponding thiol **1** (1 equiv)[2] and paraformaldehyde (CAS: 30525-89-4) (1.0 equiv), a hydrogen bromide solution 33 wt. % in AcOH (Sigma-Aldrich Cat. No. 248630) (2.0 equiv) was added in one portion. The reaction mixture is stirred at rt[7a] until consumption of starting material (TLC).[3b] The **undiluted** acetic layer was extracted with hexanes (x3). The combined organic layers were dried over anh. Na_2SO_4 and concentrated *in vacuo*, to afford bromomethylsulfanyl derivative **2** with high purity (usually >95%). Analytical samples can be obtained by bulb-to-bulb vacuum distillation (Kugelrohr). Exceptional

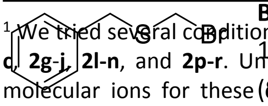

Benzyl(bromomethyl)sulfane (2a) was prepared from benzyl mercaptan **(1a)** (CAS: 100-53-8) (269 mg, 2.2 mmol), paraformaldehyde (65 mg, 2.2 mmol) and HBr/AcOH (800 μL, 4.4 mmol), in accordance to the general procedure (reaction time 10 min). The procedure affords 430 mg of **2a** (91%) as an oil. Spectral data match those previously reported.[4] ^{1}H NMR (400 MHz, $CDCl_3$) δ 7.37 – 7.26 (m, 5H), 4.43 (s, 2H), 3.89 (s, 2H). ^{13}C NMR (100 MHz, $CDCl_3$) δ 136.2, 129.4, 128.8, 127.7, 36.8, 35.9.

[1] We tried several conditions (HRMS ESI-QTOF and DART+) in order to detect molecular ions of bromomethyl sulfides **2b-c**, **2g-j**, **2l-n**, and **2p-r**. Unfortunately, base peaks consisting on $[M-Br]^+$ were found without detectable presence of molecular ions for these compounds. Despite this, characteristic ^{1}H NMR signals ($S-CH_2-Br$) had similar chemical shifts in all cases: 4.43-4.78 ppm for aliphatic bromomethyl sulfides, and 4.76-4.90 ppm for aromatic bromomethyl sulfides. Dithioacetal methylenes ($-S-CH_2-S-$) featured considerably lower chemical shifts with Δδ of *ca.* 1-0.5 ppm

[2] Typical scale: 0.5-5 mmol

[3] a) Some examples require higher or lower temperatures. b) Reaction time is substrate dependent, usually taking from 10 to 60 min.

[4] a) D. A. Evans, D. J. Mathre and W. L. Scott, *J. Org. Chem.*, 1985, **50**, 1830–1835. (b) H. J. Reich, C. P. Jasperse and J. M. Renga, *J. Org. Chem.*, 1986, **51**, 2981–2988.

[5] *rac*(1R,2S)-2-methoxycyclohexanethiol was prepared from cyclohexene oxide (CAS: 286-20-4), MeOH and conc. H_2SO_4.

1. General Experimental and Analytical Information.

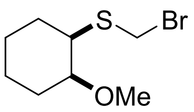

***rac*(Bromomethyl)((1R,2S)-2-methoxycyclohexyl)sulfane (2b)** was prepared from *rac*(1R,2S)-2-methoxycyclohexanethiol[5] **(1b)** (223 mg, 1.5 mmol), paraformaldehyde (45.8 mg, 1.5 mmol) and HBr/AcOH (560 µL, 3.1 mmol) in accordance to the general procedure (reaction time 30 min). The procedure affords 311 mg of **2b** (85%) as an oil. ^{1}H NMR (400 MHz, $CDCl_3$) δ 4.82 (d, *J* = 10.9 Hz, 1H), 4.71 (d, *J* = 10.9 Hz, 1H), 3.56 – 3.49 (m, 1H), 3.37 (s, 3H), 3.33 – 3.25 (m, 1H), 1.98 – 1.75 (m, 3H), 1.69 – 1.53 (m, 3H), 1.46 – 1.32 (m, 2H). ^{13}C NMR (100 MHz, $CDCl_3$) δ 79.3, 56.4, 48.6, 38.4, 28.7, 28.0, 23.9, 21.5.

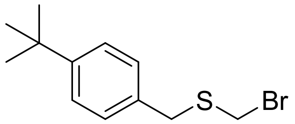

(Bromomethyl)(4-(tert-butyl)benzyl)sulfane (2c) was prepared from 4-tert-butylbenzyl mercaptan **(1c)** (CAS: 49543-63-7) (166.3 mg, 0.92 mmol), paraformaldehyde (27.7 mg, 0.92 mmol) and HBr/AcOH (340 µL, 1.87 mmol), in accordance to the general procedure (45 min reaction time). The procedure affords 253 mg of **2c** (quant.) as an oil. ^{1}H NMR (400 MHz, $CDCl_3$) δ 7.36 (d, *J* = 8.3 Hz, 2H), 7.28 (d, *J* = 8.3 Hz, 2H), 4.46 (s, 2H), 3.87 (s, 2H). ^{13}C NMR (100 MHz, $CDCl_3$) δ 150.7, 133.1, 129.1, 125.8, 37.1, 35.5, 34.7, 31.5.

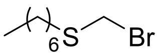

(Bromomethyl)(heptyl)sulfane (2d) was prepared from 1-heptanethiol **(1d)** (CAS: 1639-09-4)(315 mg, 2.4 mmol), paraformaldehyde (72 mg, 2.4 mmol) and HBr/AcOH (880 µL, 4.9 mmol), in accordance to the general procedure (reaction time 10 min). The procedure affords 533 mg of **2d** (99%) as an oil. ^{1}H NMR (300 MHz, $CDCl_3$) δ 4.67 (s, 2H), 2.74 (t, *J* = 7.4 Hz, 2H), 1.66 (p, *J* = 7.2 Hz, 2H), 1.43-1.23 (m, 8H), 0.90-0.87 (m, 3H). ^{13}C NMR (75 MHz, $CDCl_3$) δ 38.4, 32.9, 31.8, 29.0, 28.9, 28.3, 22.7, 14.2. MS (DART+), m/z 225, 227 (M+H).

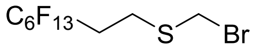

(Bromomethyl)(3,3,4,4,5,5,6,6,7,7,8,8,8-tridecafluorooctyl)sulfane (2e) was prepared from 3,3,4,4,5,5,6,6,7,7,8,8,8-tridecafluoro-1-octanethiol **(1e)** (CAS: 34451-26-8)(179 mg, 0.47 mmol), paraformaldehyde (14.1 mg, 0.47 mmol) and HBr/AcOH (170 µL, 0.94 mmol), in accordance to the general procedure (reaction time 20 min). The procedure affords 197 mg of **2e** (88%) as an oil, after Kugelrohr distillation (70 - 80 °C, 1.0 mmHg). Spectral data match those previously reported.[6] ^{1}H NMR (400 MHz, $CDCl_3$) δ 4.64 (s, 2H), 3.03 – 2.98 (m, 2H), 2.59 – 2.42 (m, 2H). ^{19}F NMR (375 MHz, TFA-*d*) δ -80.2 (tt, *J* = 10.1, 2.3 Hz, 3F), -113.6-113.8 (m, 2F), -121.2 (bs, 2F), 122.2 (bs, 2H), 122.7 (bs, 2H), 125.4 – 125.5 (m, 2H).

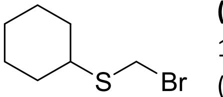

(Bromomethyl)(cyclohexyl)sulfane (2f) was prepared from cyclohexanethiol **(1f)** (CAS: 1569-69-3)(398 mg, 3.4 mmol), paraformaldehyde (103 mg, 3.4 mmol) and HBr/AcOH (1.26 mL, 7.0 mmol) in accordance to the general procedure (reaction time 10 min). The procedure affords 714 mg of **2f** (quant.) as an oil. ^{1}H NMR (300 MHz, $CDCl_3$) δ 4.71 (s, 2H), 3.12 - 2.84 (m, 1H), 2.14 - 1.94 (m, 2H), 1.87 - 1.71 (m, 2H), 1.72 - 1.57 (m, 1H), 1.47 – 1.23 (m, 5H). ^{13}C NMR (75 MHz, $CDCl_3$) δ 44.2, 36.6, 32.6, 25.9, 25.8. MS (DART+), m/z 209, 211 (M+H).

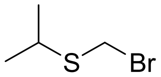

(Bromomethyl)(isopropyl)sulfane (2g) was prepared from 2-propanethiol **(1g)** (CAS: 75-33-2)(338 mg, 4.4 mmol), paraformaldehyde (133 mg, 4.4 mmol) and HBr/AcOH (1.63 mL, 9.0 mmol) in accordance to the general procedure (10 min reaction time)[7]. The procedure affords 471 mg of **2g** (63%) as an oil. ^{1}H NMR (500 MHz, $CDCl_3$) δ 4.70 (s, 2H), 3.22 (hept, *J* = 6.7 Hz, 1H), 1.34 (d, *J* = 6.7 Hz, 5H). ^{13}C NMR (125 MHz, $CDCl_3$) δ 36.8, 35.9, 22.4.

---

[6] F. Marty, E. Bollens, E. Rouvier and A. Cambon, *J. Fluor. Chem.*, 1990, **48**, 239–248.

[7] Due to its high volatility, **2f** was extracted with pentane.

[8](+)-Neomenthylthiol was prepared from *l*-menthol (CAS: 2216-51-5) in accordance to: J. M Blanco, O. Caamaño and F. Fernández *Tetrahedron* 1995, ***51***, 935-940.

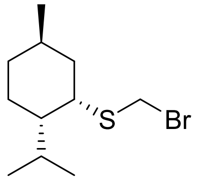

(Bromomethyl)((1*S*,2*S*,5*R*)-2-isopropyl-5-methylcyclohexyl)sulfane (2h) was prepared from (+)-neomenthylthiol[8] **(1h)** (363 mg, 2.1 mmol), paraformaldehyde (63 mg, 2.1 mmol) and HBr/AcOH (770 μL, 4.3 mmol) in accordance to the general procedure (10 min reaction time). The procedure affords 502 mg of **2h** (90%) as an oil. ^{1}H NMR (300 MHz, $CDCl_3$) δ 4.71 (s, 2H), 3.48 (bs, 1H), 2.05 (dq, J = 13.9, 3.2 Hz, 1H), 1.95 – 1.69 (m, 3H), 1.57 (m, 1H), 1.32 – 1.04 (m, 2H), 1.13 – 0.89 (m, 4H), 1.00 (d, *J* = 6.5 Hz, 3H), 0.90 (d, *J* = 6.5 Hz, 4H), 0.89 (d, J = 6.4 Hz, 3H). ^{13}C NMR (75 MHz, $CDCl_3$) δ 48.5, 47.5, 39.4, 38.5, 35.3, 29.9, 26.8, 26.6, 22.2, 21.3, 20.7.

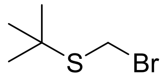

(Bromomethyl)(*tert*-butyl)sulfane (2i) was prepared with a modification of the general procedure: To a 1 M solution of 2-methyl-2-propanethiol **(1i)** (CAS: 75-66-1)(473.7 mg, 5.2 mmol) in hexanes, paraformaldehyde (158 mg, 5.2 mmol) was added and the mixture cooled to -20 °C. Subsequently, HBr/AcOH (1.93 mL, 10.7 mmol) was added in one portion and stirred (20 min). Isolation was carried out as described in the general procedure. The procedure affords 728 mg of **2i** (76%) as an orange oil. ^{1}H NMR (500 MHz, $CDCl_3$) δ 4.78 (s, 2H), 1.42 (s, 9H). ^{13}C NMR (125 MHz, $CDCl_3$) δ 45.1, 34.9, 30.7.

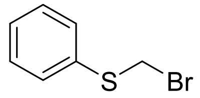

(Bromomethyl)(phenyl)(sulfane) (2j) was prepared from thiophenol **(1j)** (CAS: 108-98-5)(602 mg, 5.5 mmol), paraformaldehyde (164 mg, 5.5 mmol) and HBr/AcOH (2.0 mL, 11 mmol) in accordance to the general procedure (45 min reaction time). The procedure affords 1.086 g of **2j** (98%) as an oil. ^{1}H NMR (300 MHz, $CDCl_3$) δ 7.52 – 7.48 (m, 2H), 7.40 – 7.30 (m, 3H), 4.85 (s, 2H). ^{13}C NMR (75 MHz, $CDCl_3$) δ 133.4, 130.6, 129.4, 128.1, 37.8.

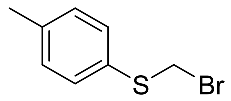

(Bromomethyl)(*p*-tolyl)sulfane (2k) was prepared from 4-methylbenzenethiol **(1k)** (CAS: 106-45-6)(327 mg, 2.6 mmol), paraformaldehyde (79 mg, 2.6 mmol) and HBr/AcOH (970 μL, 5.4 mmol) in accordance to the general procedure (45 min reaction time). The procedure affords 498 mg of **2k** (87%) as a slightly yellow oil after Kugelrohr distillation (60-65 °C, 0.5 mmHg). ^{1}H NMR (300 MHz, $CDCl_3$) δ 7.39 (d, *J* = 8.2 Hz, 2H), 7.17 (d, *J* = 7.9 Hz, 2H), 4.80 (s, 2H), 2.34 (s, 3H). ^{13}C NMR (75 MHz, $CDCl_3$) δ 138.5, 131.4, 130.1, 129.7, 39.0, 21.3. MS (DART+), m/z 217, 219 (M+H).

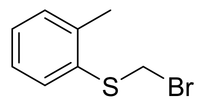

(Bromomethyl)(*p*-tolyl)sulfane (2l) was prepared from 2-methylbenzenethiol **(1l)** (CAS: 137-06-4)(216 mg, 1.74 mmol), paraformaldehyde (52 mg, 1.74 mmol) and HBr/AcOH (640 μL, 3.5 mmol) in accordance to the general procedure (45 min reaction time). The procedure affords 289 mg of **2l** (78%) as an oil after Kugelrohr distillation (135-140 °C, 10 mmHg). ^{1}H NMR (400 MHz, $CDCl_3$) δ 7.53 – 7.50 (m, 1H), 7.30 – 7.20 (m, 3H), 4.83 (s, 2H), 2.40 (s, 3H). ^{13}C NMR (100 MHz, $CDCl_3$) δ 139.0, 132.8, 130.7, 130.0, 128.0, 126.9, 37.1, 20.5.

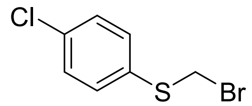

(Bromomethyl)(4-chlorophenyl)sulfane (2m) was prepared from 4-chlorothiophenol **(1m)** (CAS: 106-54-7)(448 mg, 3.1 mmol), paraformaldehyde (93 mg, 3.1 mmol) and HBr/AcOH (1.14 mL, 6.3 mmol) in accordance to the general procedure (45 min reaction time). The procedure affords 735 mg of **2m** (quant) as a yellow solid (mp 29-30 °C). ^{1}H NMR (300 MHz, $CDCl_3$) δ 7.44 (d, *J* = 8.6 Hz, 2H), 7.35 (d, *J* = 8.6 Hz, 2H), 4.81 (s, 2H). ^{13}C NMR (75 MHz, $CDCl_3$) δ 134.5, 132.2, 131.8, 129.6, 37.5.

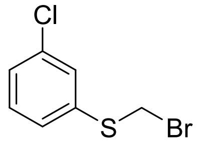

(Bromomethyl)(3-chlorophenyl)sulfane (2n) was prepared from 3-chlorothiophenol **(1n)** (CAS: 2037-31-2)(727 mg, 5.0 mmol), paraformaldehyde (151 mg, 5.0 mmol) and HBr/AcOH (1.85 mL, 10.2 mmol) in accordance to the general procedure (45 min

reaction time). The procedure affords 1.064 g of **2n** (89%) as a colorless oil. ^{1}H NMR (300 MHz, $CDCl_3$) δ 7.47 (s, 1H), 7.39 – 7.32 (m, 1H), 7.34 – 7.24 (m, 2H), 4.83 (s, 2H). ^{13}C NMR (75 MHz, $CDCl_3$) δ 135.4, 135.0, 130.4, 129.8, 128.2, 128.1, 36.4.

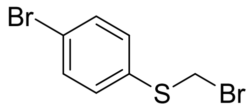

(Bromomethyl)(4-bromophenyl)sulfane (2o) was prepared from 4-bromothiophenol **(1o)** (CAS: 106-53-6)(172.1 mg, 0.9 mmol), paraformaldehyde (27 mg, 0.9 mmol) and HBr/AcOH (330 μL, 1.8 mmol) in accordance to the general procedure (45 min reaction time). The procedure to affords 249 mg of **2o** (97%) as a solid (mp 72-74 °C). ^{1}H NMR (400 MHz, $CDCl_3$) δ 7.51 (d, *J* = 8.7 Hz, 2H), 7.37 (d, *J* = 8.7 Hz, 2H), 4.81 (s, 2H). ^{13}C NMR (100 MHz, $CDCl_3$) δ 132.5, 132.3, 129.5, 122.5, 37.2. Found: C, 30.2; H, 2.2; S, 11.2; Br, 56.4. Calc. for $C_7H_6SBr_2$: C, 29.8; H, 2.1; S, 11.4; Br, 56.7%

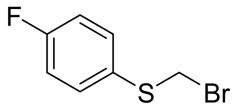

(Bromomethyl)(4-fluorophenyl)sulfane (2p) was prepared from 4-fluorothiophenol **(1p)** (CAS: 371-42-6)(302 mg, 2.4 mmol), paraformaldehyde (71 mg, 2.4 mmol) and HBr/AcOH (870 μL, 4.8 mmol) in accordance to the general procedure (45 min reaction time). The procedure to affords 496 mg of **2p** (95%) as an oil after Kugelrohr distillation (70-80 °C, 1.0 mmHg). ^{1}H NMR (300 MHz, $CDCl_3$) δ 7.51 (dd, *J* = 8.6, 5.3 Hz, 2H), 7.08 (t, *J* = 8.6 Hz, 2H), 4.78 (s, 2H). ^{13}C NMR (75 MHz, $CDCl_3$) δ 163.06 (d, *J* = 248.9 Hz), 134.02 (d, *J* = 8.4 Hz), 128.36 (d, *J* = 3.2 Hz), 116.57 (d, *J* = 22.1 Hz), 39.07.

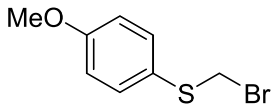

(Bromomethyl)(4-methoxyphenyl)sulfane (2q) was prepared from 4-methoxylthiophenol **(1q)** (CAS: 696-63-9)(328 mg, 2.3 mmol), paraformaldehyde (70 mg, 2.3 mmol) and HBr/AcOH (860 μL, 4.7 mmol) in accordance to the general procedure (60 min reaction time). The procedure affords 437 mg of **2q** (80%) as an oil after Kugelrohr distillation (95-100 °C, 1.0 mmHg). ^{1}H NMR (300 MHz, $CDCl_3$) δ 7.48 (d, *J* = 8.8 Hz, 2H), 6.91 (d, *J* = 8.8 Hz, 2H), 4.76 (s, 2H), 3.81 (s, 3H). ^{13}C NMR (75 MHz, $CDCl_3$) δ 160.4, 134.5, 123.7, 114.9, 55.5, 40.7.

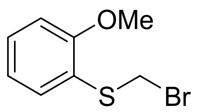

(Bromomethyl)(2-methoxyphenyl)sulfane (2r) was prepared with a modification of the general procedure: a 2-methoxythiophenol **(1r)** (CAS: 7217-59-6)(594 mg, 4.2 mmol), and paraformaldehyde (127 mg, 4.2 mmol) mixture was cooled to 0 °C before HBr/AcOH (1.56 mL, 8.6 mmol) addition (45 min reaction time). The procedure to affords 514 mg of **2r** (52%) as a colourless oil after Kugelrohr distillation (100-105 °C, 3.0 mmHg, decompose). ^{1}H NMR (500 MHz, $CDCl_3$) δ 7.47 (dd, *J* = 7.7, 1.6 Hz, 1H), 7.31 (ddd, *J* = 8.2, 7.5, 1.7 Hz, 1H), 6.99 (td, *J* = 7.6, 1.2 Hz, 1H), 6.90 (dd, *J* = 8.2, 1.1 Hz, 1H), 4.87 (s, 2H), 3.87 (s, 3H). ^{13}C NMR (125 MHz, $CDCl_3$) δ 157.9, 131.5, 129.5, 121.3, 120.9, 111.0, 55.9, 35.8.

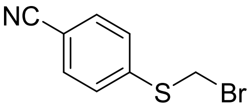

4-((bromomethyl)thio)benzonitrile (2s) was prepared from 4-cyanothiophenol[9] **(1s)** (118.8 mg, 0.87 mmol), paraformaldehyde (26 mg, 0.87 mmol) and HBr/AcOH (320 μL, 1.8 mmol) in accordance to the general procedure (45 min reaction time). The procedure to affords 93 mg of **2s** (47 %) as a white solid (mp 66-68 °C). Spectral data for this compound match those of the reported previously.[10] ^{1}H NMR (400 MHz, $CDCl_3$) δ 7.65 (d, *J* = 8.2 Hz, 2H), 7.51 (d, *J* = 8.1 Hz, 2H), 4.88 (s, 2H). ^{13}C NMR (100 MHz, $CDCl_3$) δ 140.8, 132.8, 128.3, 118.5, 110.6, 33.3.

---

[9] C. Silva-Cuevas, C. Perez-Arrieta, L. A. Polindara-García and J. A. Lujan-Montelongo, *Tetrahedron Lett.*, 2017, **58**, 2244–2247.

[10] A. Martel, C. Bachand and J.-D. Daris, Antibiotic C-3 dithioacetal-substituted carbapenem compounds, compositions, and use thereof. EP 0481511 A2. October 18, 1991.

[11] F. D. Toste, A. J. Lough and I. W. J. Still, *Tetrahedron Lett.* 1995, **36**, 6619-6622.

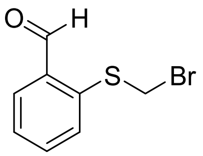

2-((Bromomethyl)thio)benzaldehyde (2t) was prepared with a modification of the general procedure: a 2-mercaptobenzaldehyde[11] **(1t)** (CAS: 29199-11-9)(256 mg, 1.85 mmol) and paraformaldehyde (556 mg, 1.85 mmol) mixture was heated to 30 °C before HBr/AcOH (670 µL, 3.7 mmol) addition (1 h reaction time). The procedure affords 258 mg of **2t** (60%) as an orangish solid with *ca.* 90% purity. An analytical sample can be obtained by partial crystallization from cold hexanes (25% recovered, mp 60 °C [decomp]). ^{1}H NMR (400 MHz, $CDCl_3$) δ 10.30 (s, 1H), 7.92 (dd, *J* = 7.6, 1.4 Hz, 1H), 7.72 – 7.65 (m, 2H), 7.48 (ddd, *J* = 8.3, 6.8, 1.8 Hz, 1H), 4.87 (s, 2H). ^{13}C NMR (100 MHz, $CDCl_3$) δ 191.56, 137.65, 134.44, 134.40, 133.10, 128.76, 127.12, 34.95. HRMS (ESI-QTOF) m/z: Calculated for C_8H_7OS $[M-Br]^+$ 151.0212; Found 151.0214.

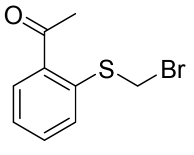

1-(2-((bromomethyl)thio)phenyl)ethan-1-one (2u) was prepared with a modification of the general procedure: a 1-(2-mercaptophenyl)ethan-1-one [12] **(1t)** (CAS: 29199-11-9)(54.5 mg, 0.36 mmol) and paraformaldehyde (11 mg, 0.36 mmol) mixture was heated to 40 °C before HBr/AcOH (670 µL, 3.7 mmol) addition (1 h reaction time). The procedure affords 35.1 mg of **2u** (60%) as a solid (88-90 °C). ^{1}H NMR (400 MHz, $CDCl_3$) δ 7.90 (dd, *J* = 7.8, 1.5 Hz, 1H), 7.68 (dd, *J* = 8.2, 1.2 Hz, 1H), 7.61 (ddd, *J* = 8.1, 7.2, 1.5 Hz, 1H), 7.34 (ddd, *J* = 7.8, 7.2, 1.2 Hz, 1H), 4.87 (s, 2H), 2.63 (s, 3H). ^{13}C NMR (100 MHz, $CDCl_3$) δ 199.21, 137.65, 135.07, 132.86, 131.34, 126.06, 125.43, 34.35, 28.19. ^{13}C NMR (100 MHz, $CDCl_3$) δ 199.21, 137.65, 135.07, 132.86, 131.34, 126.06, 125.43, 34.35, 28.19. HRMS (ESI-QTOF) m/z: Calculated for C_9H_9OS $[M-Br]^+$ 165.0369; Found 165.0368.

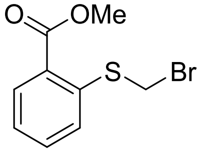

Methyl 2-((bromomethyl)thio)benzoate (2v) was prepared from methyl 2-mercaptobenzoate[13] **(1v)** (CAS: 4892-02-8)(100 mg, 0.6 mmol), paraformaldehyde (18 mg, 0.6 mmol) and HBr/AcOH (220 µL, 1.2 mmol), in accordance to the general procedure (45 min reaction time). The procedure affords 132 mg of **2t** (85%) as a slightly yellow solid. mp 114-116 °C. ^{1}H NMR (500 MHz, $CDCl_3$) δ 8.04 (d, *J* = 7.8 Hz, 1H), 7.64 (d, *J* = 7.9 Hz, 1H), 7.60 (t, *J* = 7.6 Hz, 1H), 7.30 (t, *J* = 7.5 Hz, 1H), 4.90 (s, 2H), 3.92 (s, 3H). ^{13}C NMR (125 MHz, $CDCl_3$) δ 138.3, 133.0, 131.7, 128.0, 125.8, 125.6, 52.5, 34.0. IR-ATR ν_{max}/cm^{-1} 1705 (C=O). HRMS (ESI-QTOF) m/z: Calculated for $C_9H_{10}O_2SBr$ $[M+H]^+$ 260.9579, 262.9558; Found 260.9578, 262.9560.

3. Distinctive ^{1}H-NMR chemical shifts (ppm) for bromomethyl sulfides (2i-v) and selected dithioacetals (3a-b, f-g, i-m, o-r, v).

Literature reported data is included within parentheses:

Aliphatics		-S-C**H$_2$**-Br		-S-C**H$_2$**-S-
	**2a**	4.43 (4.33)[8a]	**3a**	(3.37)[14]
	**2b**	4.82<br>4.71	**3b**	3.88
	**2c**	4.46		
	**2d**	4.67		
	**2e**	4.64 (4.56)[10]		

Aromatics		-S-C**H$_2$**-Br		-S-C**H$_2$**-S-
	**2j**	4.85	**3j**	(4.35)[4]
	**2k**	4.80	**3k**	4.24 (4.24)[2]
	**2l**	4.83	**3l**	4.29
	**2m**	4.81	**3m**	(4.28)[4]
	**2n**	4.83		
	**2o**	4.81	**3o**	(4.28)[4]

[12] M. J. Topolski, *J. Org. Chem.* 1995, **60**, 5588–5594.
[13] X. Du, H. Liu and D. M. Du, *Tetrahedron Asymmetry*, 2010, **21**, 241–246.
[14] K. Ajiki, M. Hirano and K. Tanaka, *Org. Lett.,* 2005, **7**, 4193-4195.

	**2f** 4.71	**3f**
	**2g** 4.70	**3g** (3.70)[15]
	**2h** 4.71	
	**2i** 4.78	**3i** (3.68)[3]

	**2p**	4.78	**3p**	(4.22)[4]
	**2q**	4.76	**3q**	4.16 (4.15)[16]
	**2r**	4.87	**3r**	4.35 (4.35)[17]
	**2s**	4.88 (4.88)[14]		
	**2t**	4.87		
	**2u**	4.87		
	**2v**	4.90	**3v**	(4.41)[5]

4. Procedures for the preparation of bromoalkylsulfides (2k1-3, 2a1) from thiols and selected carbonyl compounds.

Bromoalkylation of thiols were performed following the general procedure for the preparation of bromomethylsulfides from thiols. Dithioacetals (**3k1-3**, **3a3**) were synthesized following a two-fold amount of the thiol reactant following the general bromomethylation procedure. Bromoalkylsulfides (**2k1-3**, **2a1**) could not be purified by either distillation or chromatographic techniques and are reported as mixtures with dithioacetals (**3k1-3**, **3a3**).

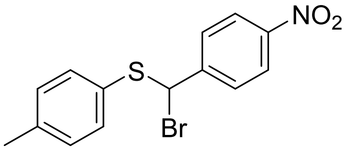

(Bromo(4-nitrophenyl)methyl)(*p*-tolyl)sulfane (2k1) was prepared from 4-methylbenzenethiol **(1k)** (CAS: 106-45-6) (229.9 mg, 1.85 mmol), 4-nitrobenzaldehyde (279.7 mg, 1.85 mmol) and HBr/AcOH (670 μL, 3.7 mmol) in accordance to the general bromomethylation procedure (16 h reaction time). The procedure affords 537 mg of a mixture consisting on **2k1** (67%) and dithioacetal **3k1** (9) as a yellow solid. **2k1**[18] ^{1}H NMR (400 MHz, $CDCl_3$) δ 8.20 (d, *J* = 8.2 Hz, 2H), 7.64 (d, *J* = 8.3 Hz, 2H), 7.41 (d, *J* = 8.3 Hz, 2H), 7.19 (d, *J* = 8.3 Hz, 2H), 6.22 (s, 1H), 2.37 (s, 3H). ^{13}C NMR (100 MHz, $CDCl_3$) δ 147.9, 146.6, 140.2, 134.1, 130.3, 128.6, 128.3, 124.1, 58.9, 21.5. **((4-Nitrophenyl)methylene)bis(*p*-tolylsulfane) (3k1)** ^{1}H NMR (400 MHz, $CDCl_3$) 8.05 (d, *J* = 8.3 Hz, 2H), 7.39 (d, *J* = 8.7 Hz, 2H), 7.22 (d, *J* = 8.1 Hz, 4H), 7.04 (d, *J* = 8.0 Hz, 4H), 5.33 (s, 1H), 2.29 (s, 6H). ^{13}C NMR (100 MHz, $CDCl_3$) δ 147.5, 147.2, 138.9, 133.8, 129.9, 129.6, 128.8, 123.6, 60.4, 21.3.

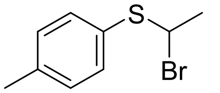

(1-Bromoethyl)(*p*-tolyl)sulfane (2k2) was prepared from 4-methylbenzenethiol **(1k)** (CAS: 106-45-6) (111.6 mg, 0.9 mmol), acetaldehyde (39.5 mg, 0.9 mmol) and HBr/AcOH (330 μL, 1.8 mmol) in accordance to the general procedure (1 h reaction time). The procedure affords 189.3 mg of a mixture consisting on **2k2** (56%) and **3k2** (36%) as a colorless oil. **2k2** ^{1}H NMR (400 MHz, $CDCl_3$) δ 7.45 (d, *J* = 8.1 Hz, 2H), 7.19 (d, *J* = 8.0 Hz, 2H), 5.43 (q, *J* = 6.8 Hz, 1H), 2.34 (s, 3H), 2.05 (d, *J* = 6.8 Hz, 3H). ^{13}C NMR (100 MHz, $CDCl_3$) δ 139.2, 133.7, 130.0, 129.2, 57.1, 27.8, 21.4. **Ethane-1,1-diylbis(p-tolylsulfane) (3k2)** ^{1}H NMR (400 MHz, $CDCl_3$) δ7.37 (d, *J* = 8.1 Hz, 2H), 7.10 (d, *J* = 8.1 Hz, 2H), 4.42 (q, *J* = 6.8 Hz, 1H), 2.32 (s, 3H), 1.55 (d, *J* = 6.9 Hz, 3H). ^{13}C NMR (100 MHz, $CDCl_3$) δ 138.0, 133.6, 130.4, 129.7, 52.9, 22.8, 21.3.

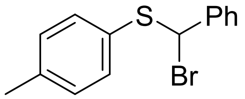

(Bromo(phenyl)methyl)(*p*-tolyl)sulfane (2k3) was prepared with a modification of the general procedure: a mixture of 4-methylbenzenethiol **(1k)** (CAS: 106-45-6)

[15] M. Prévost, S. Dostie, M. E. Waltz and Y. Guindon, *J. Org. Chem.* 2014, **79**, 10504–10525.
[16] V. Pace, A. Pelosi, D. Antermite, O. Rosati, M. Curini and W. Holzer, *Chem. Commun.,* 2016, **52**, 2639-2642.
[17] J. -J. Lee, H. -J. Jeong, C. -K. Jin, S. -H. Jang, S-H.; M. -K. Kim, Y. -J. Yoon and Lee, S. -G. *Bull. Korean Chem. Soc.*, 2005, **26**, 811-814.
[18] HETCOR and HMBC were used for assignment

(158.1 mg, 1.27 mmol) and benzaldehyde (135 mg, 1.27 mmol) was heated to 30°C, before HBr/AcOH (460 μL, 2.5 mmol) was added (16 h reaction time). The procedure affords 325 mg of a mixture consisting on **2k3** (46%) and **3k2** (29%) as a yellow solid. **2k3**[19] ^{1}H NMR (400 MHz, $CDCl_3$) 7.51 (d, *J* = 8.0, 1.7 Hz, 2H), 7.45 (d, *J* = 8.0 Hz, 2H), 7.33 – 7.31 (m, 3H), 7.17 (d, *J* = 7.7 Hz, 2H), 6.25 (s, 1H), 2.34 (s, 3H). ^{13}C NMR (100 MHz, $CDCl_3$) δ 139.9, 139.3, 133.4, 130.1, 129.2, 129.1, 128.8, 127.1, 62.2, 21.4. **(Phenylmethylene)bis(p-tolylsulfane) (3k3)** ^{1}H NMR (400 MHz, $CDCl_3$) δ 7.32 (dd, *J* = 8.0, 1.5 Hz, 2H), 7.27 – 7.20 (m, 7H), 7.04 (d, *J* = 8.0 Hz, 4H), 5.31 (s, 1H), 2.29 (s, 6H). ^{13}C NMR (100 MHz, $CDCl_3$) 140.1, 138.1, 133.3, 131.0, 129.7, 128.5, 128.0, 61.4, 21.3.

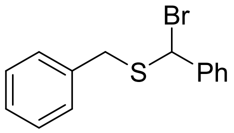

Benzyl(bromo(phenyl)methyl)sulfane (2a3) was prepared with a modification of the general procedure: a mixture of benzyl mercaptan **(1a)** (CAS: 100-53-8) (141.3 mg, 1.14 mmol) and benzaldehyde (120.7 mg, 1.14 mmol) was heated to 30°C, before HBr/AcOH (410 μL, 2.28 mmol) was added (1 h reaction time). The procedure affords 343 mg of a mixture consisting on **2a3** (61 %) and **3a2** (19 %) as an orange oil. **2a3**[18] ^{1}H NMR (400 MHz, $CDCl_3$) 7.44 – 72.8 (m, 10H), 5.87 (s, 1H), 4.01 (s, 2H) ^{13}C NMR (100 MHz, $CDCl_3$) δ 139.5, 136.4, 129.4, 129.3, 129.1, 128.85, 127.7, 127.2, 60.4, 39.0. **(Phenylmethylene)bis(benzylsulfane) (3a3)** δ 7.33 – 7.22 (m, 11H), 7.17 – 7.12 (m, 4H), 4.47 (s, 1H), 3.77 (d, *J* = 13.4 Hz, 2H), 3.55 (d, *J* = 13.4 Hz, 2H). ^{13}C NMR (100 MHz, $CDCl_3$) δ 139.7, 137.9, 129.1, 128.7, 128.6, 128.13, 128.0, 127.1, 51.0, 36.7.

5. **Distinctive ^{1}H-NMR chemical shifts (ppm) for bromoalkyl sulfides (2k1-3, 2a1) and selected dithioacetals (3k1-3, 3a3)**. Literature reported data is included within parentheses:

	R^1	R^2	-S-CHR-
**2k1**	*p*-Tol	*p*-$(NO_2)C_6H_4$	6.22
**2k2**	*p*-Tol	Me	5.43
**2k3**	*p*-Tol	Ph	6.25
**2a3**	Bn	Ph	5.87

	-S-CHR-S-
**3k1**	5.33 (5.34)[20]
**3k2**	4.42 (4.42)[21]
**3k3**	5.31 (5.31)[22]
**3a3**	3.77, 3.55 (3.7)[23]

6. **Procedures for bromo-lithium exchange / functionalization.**

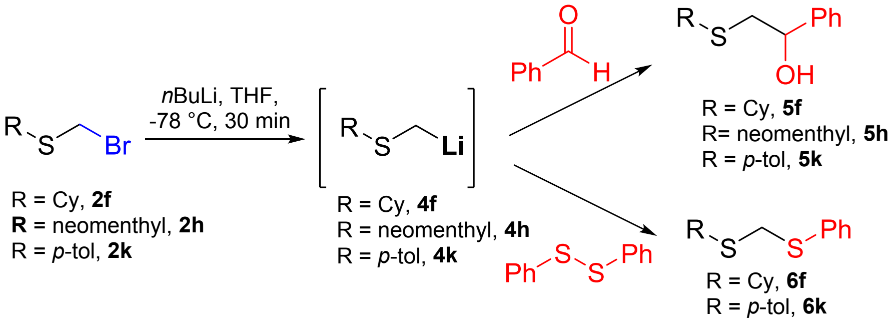

I. **Preparation of β-hydroxysulfides (5):** Under an argon atmosphere, a 1.6 M solution of BuLi (1.1 eq) was added dropwise to a cold (-78 °C) THF solution (0.6 M) of the

[19] HSQC and HMBC were used for assignment.
[20] S. Kumar, R. Kadu and S. Kumar, *Org. Biomol. Chem.*, 2016, **14**, 9210–9214.
[21] H. Xi, E. Ma and Z. Li, *Tetrahedron*, 2016, **72**, 4111–4116.
[22] H. Zhang, H. Wang, H. Yang and H. Fu, *Org. Biomol. Chem.*, 2015, **13**, 6149–6153.
[23] M. Kakimoto, T. Seri and Y. Imai, *Synthesis*, 1987, **1987**, 164–166.

corresponding bromomethyl sulfide (**2f**, **2h** or **2k**) (1 eq). After 15 min, a THF solution of benzaldehyde (CAS: 100-52-7) (1.1 M, 0.95 eq)[24] was added in one portion via syringe and stirred for 1 h at the same temperature (-78 °C). Subsequently, the reaction mixture was diluted with MeOH followed by half-saturated NH_4Cl solution and extracted with Et_2O (x4). The organic extracts were dried over Na_2SO_4, and the solvent removed *in vacuo* (no heating). The crude material was purified by radial chromatography using mixtures of hexanes/acetone or hexane/EtOAc.

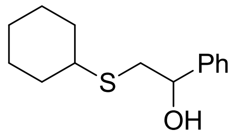

2-(cyclohexylthio)-1-phenylethan-1-ol (5f) was prepared from **2f** (259 mg, 1.2 mmol), BuLi 1.6 M (850 µL, 1.4 mmol) and benzaldehyde (125 mg, 1.2 mmol), in accordance to the general procedure for the preparation of β-hydroxysulfides. The procedure affords 208 mg of **5f** (71%) as a colorless liquid. ^{1}H NMR (400 MHz, $CDCl_3$) δ 7.39 – 7.30 (m, 4H), 7.31 – 7.21 (m, 1H), 4.68 (ddd, *J* = 9.3, 3.7, 2.6 Hz, 1H), 3.16 (d, *J* = 2.5 Hz, 1H), 2.95 (dd, *J* = 13.7, 3.8 Hz, 1H), 2.70 (dd, *J* = 13.7, 9.3 Hz, 1H), 2.66 – 2.59 (m, 1H), 2.02 – 1.90 (m, 2H), 1.81 – 1.70 (m, 2H), 1.65 – 1.54 (m, 1H), 1.41 – 1.17 (m, 5H). ^{13}C NMR (100 MHz, $CDCl_3$) δ 142.8, 128.5, 127.8, 125.8, 72.1, 43.7, 40.2, 33.9, 33.7, 26.1, 25.8. HRMS (ESI-QTOF) m/z: Calculated for $C_{14}H_{20}OSNa$ $[M+Na]^+$ 259.1127; Found 259.1128.

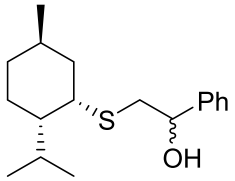

2-(((1*S*,2*S*,5*R*)-2-isopropyl-5-methylcyclohexyl)thio)-1-phenylethan-1-ol (5h) was prepared from **2h** (351 mg, 1.3 mmol), BuLi 1.6 M (910 µL, 1.4 mmol) and benzaldehyde (154 mg, 1.4 mmol), in accordance to the general procedure for the preparation of β-hydroxysulfides. The procedure affords 299.3 mg of the diastereoisomeric mixture of **5h** (***dr*** 1.4 : 1 as determined by ^{1}H-NMR, 77%) as a slightly yellow liquid. HRMS (ESI-QTOF) m/z: Calculated for $C_{18}H_{28}OSNa$ $[M+Na]^+$ 315.1753; Found 315.1752. Mixture of diastereoisomers: ^{1}H NMR (500 MHz, $CDCl_3$) δ δ 7.40 – 7.31 (m, 8H), 7.31 – 7.26 (m, 2H), 4.74 – 4.67 (m, 1H), 3.28 – 3.24 (m, 1H), 3.22 – 3.18 (m, 1H), 3.15 (d, *J* = 1.9 Hz, 1H), 3.10 (d, *J* = 2.3 Hz, 1H), 2.95 (dd, *J* = 10.3, 3.5 Hz), 2.91 (dd, *J* = 10.3, 3.2 Hz), 2.71 (dd, *J* = 13.3, 9.6 Hz), 2.66 (dd, *J* = 13.7, 10.0 Hz, 1H), 2.02 – 1.87 (mz, 4H), 1.78 – 1.63 (m, 7H), 1.31 – 1.03 (m, 7H), 0.96 (d, *J* = 6.6 Hz, 6H), 0.95 (d, *J* = 6.7 Hz, 6H), 0.91 (d, *J* = 6.6 Hz, 6H). ^{13}C NMR (125 MHz, $CDCl_3$) δ 142.80, 142.66, 128.60, 127.89, 127.88, 125.91, 125.89, 72.42, 71.13, 49.28, 48.79, 48.57, 45.91, 43.13, 42.14, 41.31, 40.06, 35.46, 35.39, 30.11, 30.04, 26.76, 26.32, 25.95, 25.90, 22.34, 22.31, 21.21, 21.15, 21.12, 20.88.

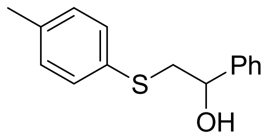

1-Phenyl-2-(*p*-tolylthio)ethan-1-ol (5k) was prepared from **2k** (144 mg, 0.66 mmol), BuLi 1.6 M (460 µL, 0.73 mmol) and benzaldehyde (67 mg, 0.63 mmol), in accordance to the general procedure for the preparation of β-hydroxysulfides. The procedure affords 125 mg of **5k** (77%) as a colorless liquid. Spectral data for this compound match those of the reported previously.[25] ^{1}H NMR (400 MHz, $CDCl_3$) δ 7.36 – 7.31 (m, 6H), 7.33 – 7.24 (m, 1H), 7.13 (d, *J* = 7.8 Hz, 2H), 4.67 (d, *J* = 9.5 Hz, 1H), 3.27 (dd, *J* = 13.8, 3.3 Hz, 1H), 3.02 (dd, *J* = 13.8, 9.7 Hz, 1H), 2.91 (s, 1H), 2.33 (s, 3H). ^{13}C NMR (100 MHz, $CDCl_3$) δ 142.3, 137.3, 131.2, 131.0, 130.1, 128.7, 128.0, 126.0, 71.6, 45.0, 21.2.

II. **Preparation of unsymmetrical dithioacetals (6):** Under an argon atmosphere, a 1.6 M solution of BuLi (1.1 eq) was added dropwise to a cold (-78 °C) THF solution (0.6 M) of the corresponding bromomethyl sulfide (**2f** or **2k**) (1 eq). After 15 min, a THF solution of phenyl disulfide (CAS: 882-33-7)(1.1 M, 0.95 eq) was added in one portion via

[24] 1.1 eq (1.1 M solution in THF) of benzaldehyde for the electrophilic quench in the case of **2h.**
[25] C. Huo, Y. Wang, Y. Yuan, F. Chen and J. Tang, *Chem. Commun.* 2016, **52**, 7233-7236.

syringe and stirred for 1h at the same temperature (-78 °C). Subsequently, the reaction mixture was diluted with MeOH and a half-saturated NH_4Cl solution, and extracted with Et_2O (x4). The organic extracts were dried over Na_2SO_4, and the solvent removed *in vacuo*. The crude material was purified by radial chromatography using mixtures of hexanes/acetone (99:1 to 98:2). In all cases, butyl(phenyl)sulfane was obtained as a by-product (from the condensation between butyl bromide and thiophenolate), which spectral data matching those previously reported.[26]

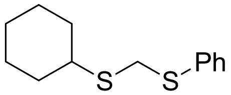

Cyclohexyl((phenylthio)methyl)sulfane (6f) was prepared from **2f** (291 mg, 1.4 mmol), BuLi 1.6 M (960 µL, 1.5 mmol) and phenyl disulfide (289 mg, 1.3 mmol). The procedure affords 188 mg of **6f** (60% yield) as a syrup. Spectral data for this compound match those of the reported previously.[27] ^{1}H NMR (400 MHz, $CDCl_3$) δ 7.41 (d, *J* = 8.0 Hz, 2H), 7.31 (t, *J* = 7.6 Hz, 2H), 7.25 – 7.20 (m, 1H), 4.05 (s, 2H), 3.03 – 2.83 (m, 1H), 2.06 – 1.90 (m, 2H), 1.87 – 1.70 (m, 2H), 1.69 – 1.54 (m, 1H), 1.48 – 1.15 (m, 5H). ^{13}C NMR (100 MHz, $CDCl_3$) δ 135.9, 130.4, 129.1, 126.9, 43.4, 36.3, 33.4, 26.1, 25.9.

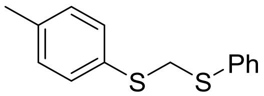

Phenyl((*p*-tolylthio)methyl)sulfane (6k) was prepared from **2k** (203 mg, 0.93 mmol), BuLi 1.6 M (640 µL, 1.03 mmol) and 1,2-diphenyldisulfane (194 mg, 0.89 mmol). The procedure affords 126 mg of **6k** (58% yield) as a syrup. ^{1}H NMR (400 MHz, $CDCl_3$) δ 7.42 (d, *J* = 8.1 Hz, 2H), 7.36 – 7.28 (m, 4H), 7.23 (t, *J* = 7.1 Hz, 1H), 7.13 (d, *J* = 8.1 Hz, 2H), 4.30 (s, 2H), 2.34 (s, 3H). ^{13}C NMR (100 MHz, $CDCl_3$) δ 137.7, 135.3, 131.8, 131.3, 130.7, 129.9, 129.1, 127.2, 41.5, 21.3.

7. Alternative preparation of unsymmetrical dithioacetals 6f and 6k

To a cold (0 °C) suspension of NaH (60% in mineral oil, 1 eq) in dry THF (0.5 M), thiophenol (1.0 eq) was added dropwise via syringe. After 30 min, bromomethyl sulfide **2f** or **2k** (1.1 eq) solution in dry THF (0.5 M) was added in one portion and stirred for 1 h at the same temperature. Afterwards, the reaction mixture was diluted with cold water and extracted with EtOAc (x4). The organic extracts were dried over Na_2SO_4, and the solvent removed *in vacuo.* Dithioacetals **6f** or **6k** were purified by column chromatography usingmixtures of hexanes/EtOAc. Yields: **6f** 97%, **6k** 85%.

8. Procedures for free radical reactions

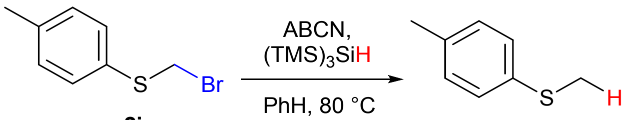

2j **8j**

I. Reduction – Preparation of thioether methyl(p-tolyl)sulfane (8j): a degassed solution of bromomethylsulfide (**2j**) (112 mg, 0.52 mmol), tris(trimethylsilyl)silane (CAS: 1873-77-4) (154 mg, 0.62 mmol), and 1,1'-azobis(cyclohexanecarbonitrile) (ABCN, CAS: 2094-98-6) (12.6 mg, 0.05 mmol) in dry benzene (5 mL), was refluxed for 30 min. After this, the reaction mixture was cooled down (rt), and concentrated *in vacuo.* The crude mixture

[26] A. M. Thomas, S. Asha, K. S. Sindhu and G. Anilkumar, *Tetrahedron Lett.*, 2015, **56**, 6560–6564.
[27] K. Sipilä, T. Hase, J. Koskimies, J. Matikainen and J. Kansikas, *Phosphorus, Sulfur Silicon Relat. Elem.*, 2002, **177**, 709–727.

was directly analyzed by ^{1}H-NMR using mesitylene as an internal standard to estimate a 85% yield.[28]

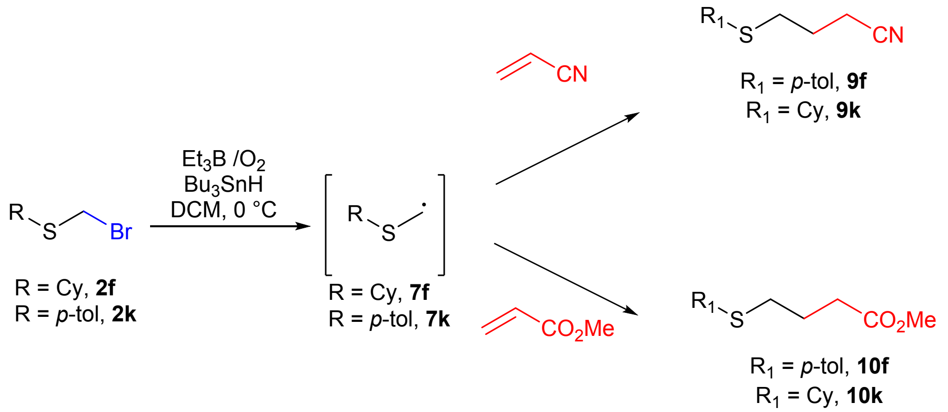

II. Radical additions - Preparation of γ-sulfenylnitriles (9f, 9k) and ethyl γ-sulfenylbutyrates (10f, 10k): to a dry, cold (0 °C) solution of the corresponding bromomethylsulfide **2f** or **2k** (1 eq), acrylonitrile (CAS: 107-13-1) or methyl acrylate (CAS: 96-33-3) (2 equiv) in DCM (0.01 M), a 1 M Et_3B solution in hexanes (0.2 eq) was added using a syringe and air was bubbled simultaneously. After this, a freshly prepared solution of Bu_3SnH (1.25 eq) in dry benzene (0.2 M) were added through a 2 h lapse (syringe pump), at 0 °C. Through the Bu_3SnH addition, additional Et_3B (0.2 eq) was added every 20 min with additional air bubbling (1.4 eq total). Afterwards, the reaction mixture was allowed to warm until rt and stirred for 1 h. Next, solvent was removed (no vacuum), and the resulting material was filtered on silica gel and purified by radial chromatography using mixtures of hexanes : EtOAc.

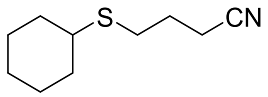

4-(Cyclohexylthio)butanenitrile (9f) was prepared from **2f** (190 mg, 0.91 mmol), acrylonitrile (120 μL, 1.82 mmol), Bu_3SnH 0.2 M in dry benzene (5.7 mL, 1.14 mmol), and Et_3B 1M in hexanes (1.27 mL, 1.27 mmol). The procedure affords 84.2 mg of **9f** (51%) as an oil. ^{1}H NMR (400 MHz, $CDCl_3$) δ 2.69 – 2.61 (m, 3H), 2.51 (td, J = 7.1, 1.4 Hz, 2H), 1.99 – 1.87 (m, 4H), 1.80 – 1.72 (m, 2H), 1.66 – 1.59 (m, 1H), 1.38 – 1.19 (m, 5H). ^{13}C NMR (100 MHz, $CDCl_3$) δ 119.2, 43.5, 33.6, 28.5, 26.0, 25.7, 25.6, 16.0. IR-ATR ν_{max} (cm^{-1}) 2246 (CN). HRMS (DART) m/z: Calculated for $C_{10}H_{18}NS$ $[M+H]^+$ 184.1160; Found 184.1164.

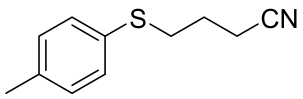

4-(*p*-Tolylthio)butanenitrile (9k) was prepared from **2k** (206 mg, 0.95 mmol), acrylonitrile (130 μL, 1.90 mmol), Bu_3SnH 0.2 M in dry benzene (1.19 mmol, 5.9 mL), and Et_3B 1M in hexanes (1.33 mL, 1.33 mmol). The procedure affords 145.4 mg of **9k** (80%) as a clear oil. ^{1}H NMR (400 MHz, $CDCl_3$) δ 7.26 (dd, J = 8.2, 2.3 Hz, 2H), 7.10 (dd, J = 8.2, 2.2 Hz, 2H), 3.0 – 2.90 (m, 2H), 2.5 – 2,43 (m, 2H), 2.31 (s, 3H),

[28] Reaction profiles featured clean conversions. Yield estimation using an internal standard was needed due a high volatility of the product and similar polarity of $(TMS)_3SiBr$. Spectral data of the product match those already reported in the literature: T. H. Chuo, R. Boobalan and C. Chen, *ChemistrySelect*, 2016, **1**, 2174–2180.

1.89 (qd, J = 7.0, 1.7 Hz, 2H). ^{13}C NMR (100 MHz, $CDCl_3$) δ 137.0, 130.93, 130.85, 129.9, 119.2, 33.2, 24.9, 21.0, 15.8. IR-ATR ν_{max} (cm^{-1}) 2246 (CN). HRMS (ESI-QTOF) m/z: Calculated for $C_{11}H_{14}NS$ $[M+H]^+$ 192.0847; Found 192.0828.

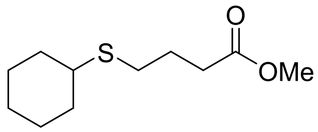

Methyl 4-(cyclohexylthio)butanoate (10f) was prepared from **2f** (171.3 mg, 0.82 mmol), methyl acrylate (150 μL, 1.64 mmol), Bu_3SnH 0.2 M in dry benzene (5.1 mL), and Et_3B 1M in hexanes (1.15 mL, 1.15 mmol). The procedure affords 64 mg of **10f** (36%) as an oil. ^{1}H NMR (400 MHz, $CDCl_3$) δ 3.68 (s, 3H), 2.68 – 2.61 (m, 1H), 2.57 (t, J = 7.3 Hz, 2H), 2.44 (t, J = 7.4 Hz, 2H), 2.00 – 1.86 (m, 4H), 1.80 – 1.71 (m, 2H), 1.65 – 1.57 (m, 1H), 1.37 – 1.19 (m, 5H). ^{13}C NMR (100 MHz, $CDCl_3$) δ 173.7, 51.6, 43.4, 33.8, 33.00, 29.4, 26.2, 25.9, 25.2. IR-ATR ν_{max} (cm^{-1}) 1737 (C=O). HRMS (DART+) m/z: Calculated for $C_{11}H_{21}O_2S$ $[M+H]^+$ 217.1262; Found 217.1275.

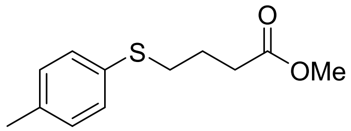

Methyl 4-(*p*-tolylthio)butanoate (10k) was prepared from **2k** (181 mg, 0.83 mmol), methyl acrylate (150 μL, 1.67 mmol), Bu_3SnH 0.2 M in dry benzene (5.2 mL), and Et_3B 1M in hexanes (1.17 mL). The procedure affords 104.2 mg of **10k** (56%) as an oil. ^{1}H NMR (500 MHz, $CDCl_3$) δ 7.25 (d, J = 10.5 Hz, 2H), 7.09 (d, J = 8.3 Hz, 2H), 3.66 (s, 3H), 2.91 (t, J = 7.1 Hz, 2H), 2.46 (dd, J = 7.3 Hz, 2H), 2.31 (s, 3H), 1.92 (p, J = 7.2 Hz, 2H). ^{13}C NMR (125 MHz, $CDCl_3$) δ 173.6, 136.4, 132.2, 130.4, 129.8, 51.7, 33.8, 32.7, 24.5, 21.1. IR-ATR ν_{max} (cm^{-1}) 1734 (C=O). HRMS (DART) m/z: Calculated for $C_{12}H_{17}O_2S$ $[M+H]^+$ 225.0949; Found 225.0964.

Benzyl(bromomethyl)sulfane (2a)

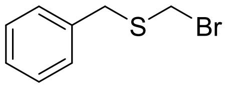

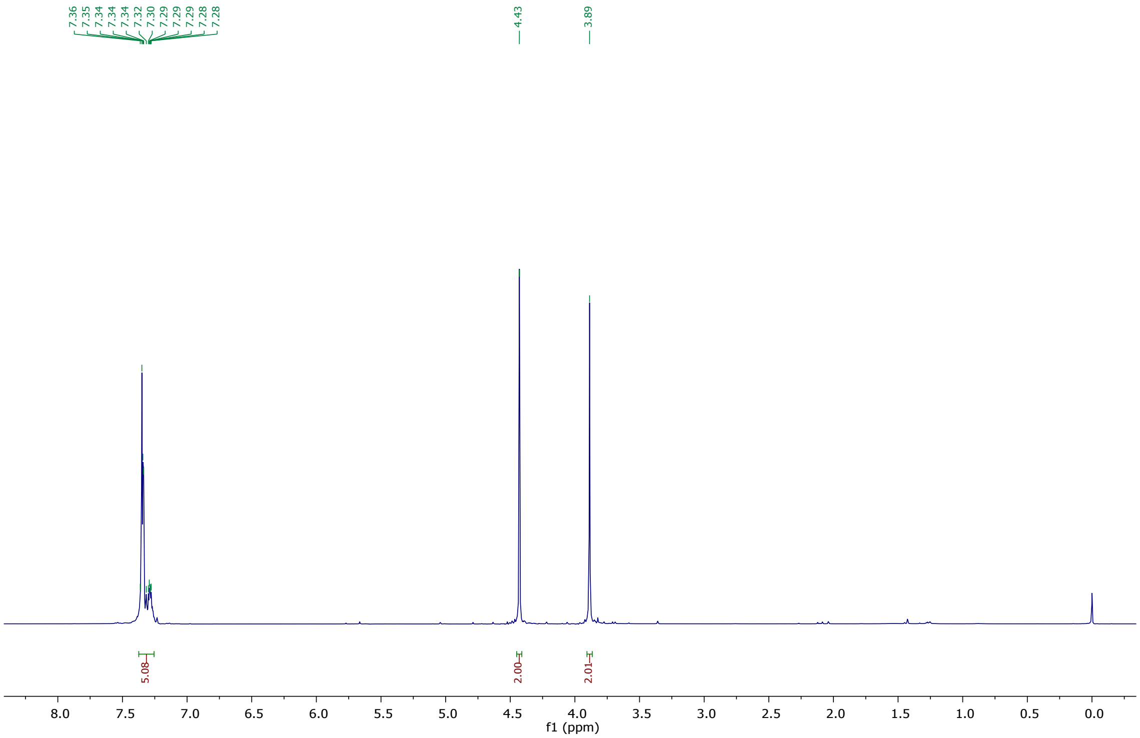

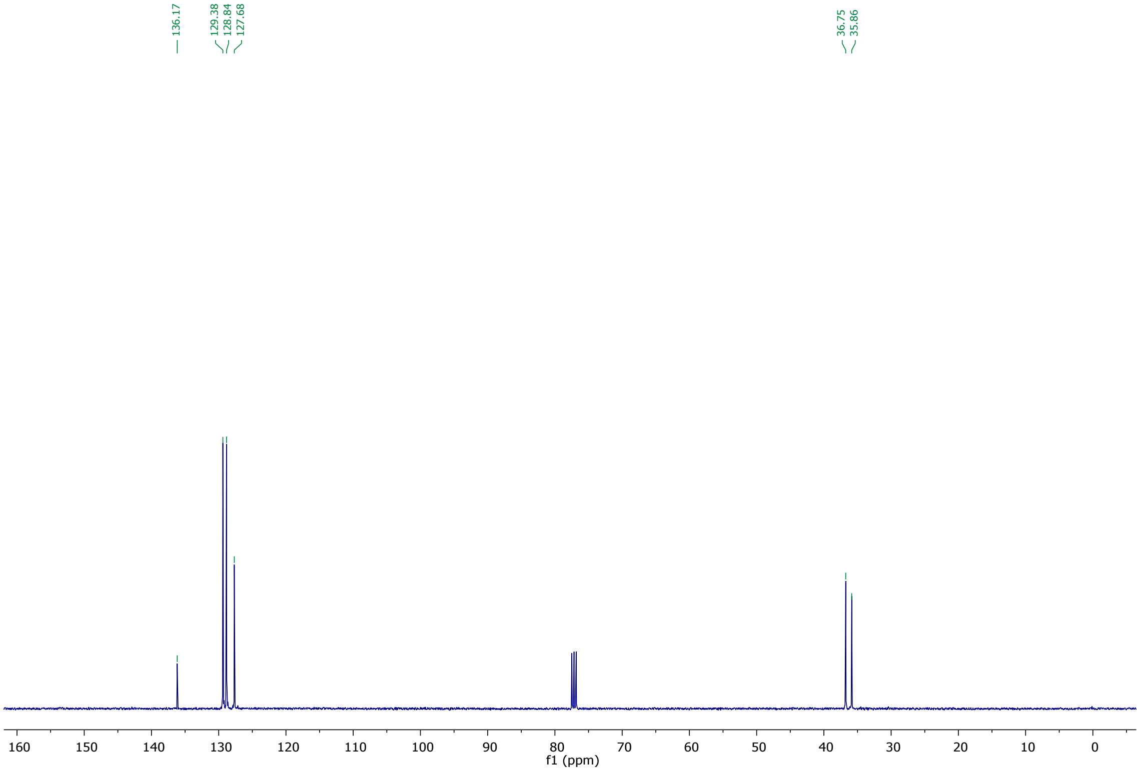

rac(Bromomethyl)((1R,2S)-2-methoxycyclohexyl)sulfane (2b)

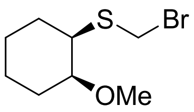

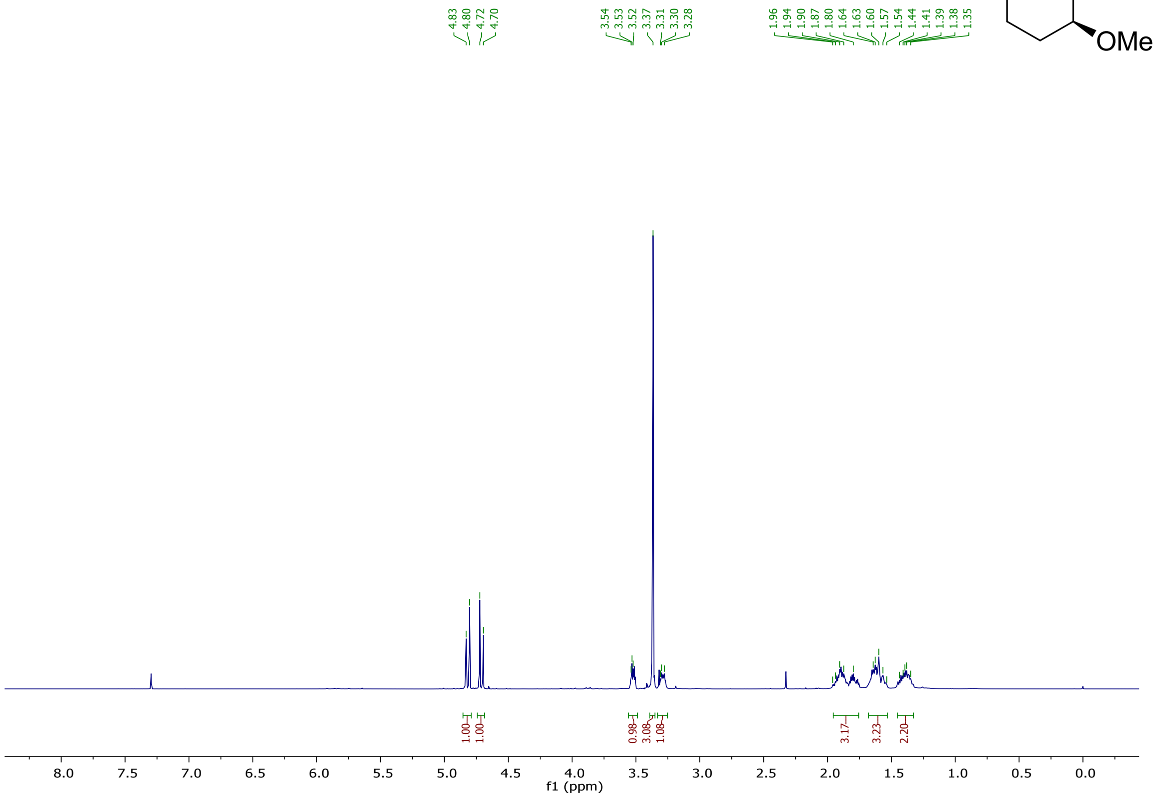

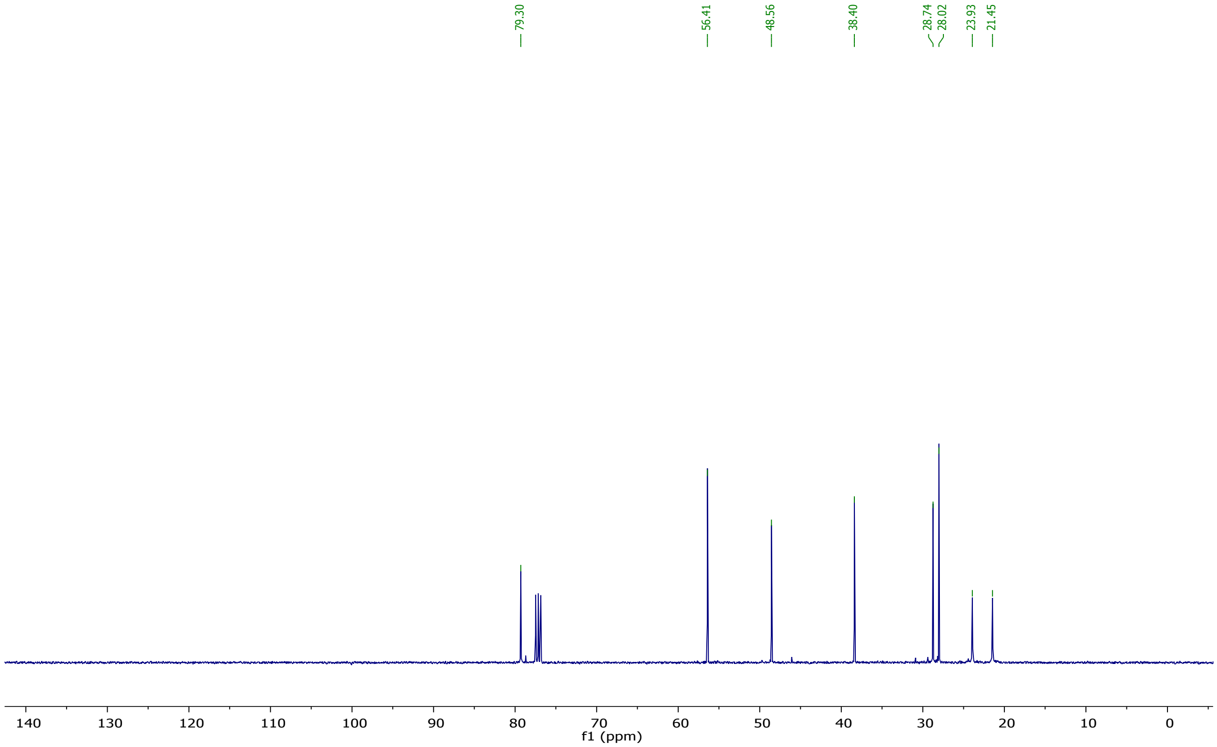

(Bromomethyl)(4-(tert-butyl)benzyl)sulfane (2c)

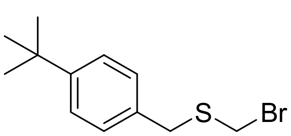

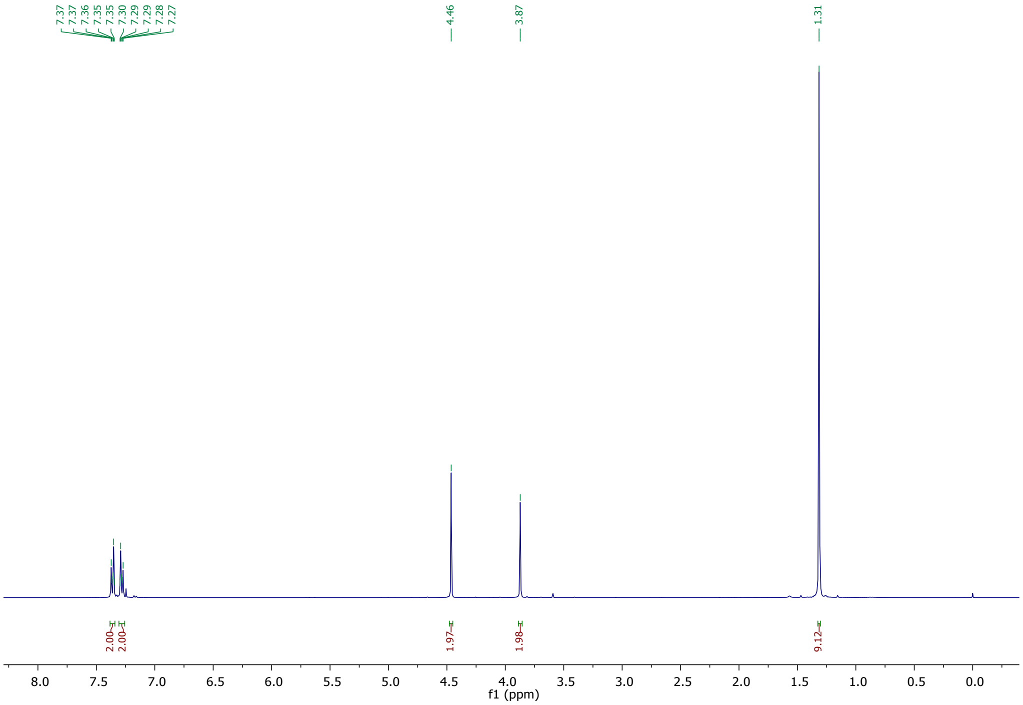

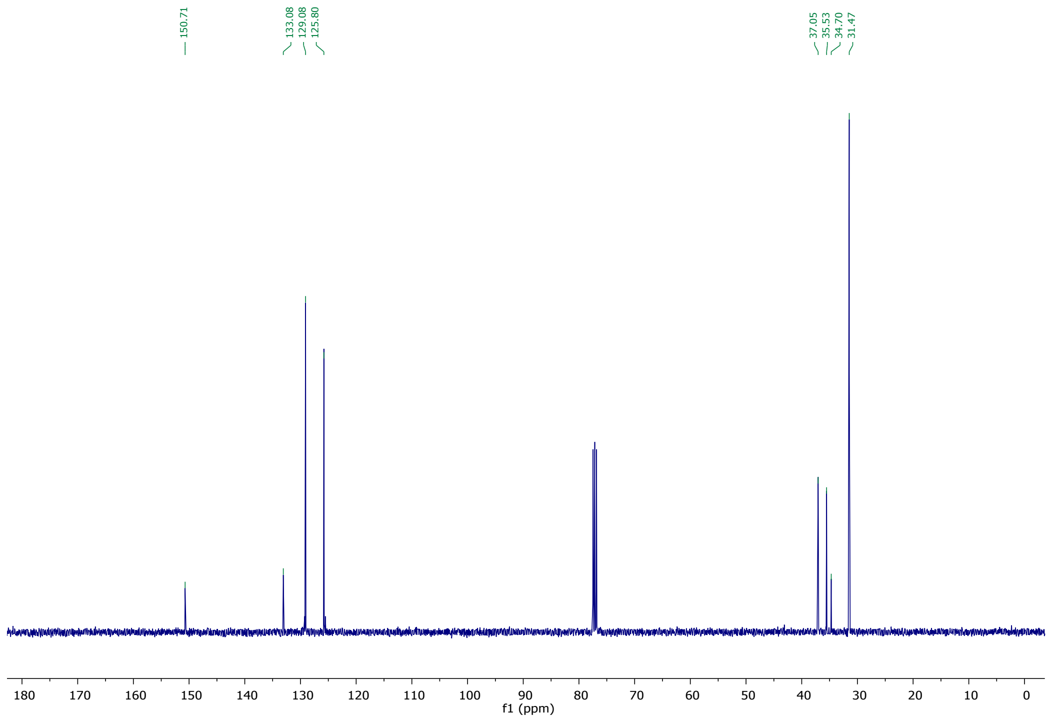

(Bromomethyl)(heptyl)sulfane (2d)

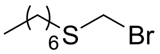

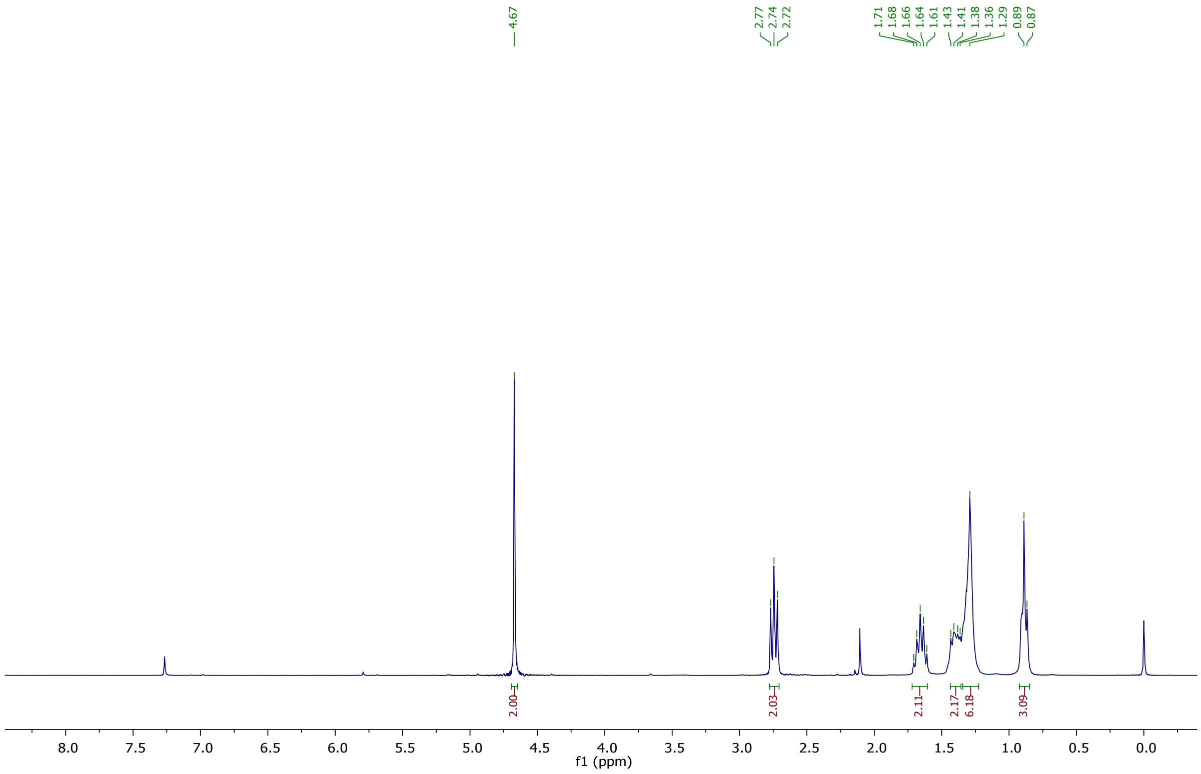

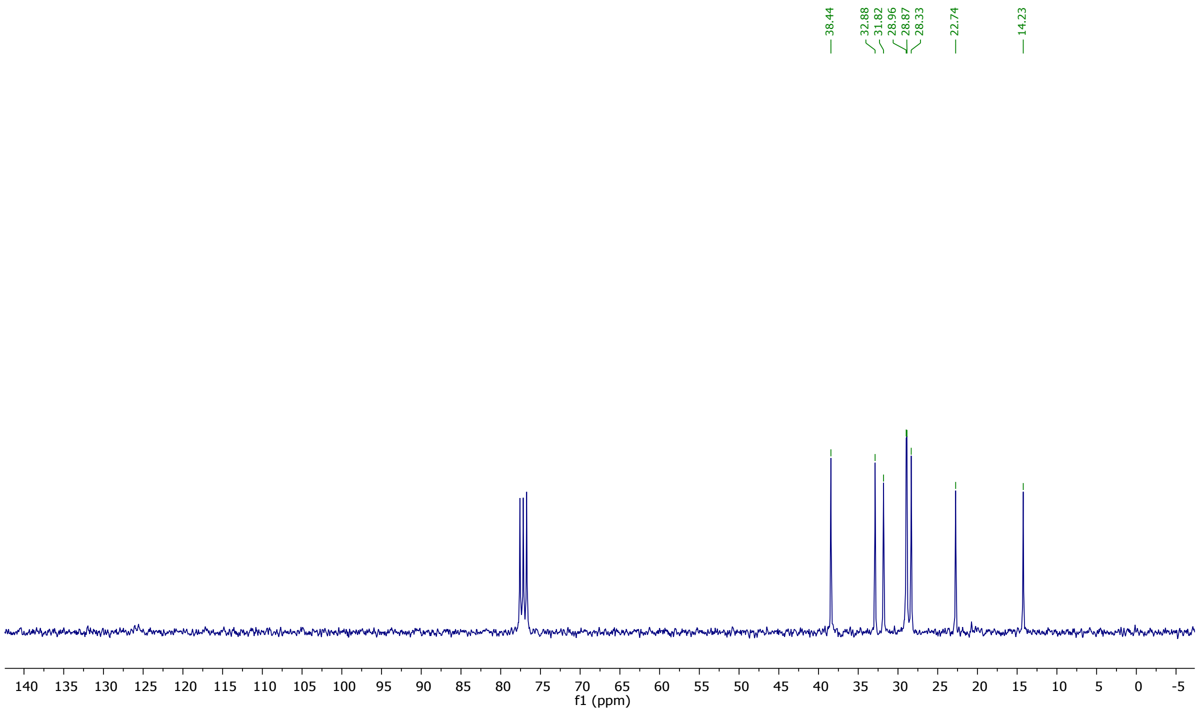
38.44
32.88
31.82
28.96
28.87
28.33
22.74
14.23
140 135 130 125 120 115 110 105 100 95 90 85 80 75 70 65 60 55 50 45 40 35 30 25 20 15 10 5 0 -5
f1 (ppm)

(Bromomethyl)(3,3,4,4,5,5,6,6,7,7,8,8,8-tridecafluorooctyl)sulfane (2e)

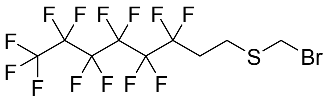

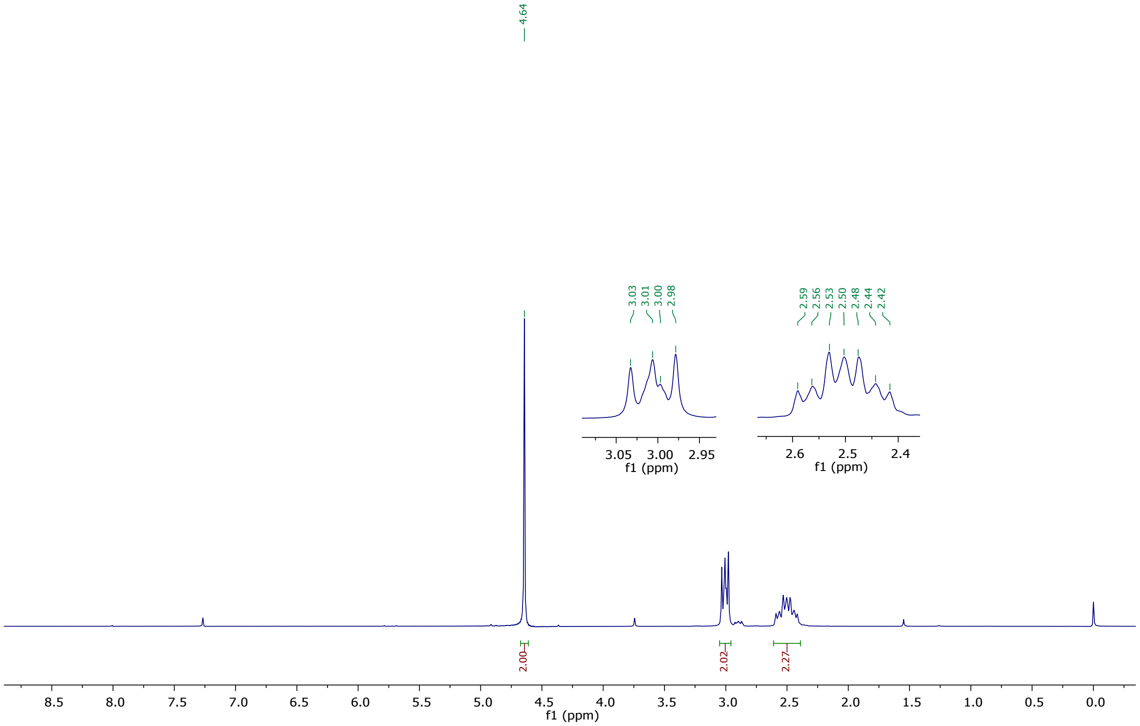

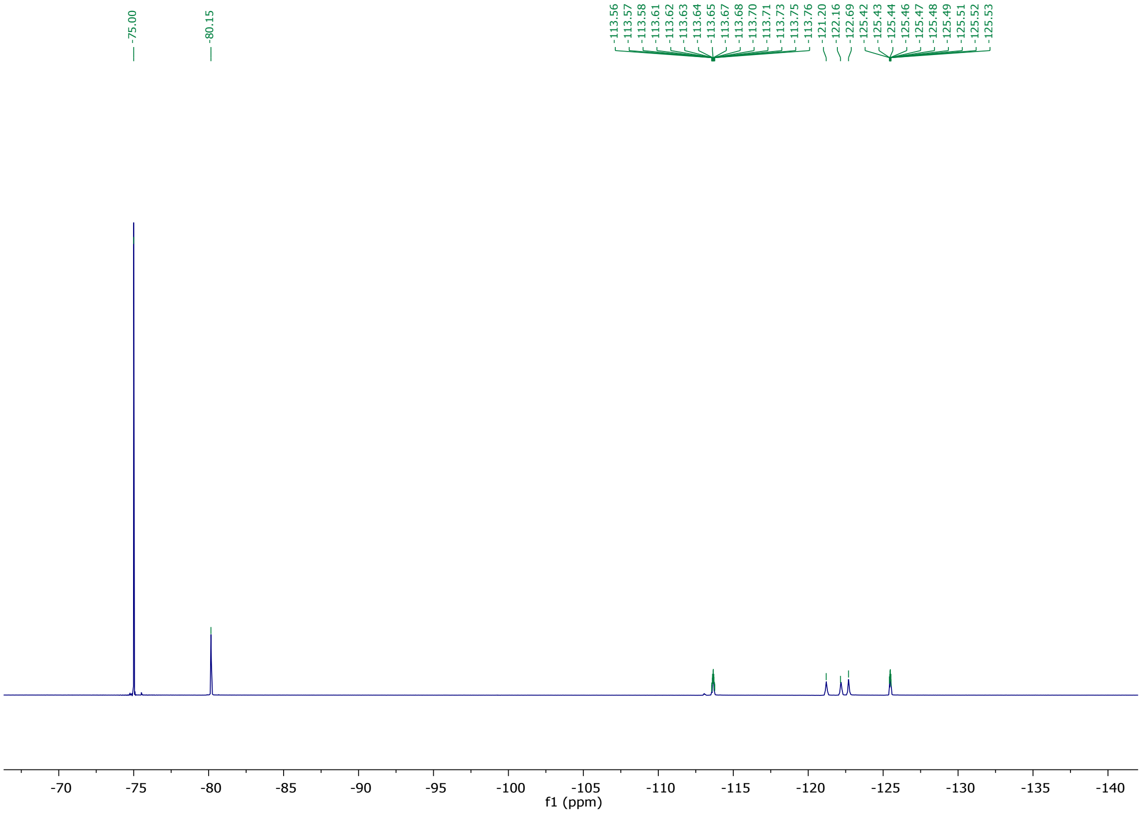

(Bromomethyl)(cyclohexyl)sulfane (2f)

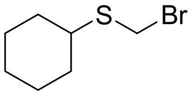

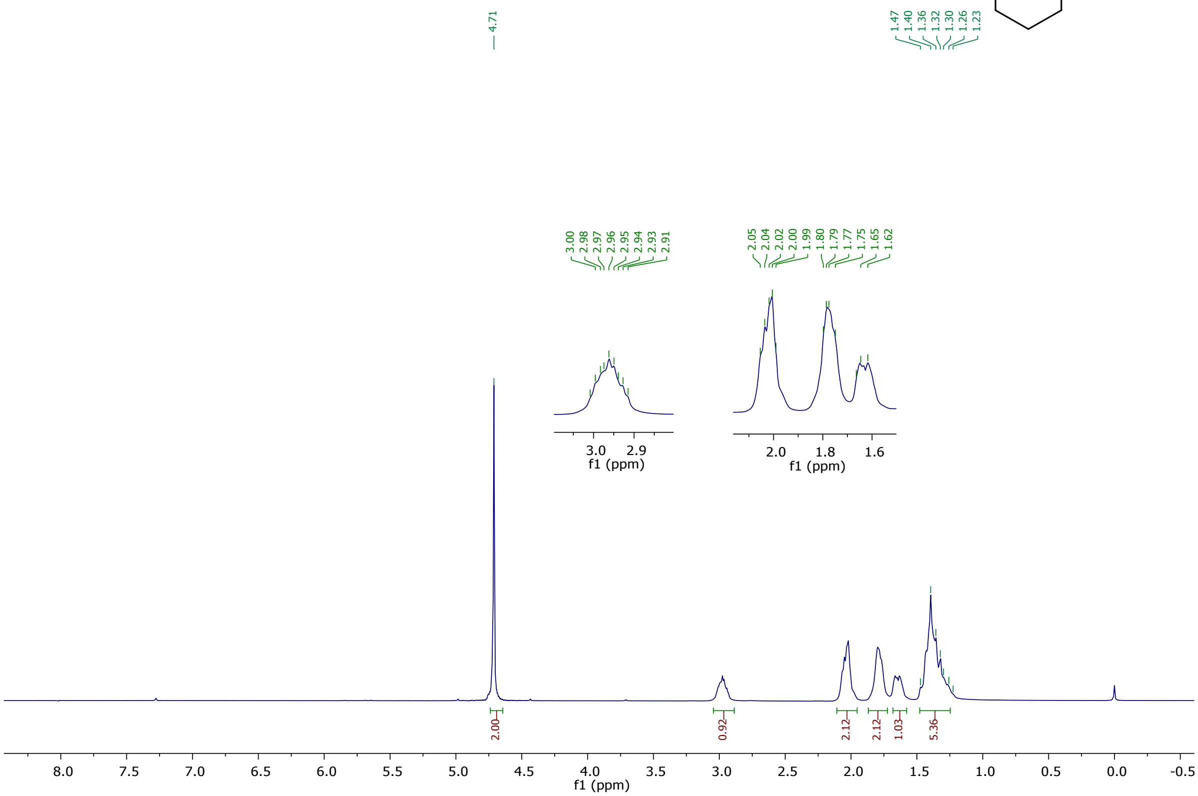

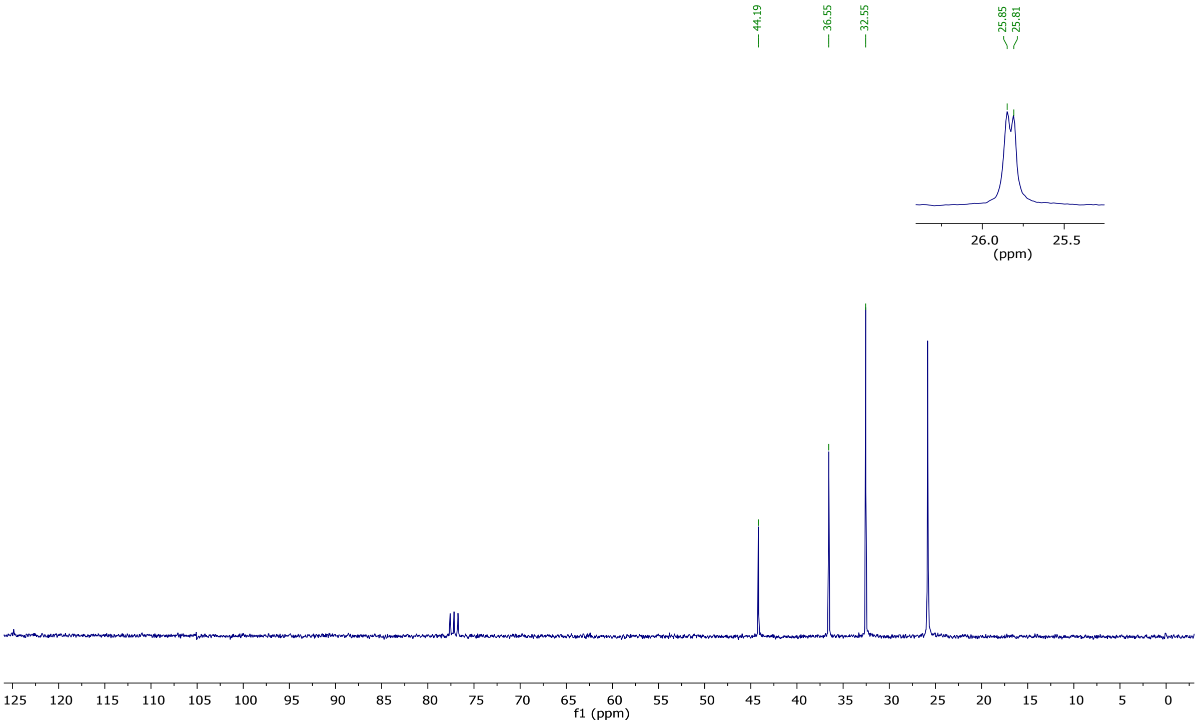

(Bromomethyl)(isopropyl)sulfane (2g)

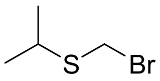

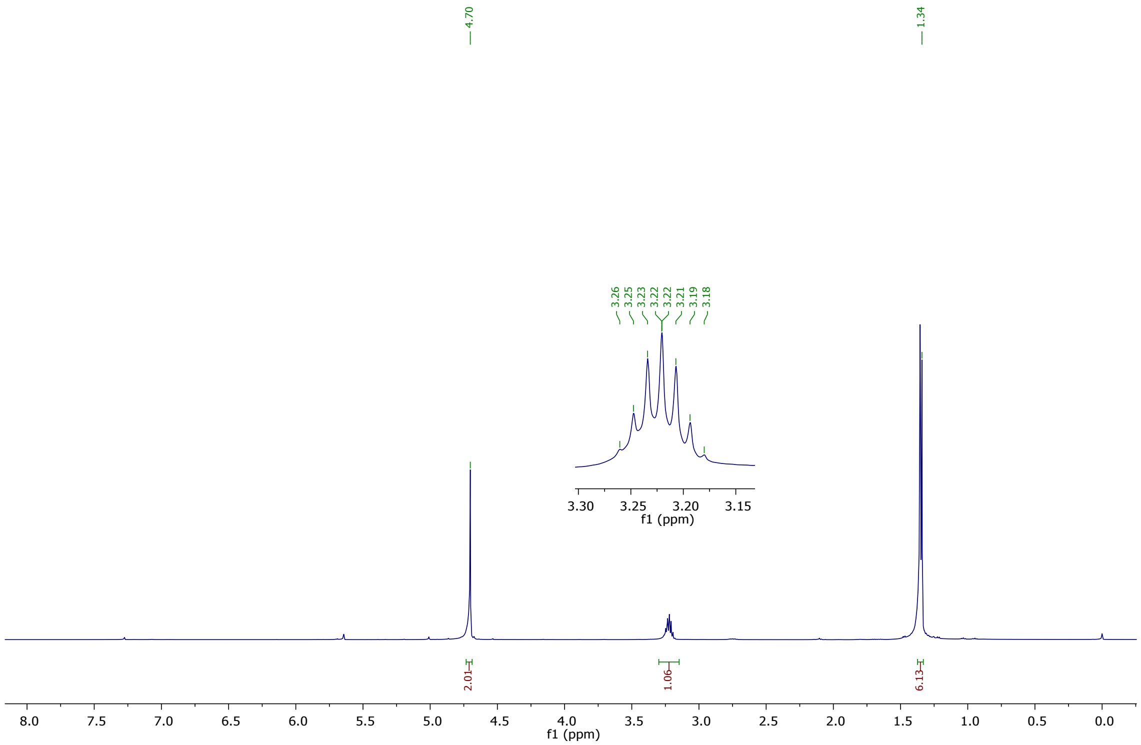

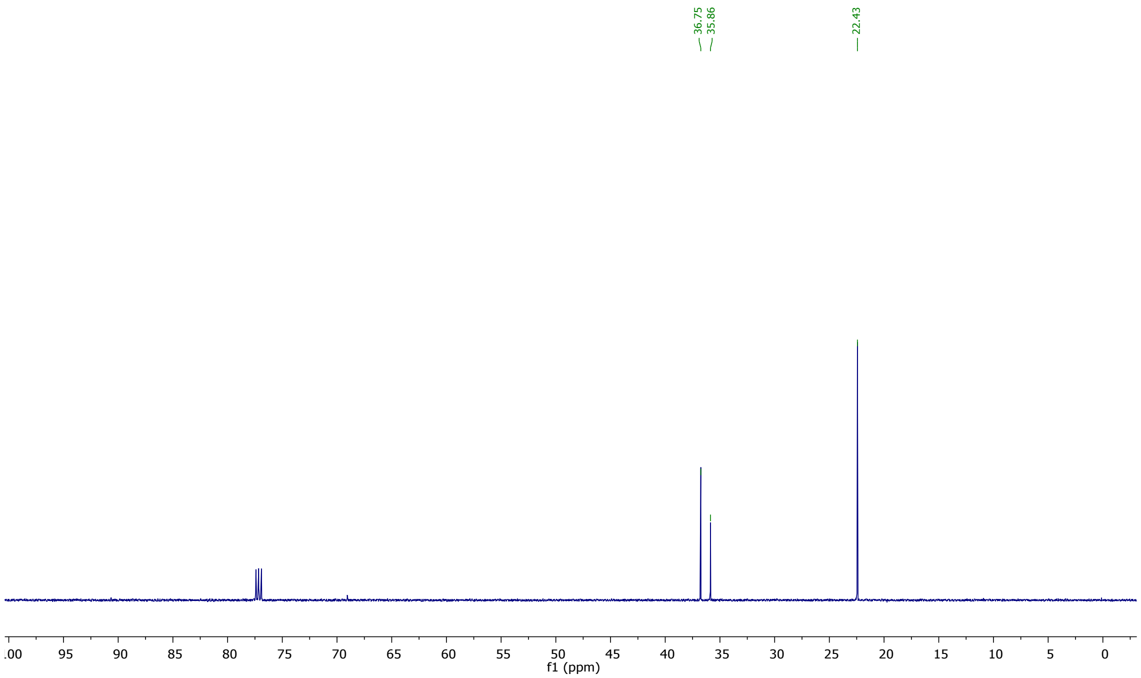

(Bromomethyl)((*1R, 2S, 5R*)-2-isopropyl-5-methylcyclohexyl)sulfane (2h)

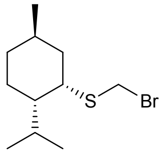

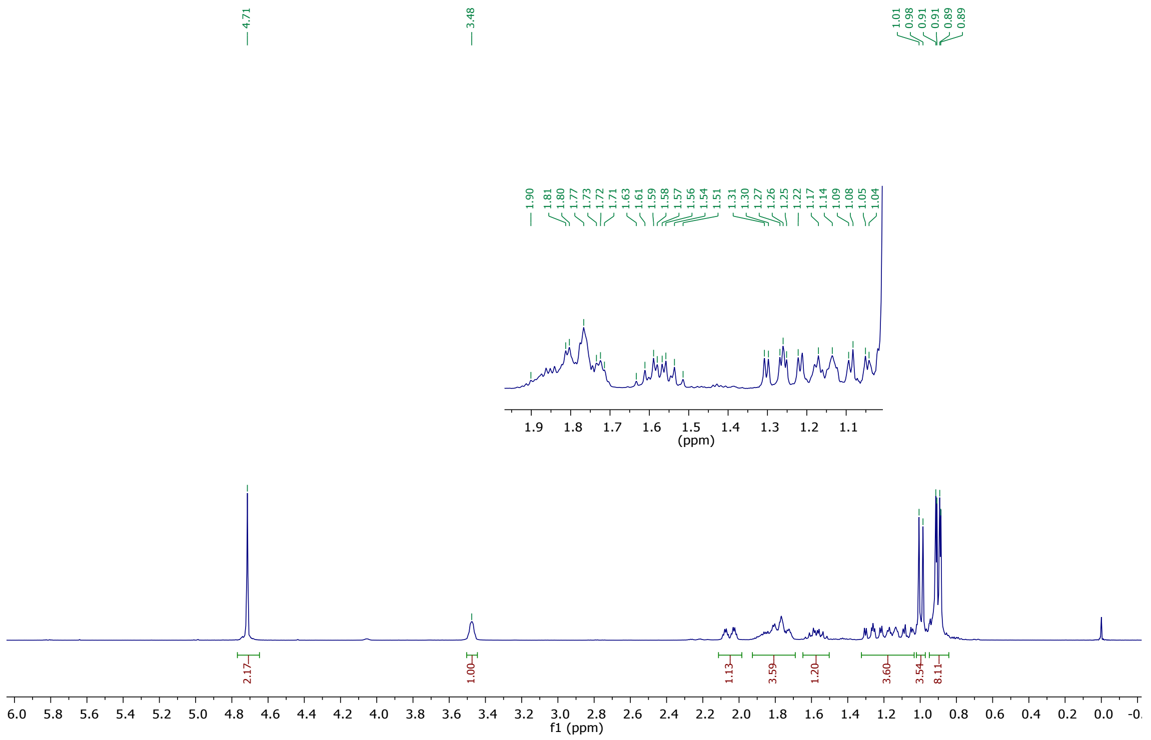

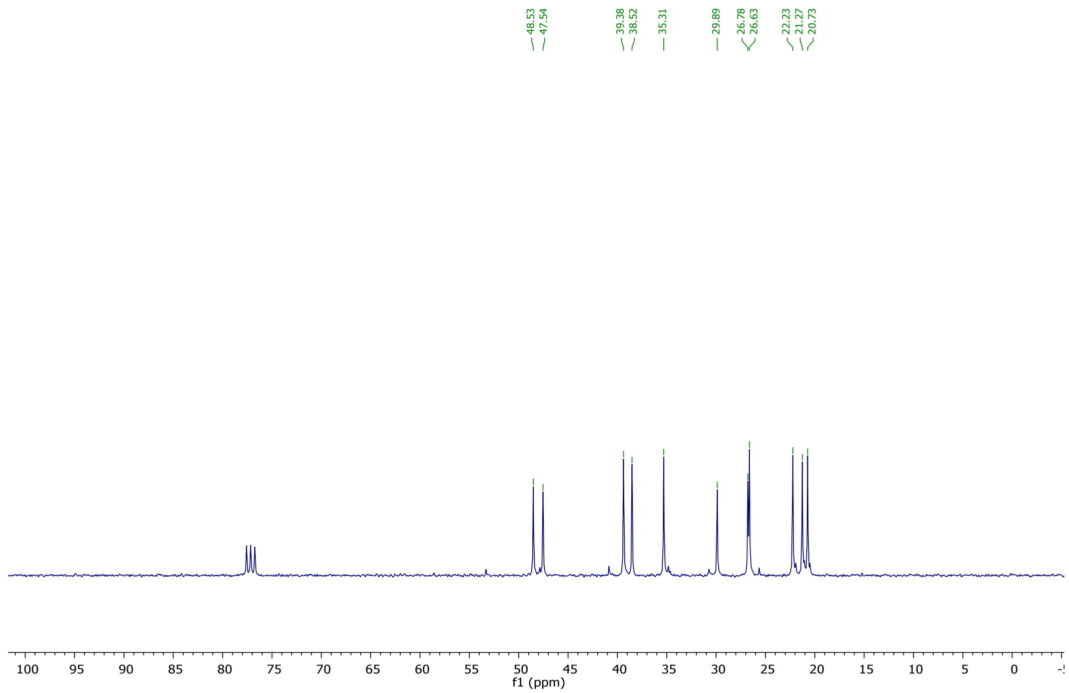

(Bromomethyl)(tert-butyl)sulfane (2i)

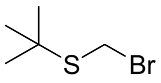

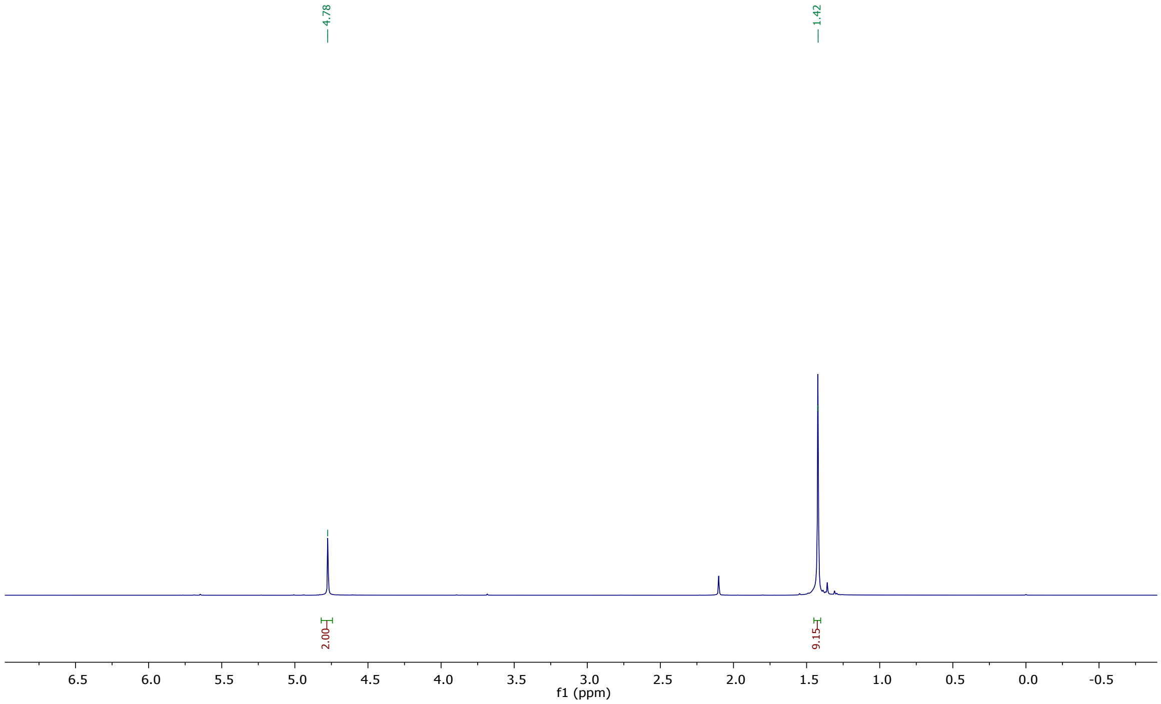

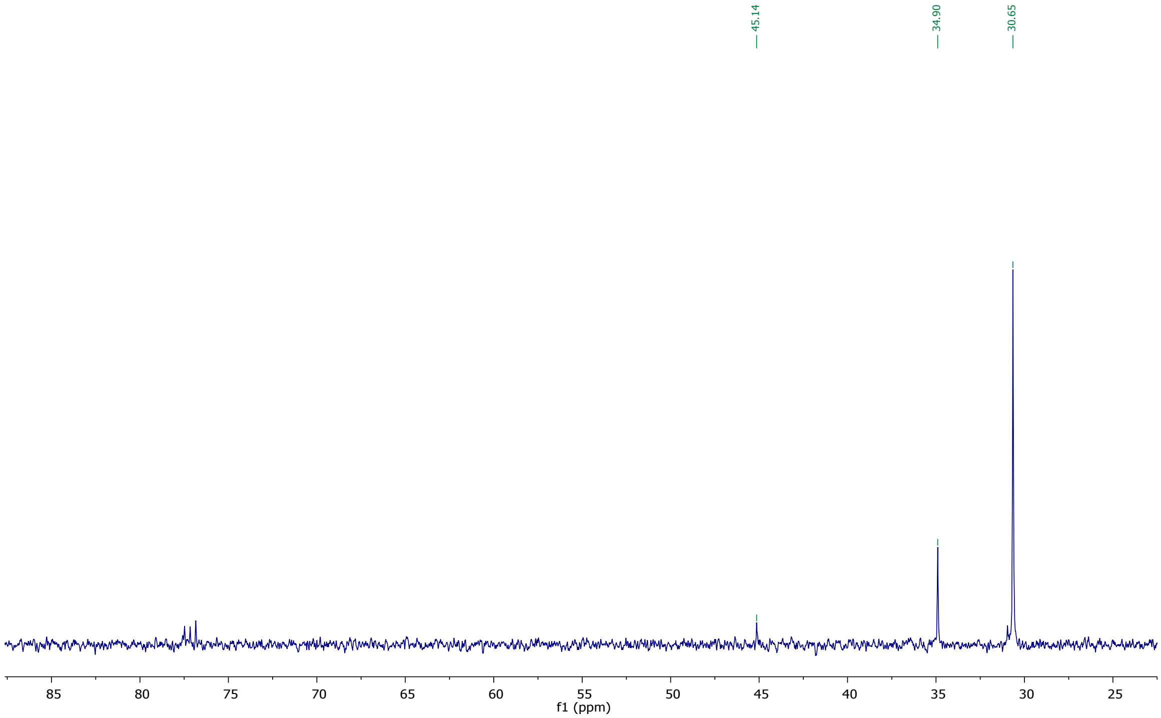

(Bromomethyl)(phenyl)(sulfane) (2j)

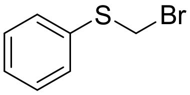

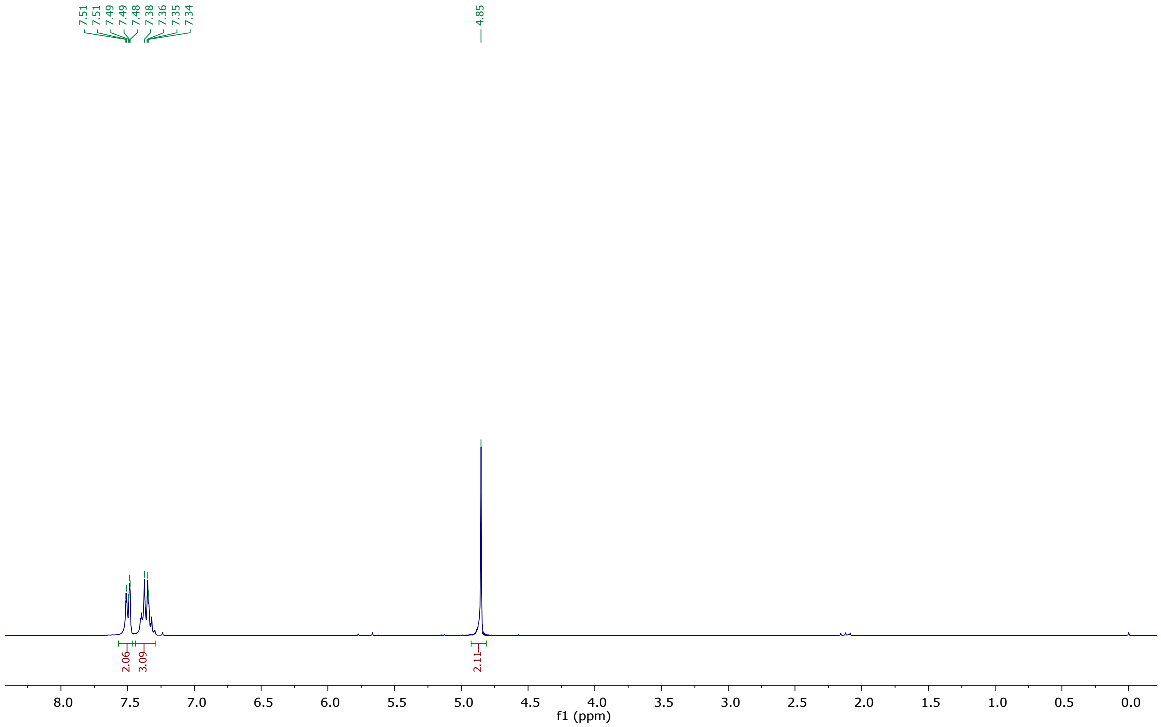

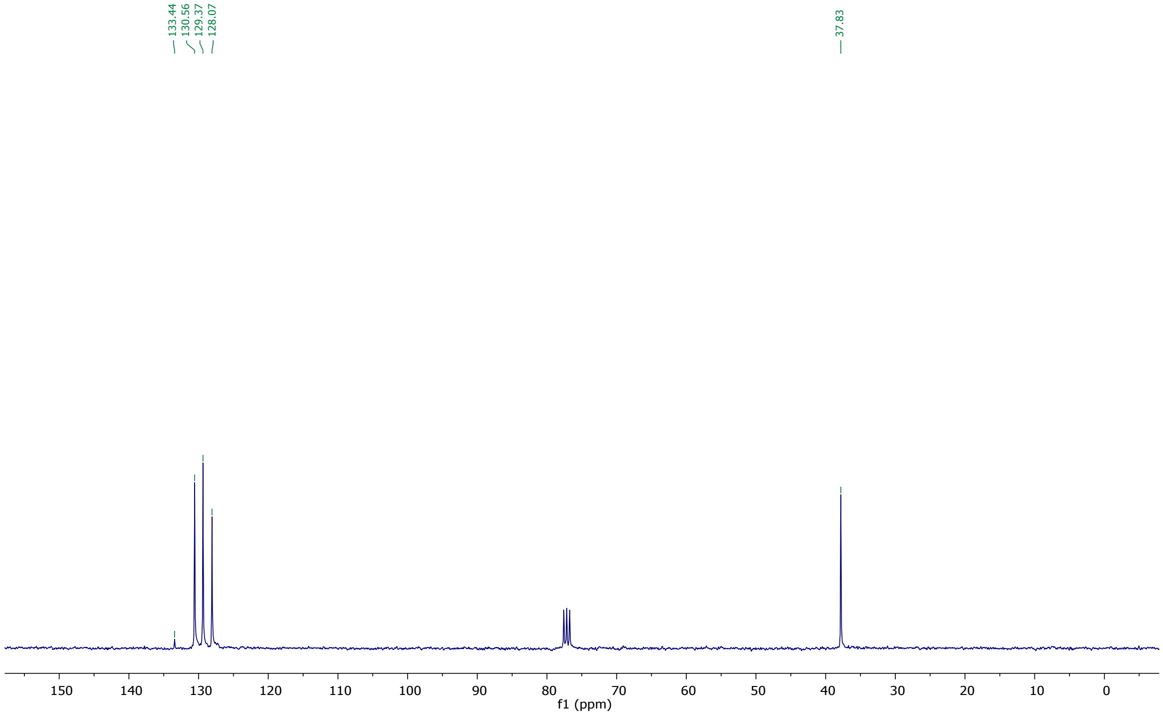

1-[(bromomethyl)thio]-4-methylbenzene (2k)

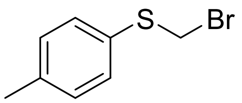

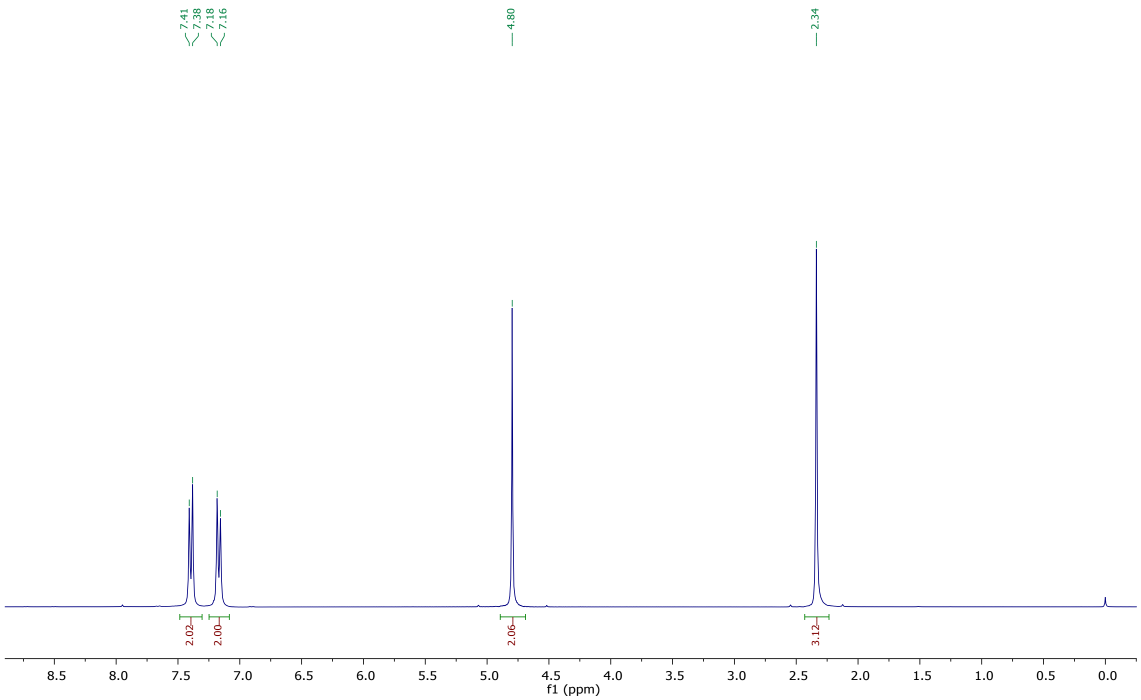

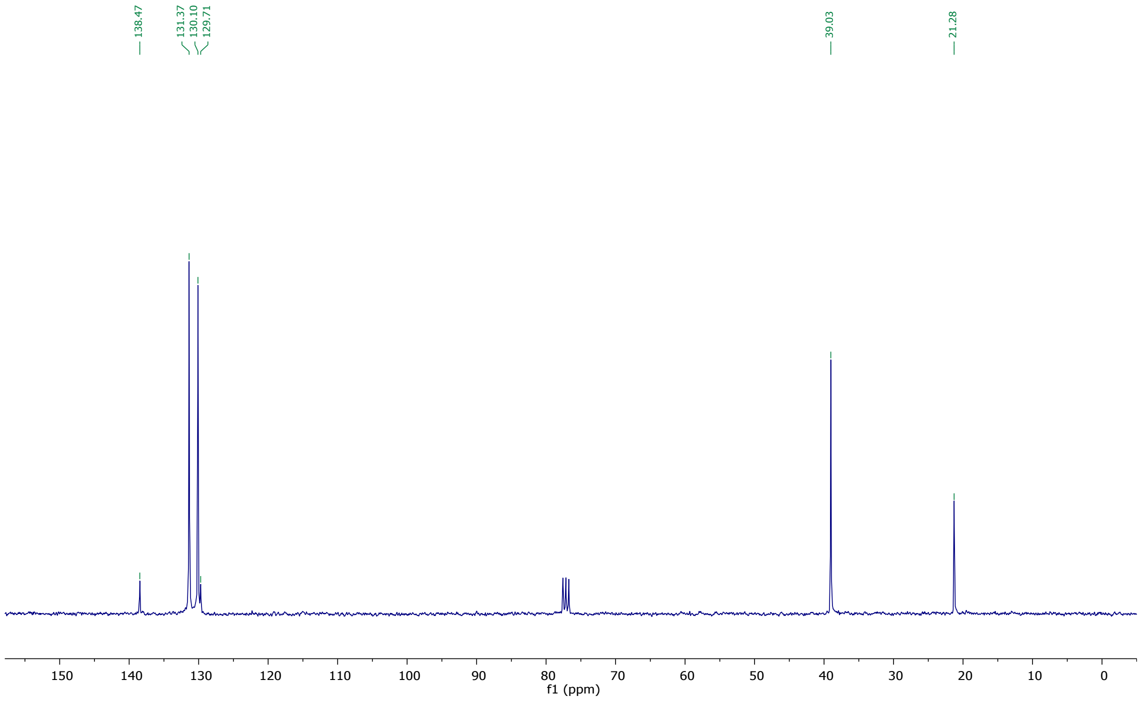

(Bromomethyl)(*o*-tolyl)sulfane (2l)

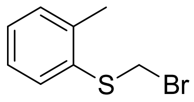

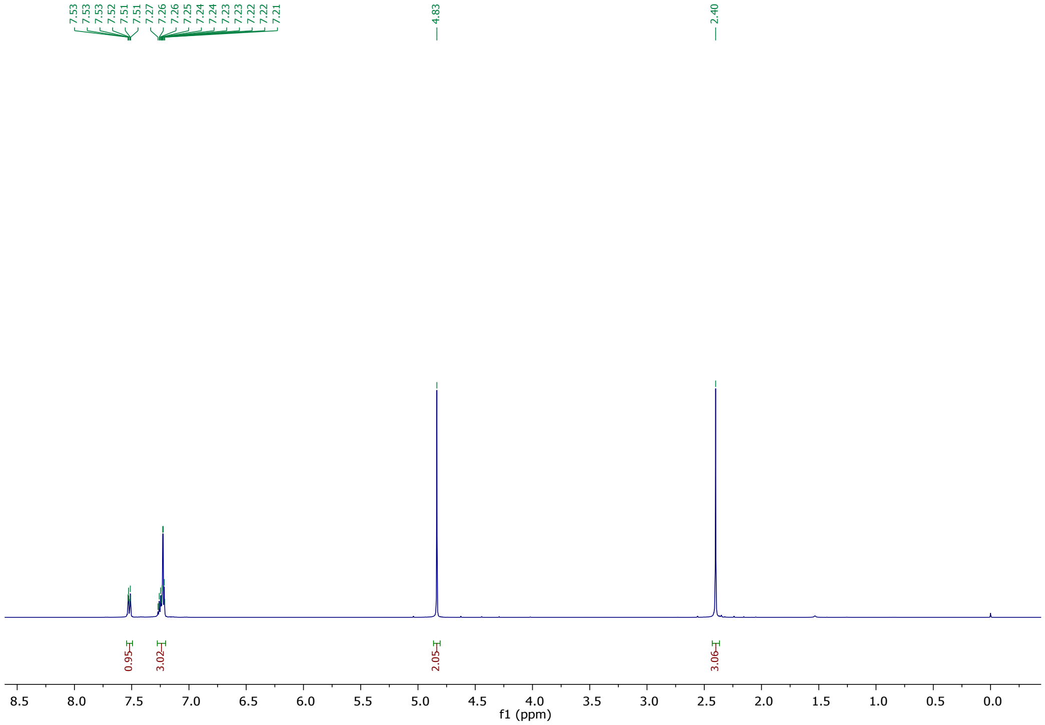

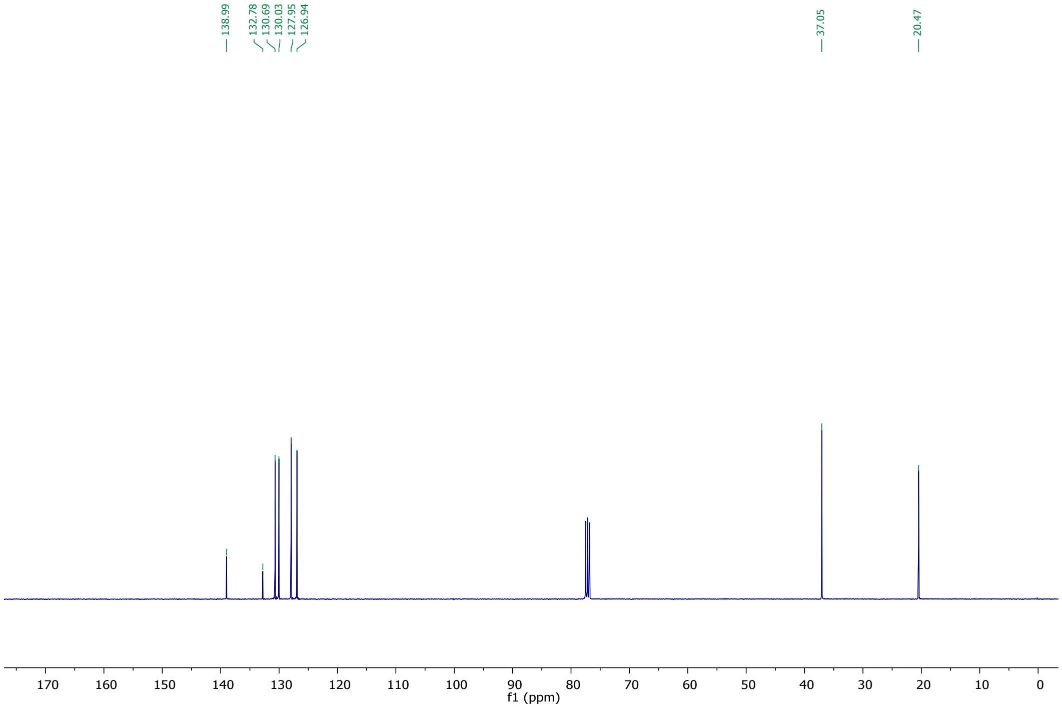

(Bromomethyl)(4-chlorophenyl)sulfane (2m)

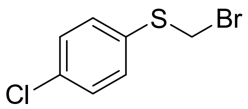

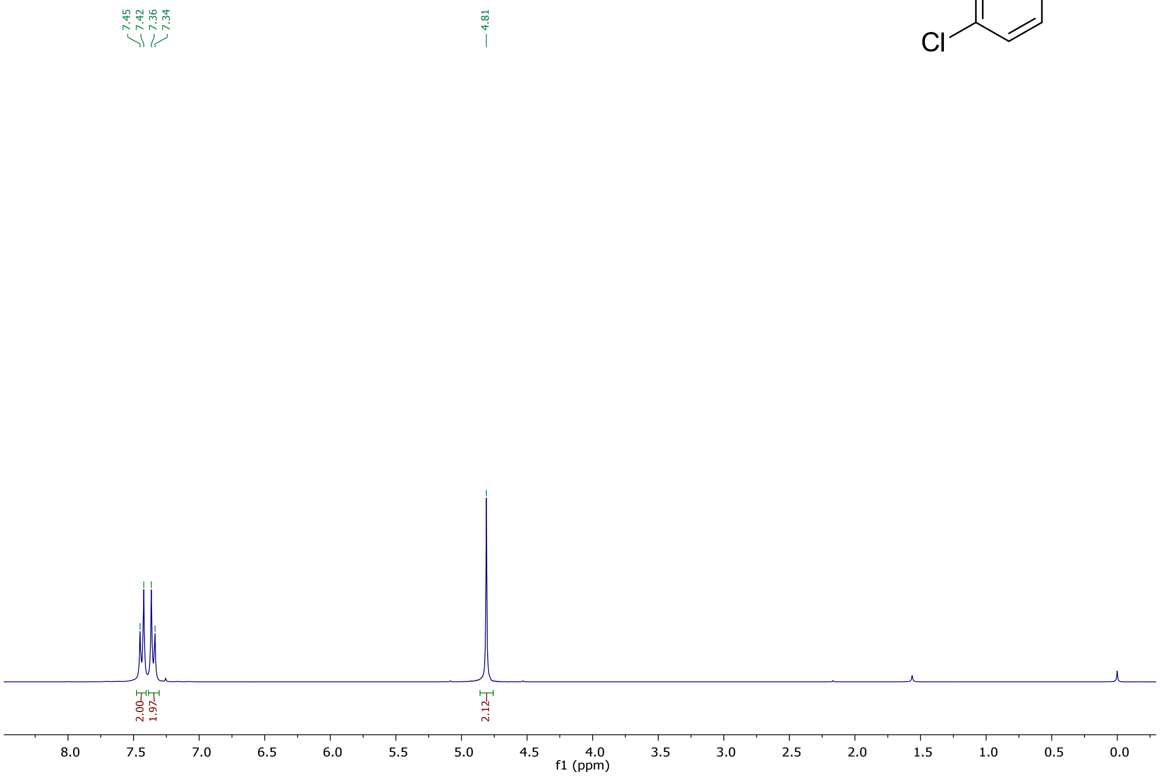

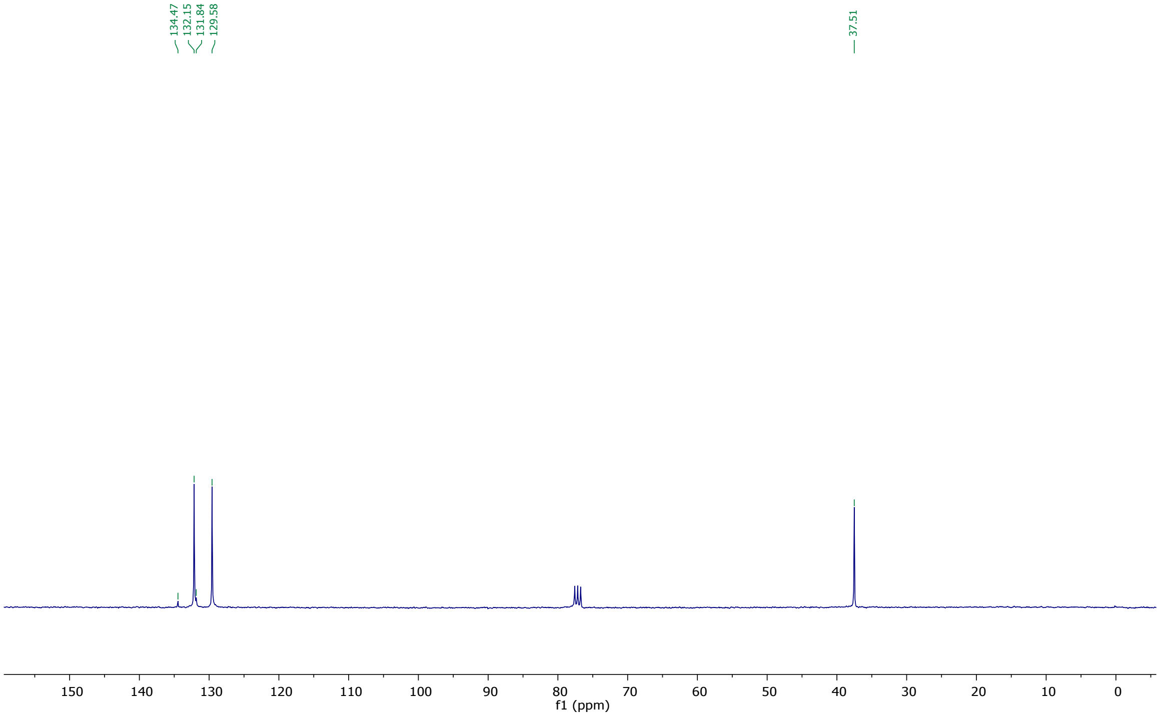

(Bromomethyl)(3-chlorophenyl)sulfane (2n)

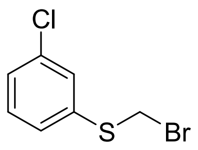

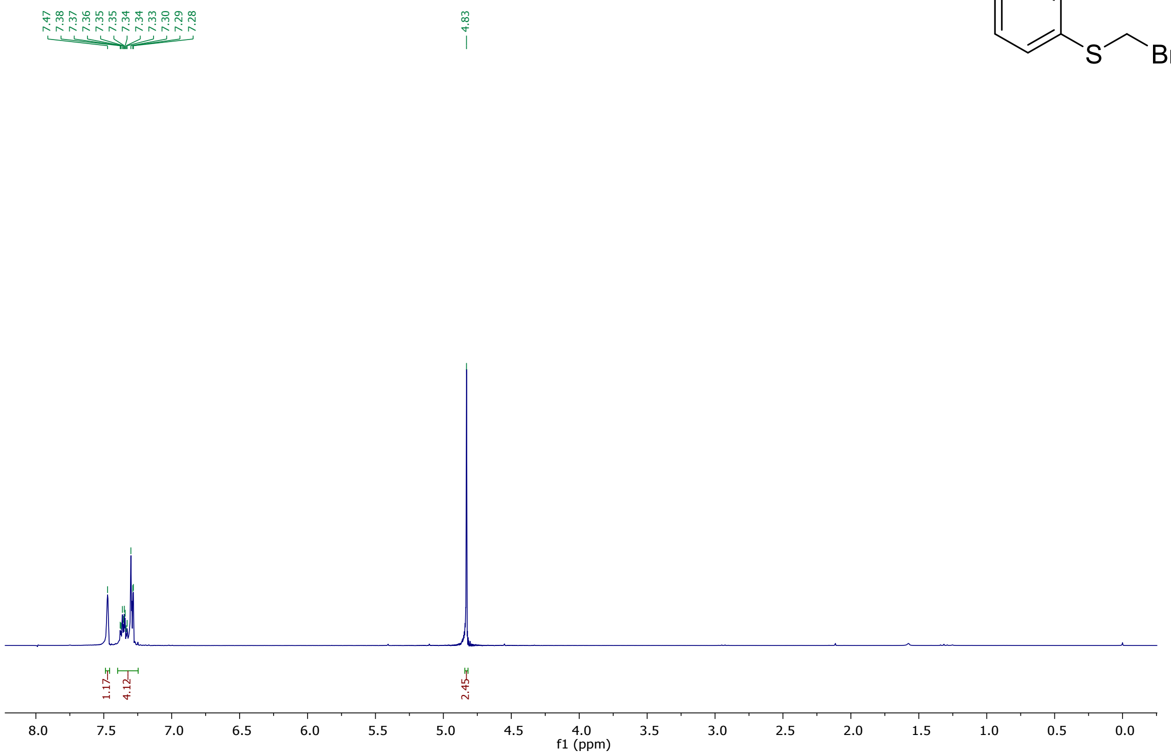

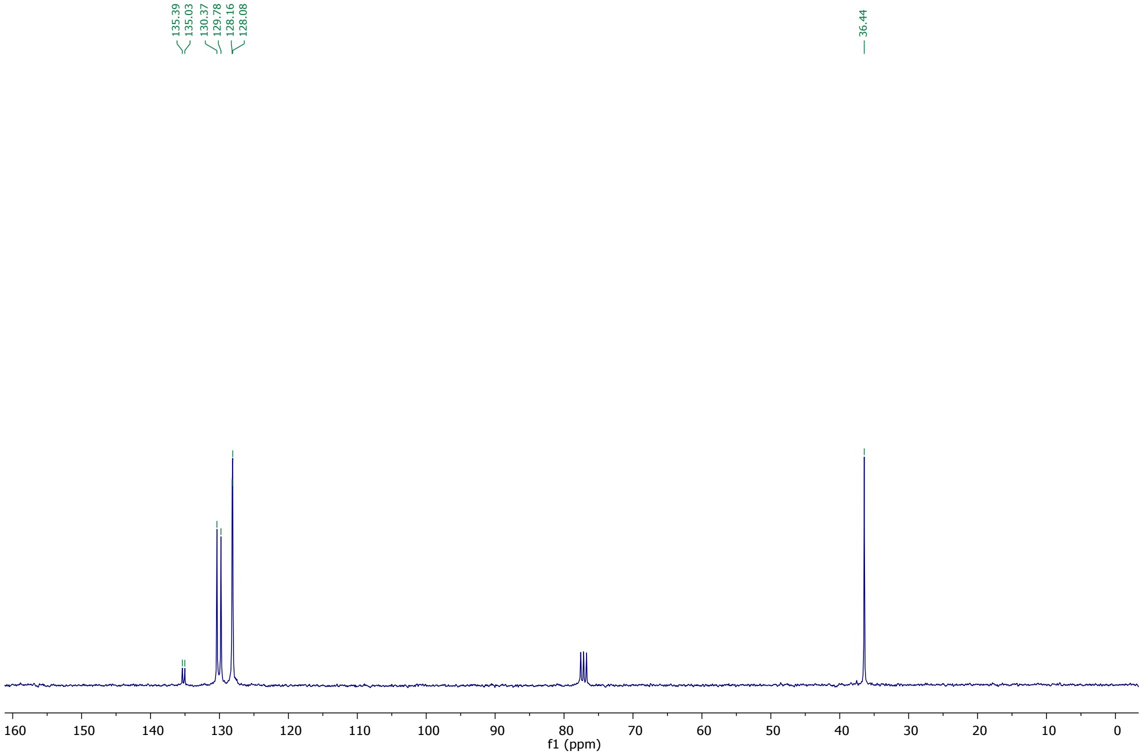

(Bromomethyl)(4-bromophenyl)sulfane (2o)

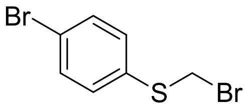

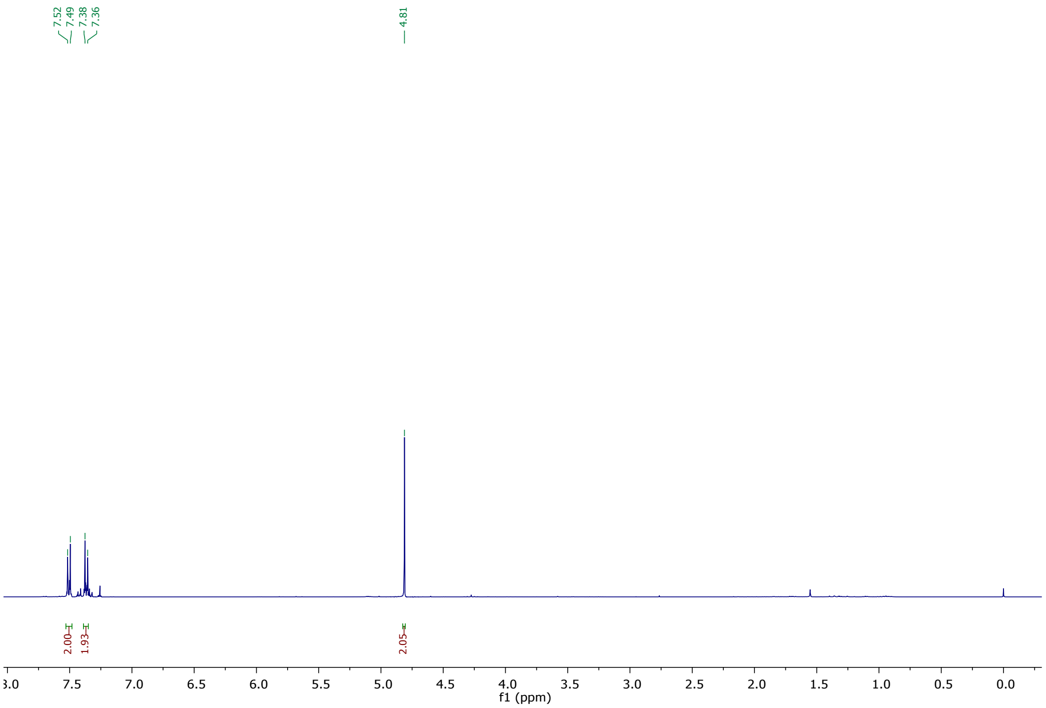

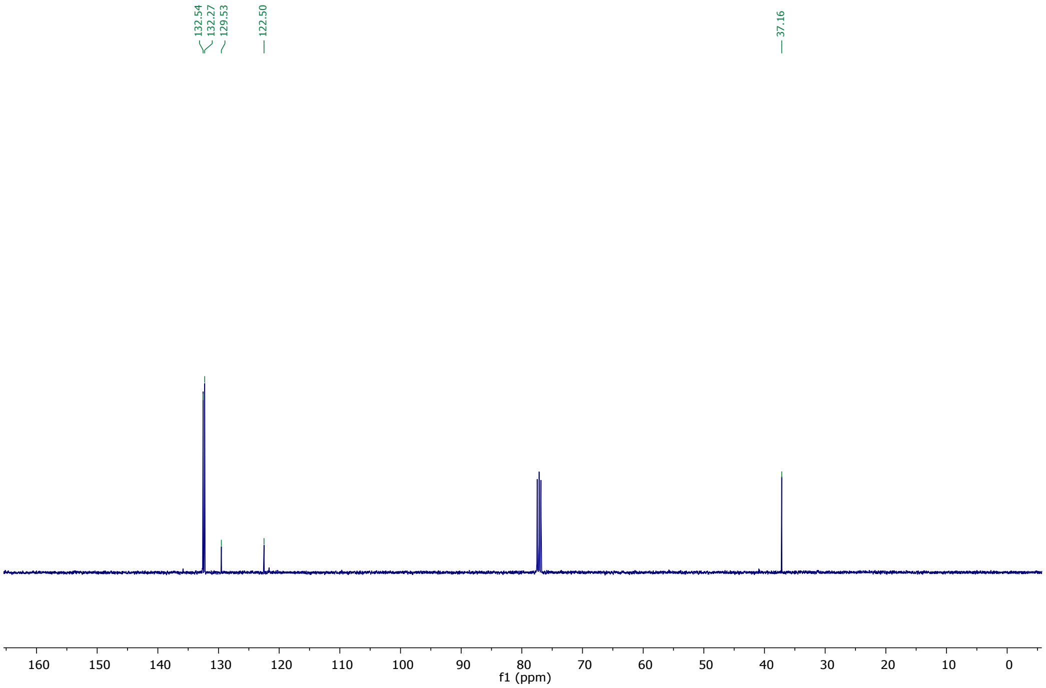

(Bromomethyl)(4-fluorophenyl)sulfane (2p)

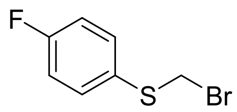

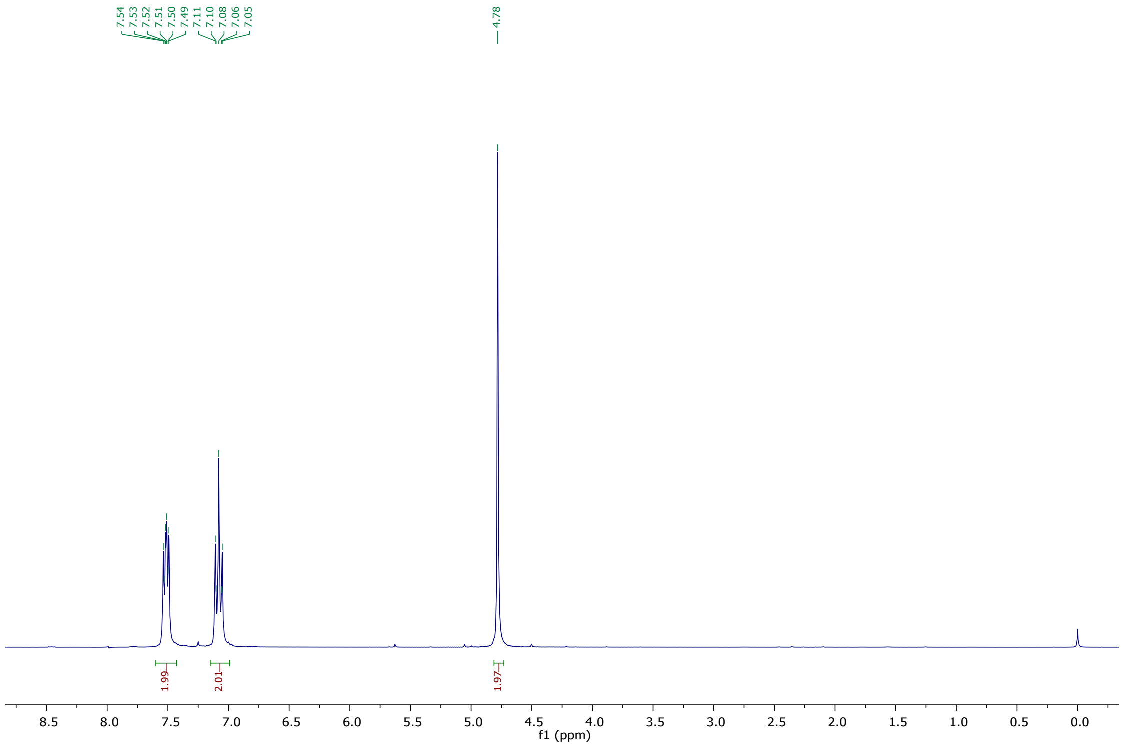

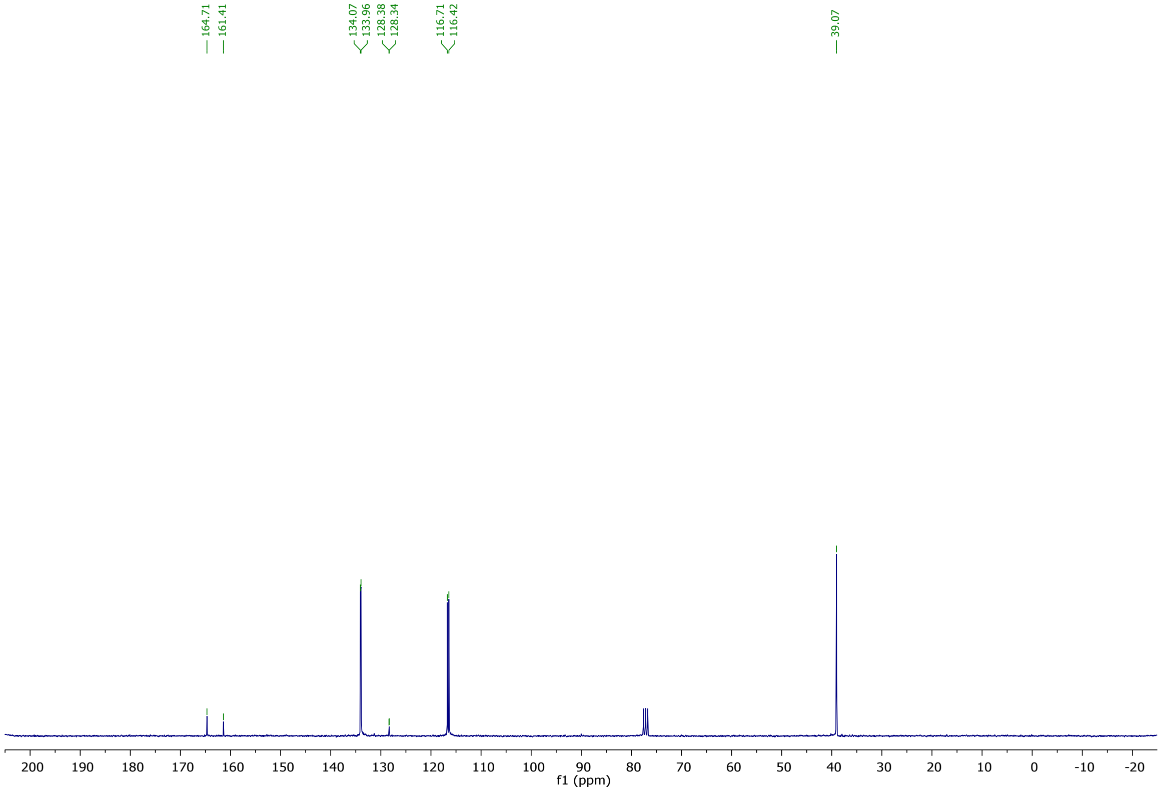

(Bromomethyl)(4-methoxyphenyl)sulfane (2q)

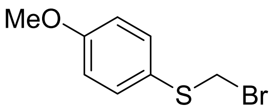

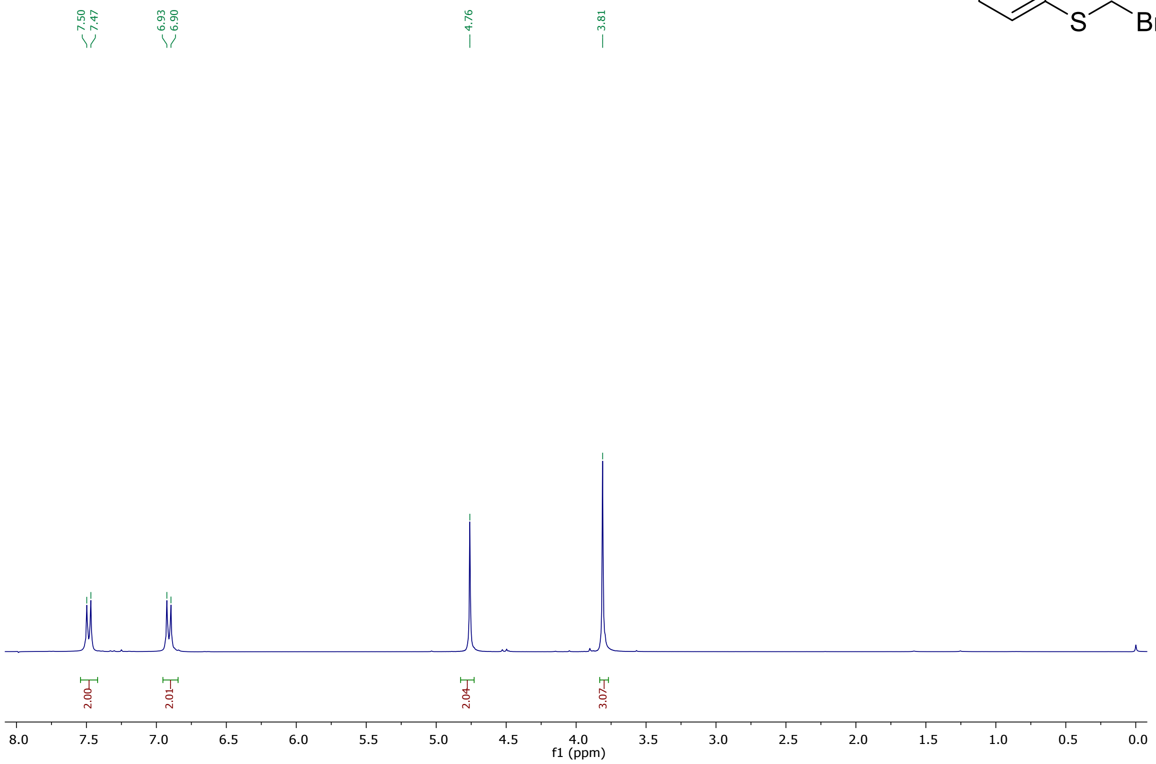

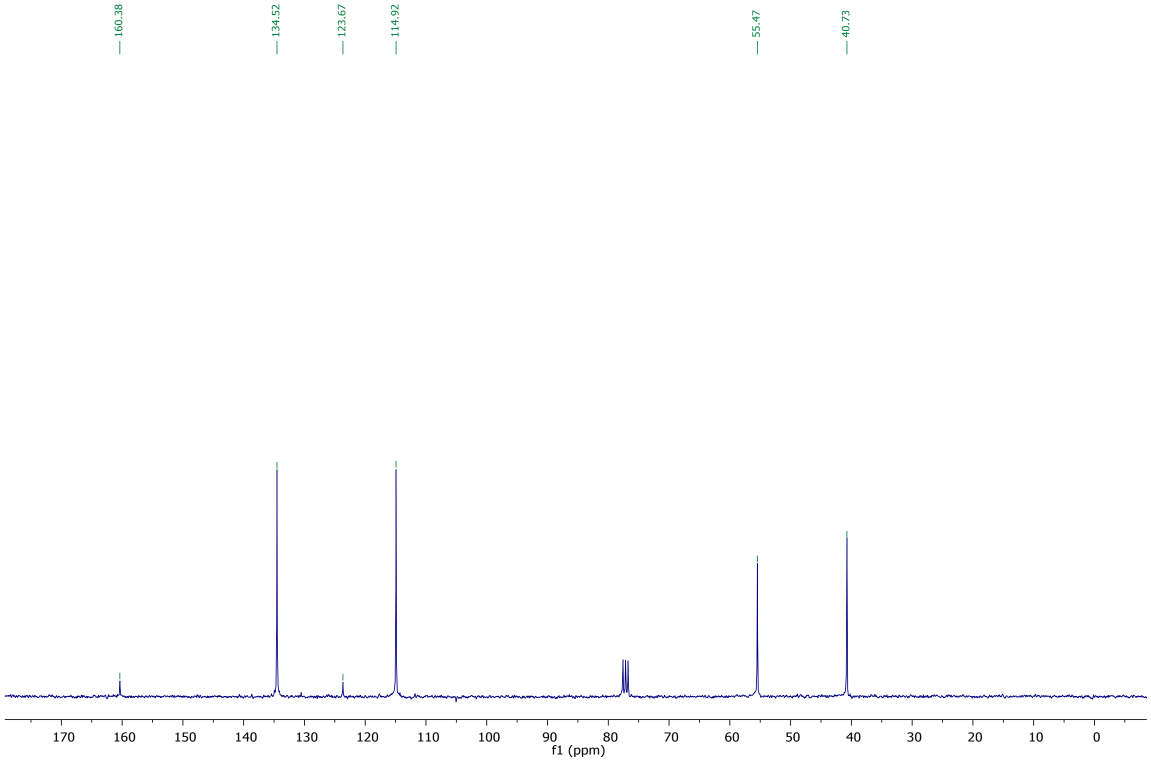

(Bromomethyl)(2-methoxyphenyl)sulfane (2r)

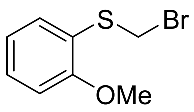

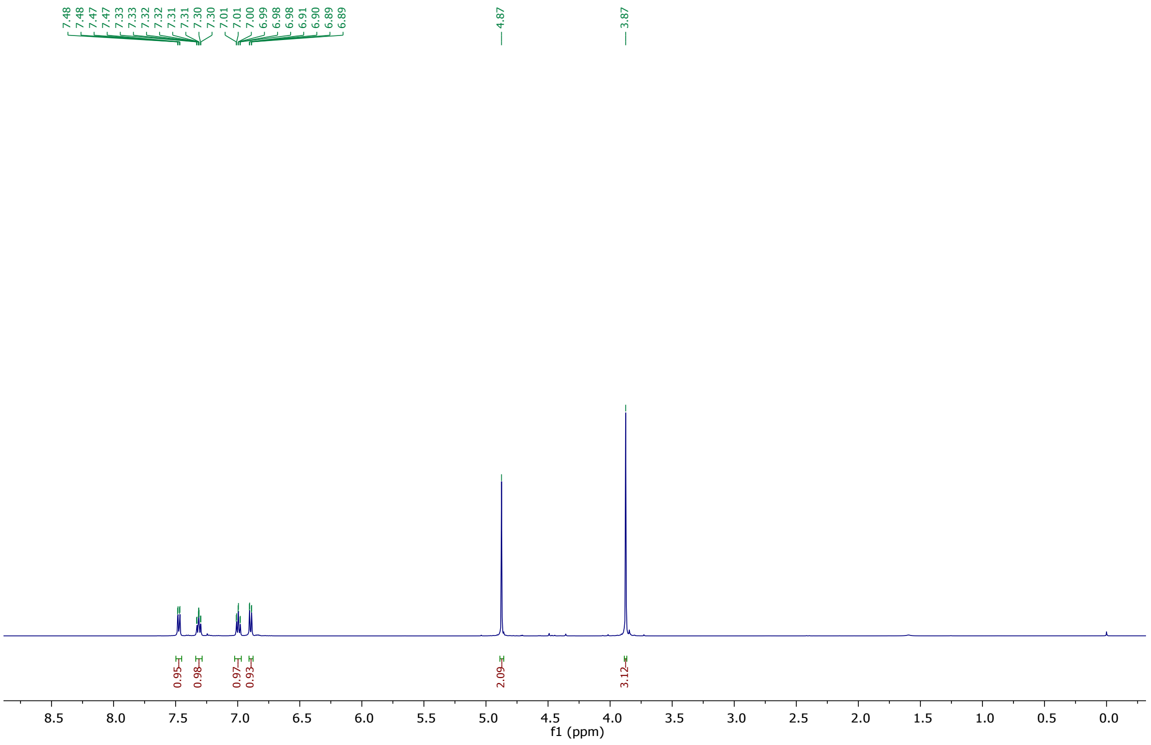

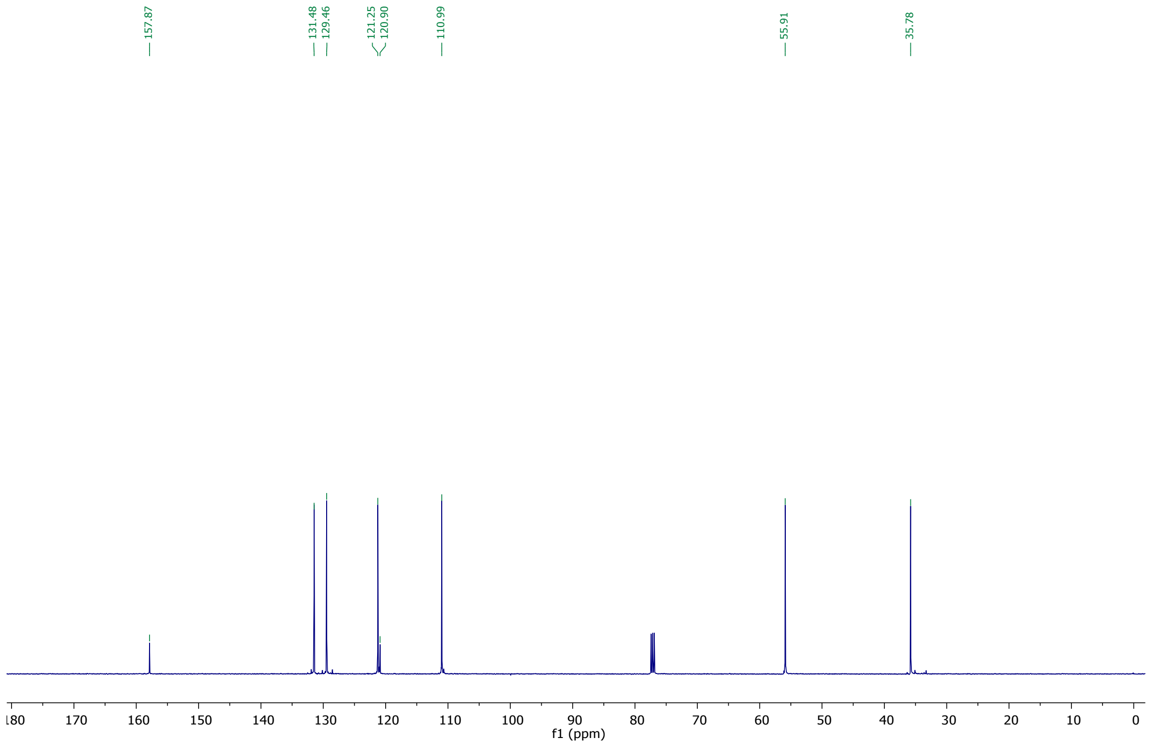

4-((bromomethyl)thio)benzonitrile (2s)

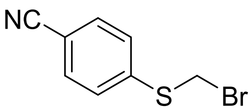

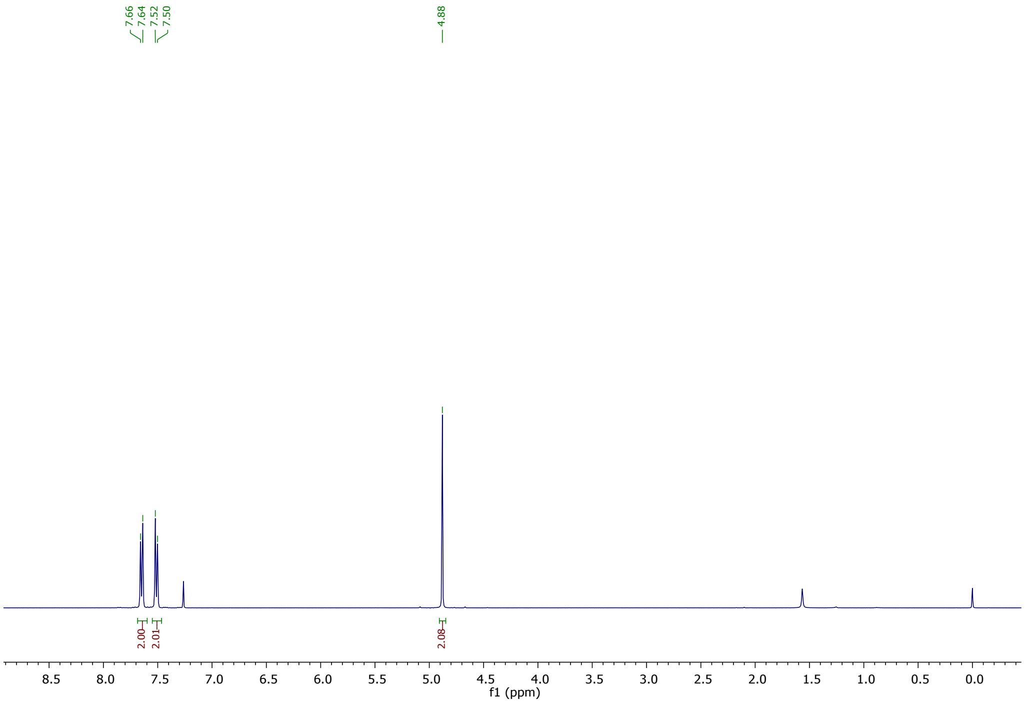

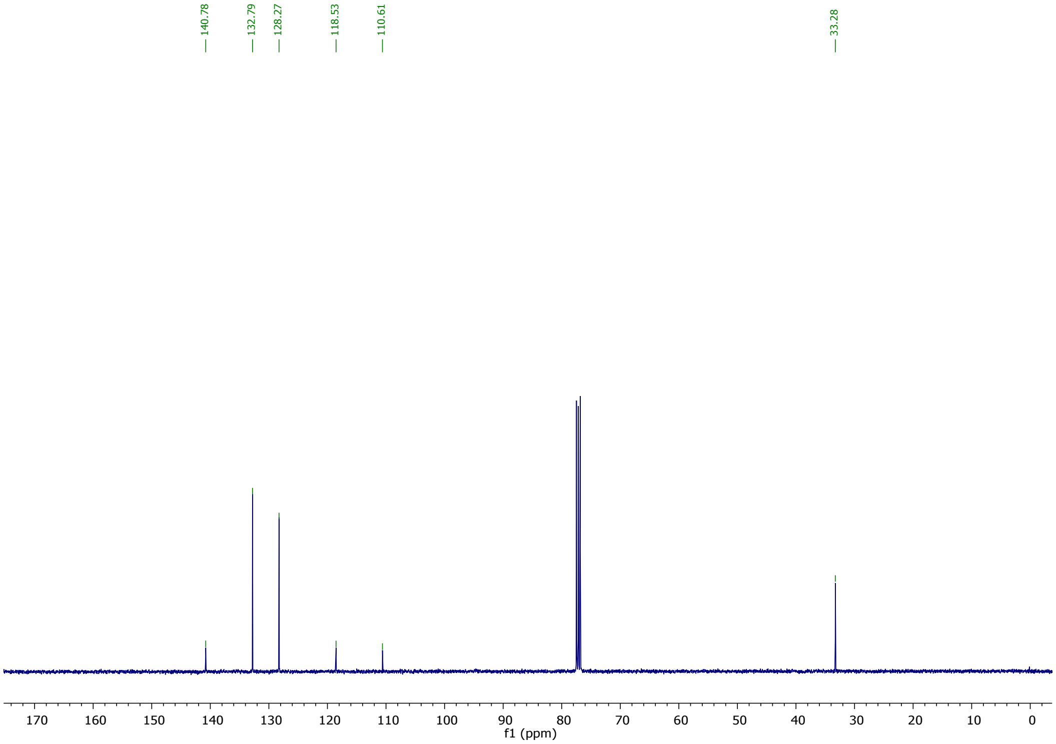

2-((Bromomethyl)thio)benzaldehyde (2t)

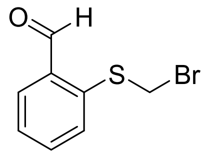

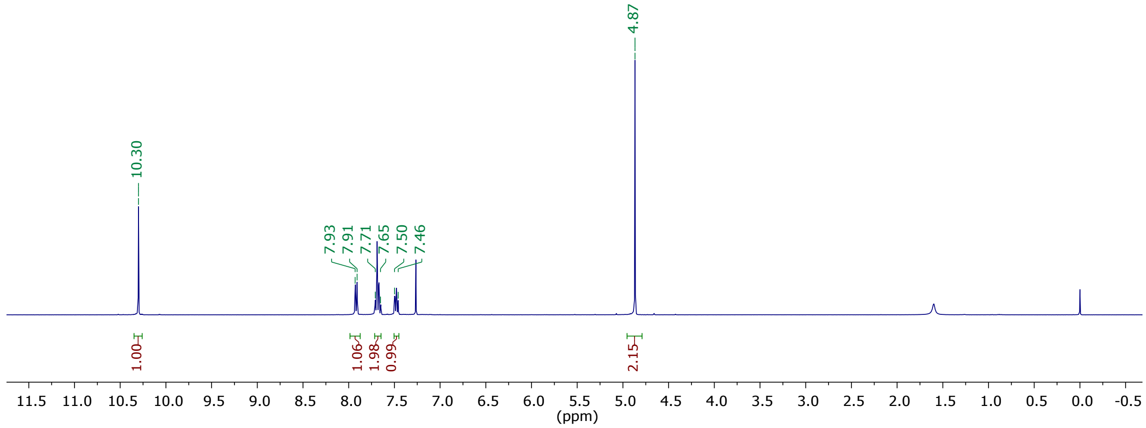

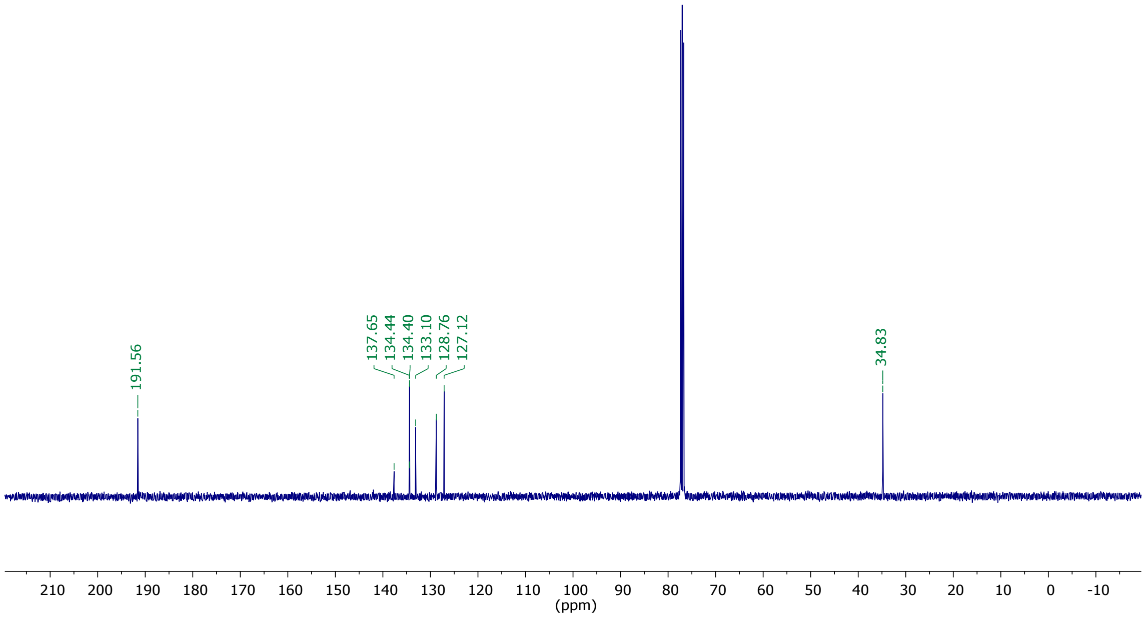

1-(2-((bromomethyl)thio)phenyl)ethan-1-one (2u)

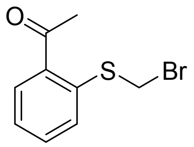

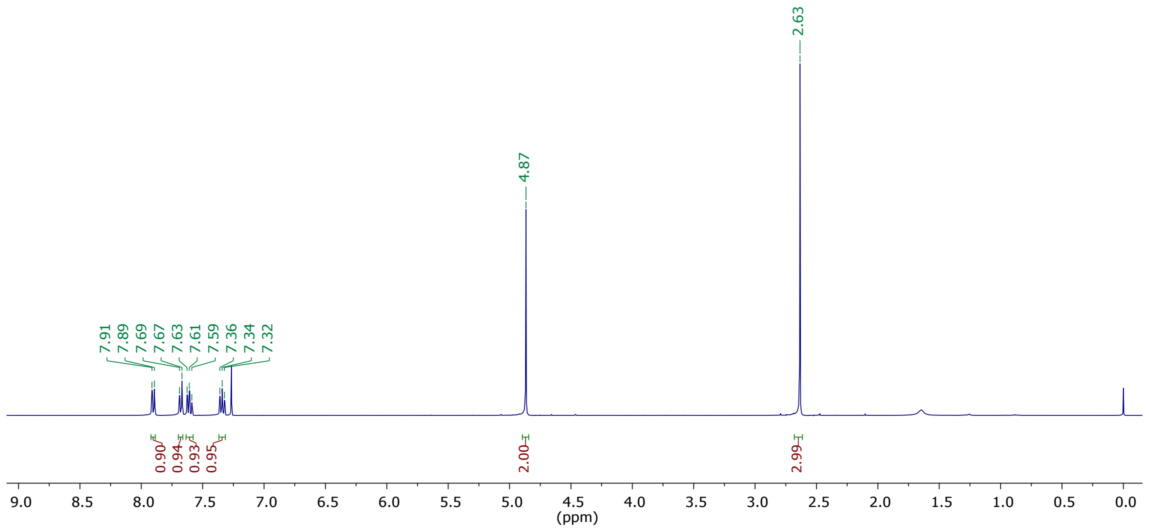

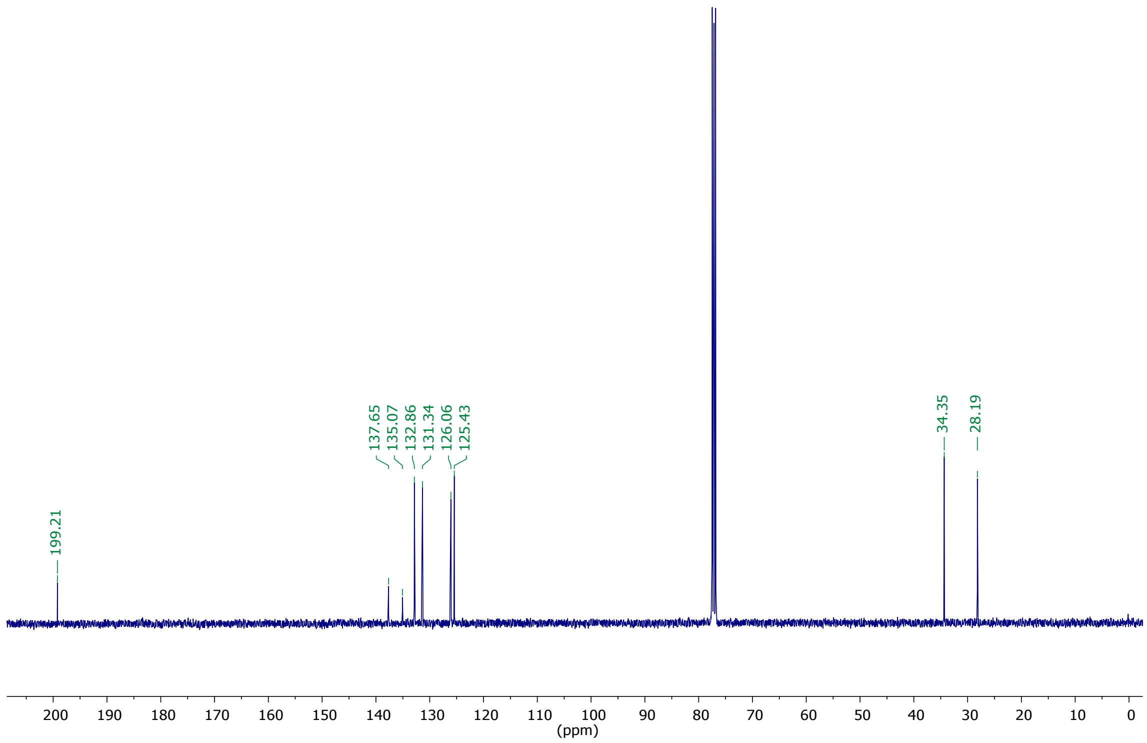

Methyl 2-((bromomethyl)thio)benzoate (2v)

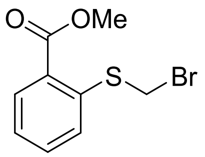

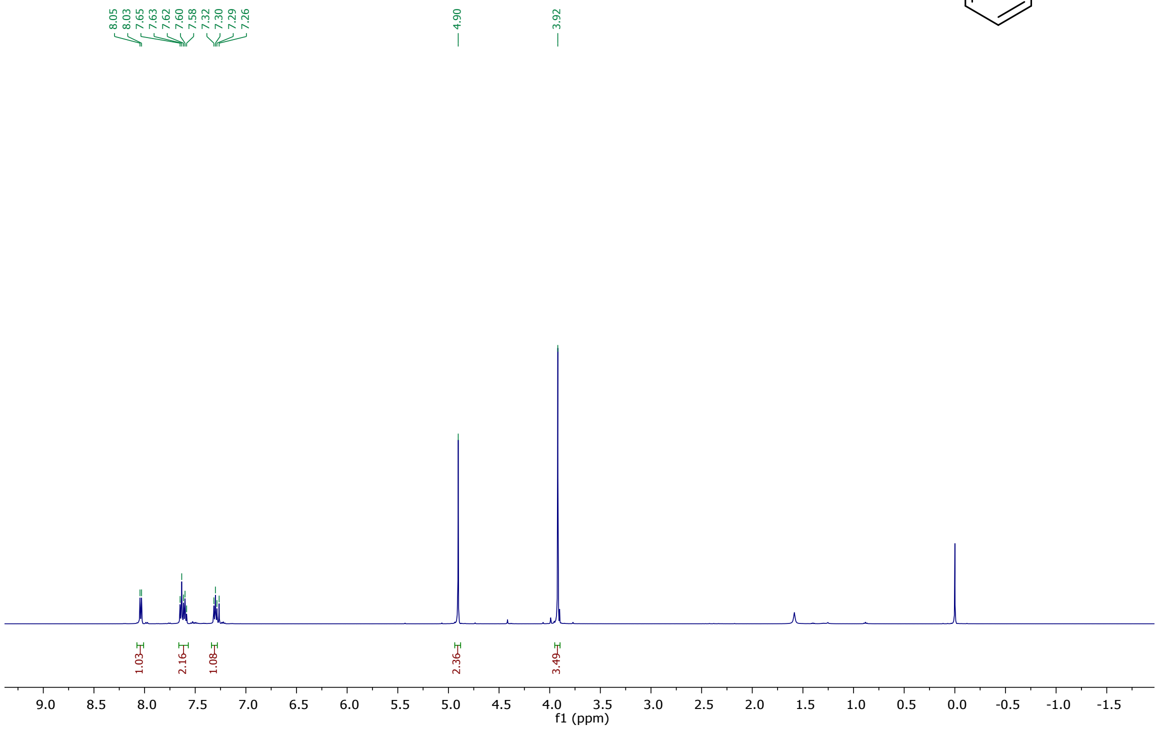

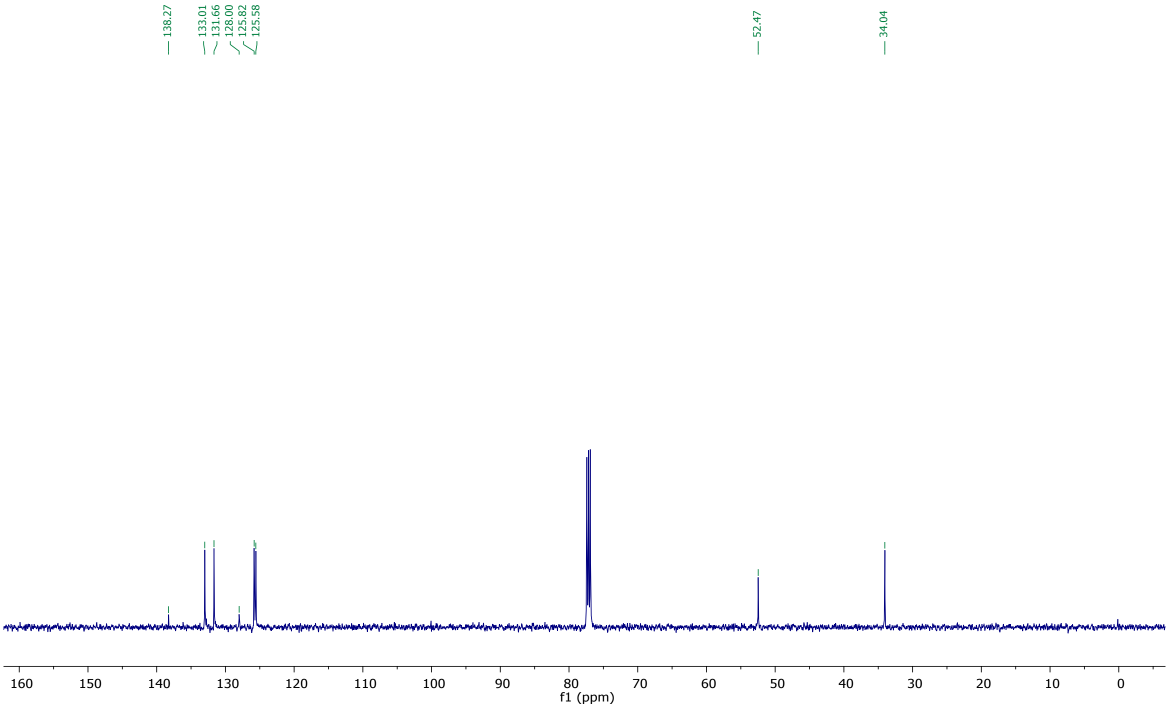

(Bromo(4-nitrophenyl)methyl)(*p*-tolyl)sulfane (2k1)

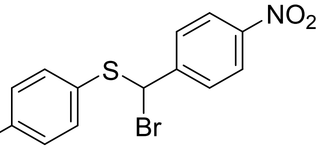

A- 2k1
B- 3k2
C-4-Nitrobenzaldehyde

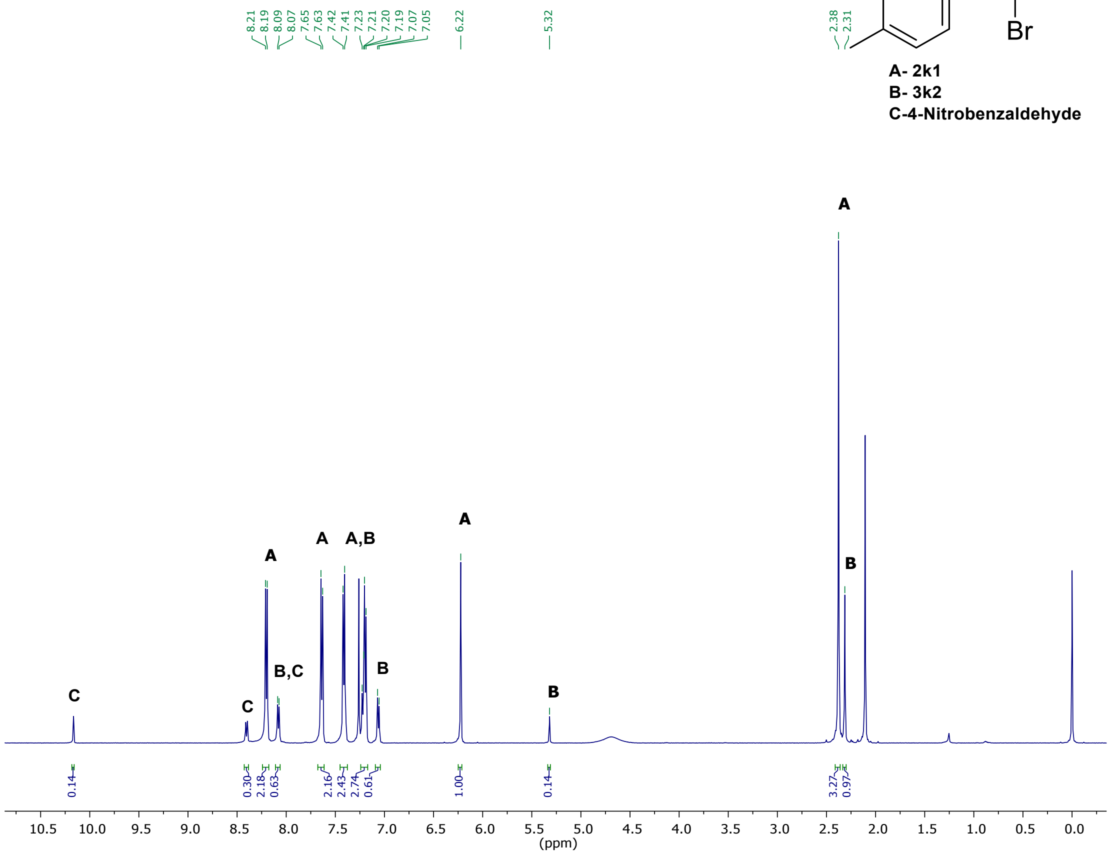

A- 2k1
B- 3k2
C-4-Nitrobenzaldehyde

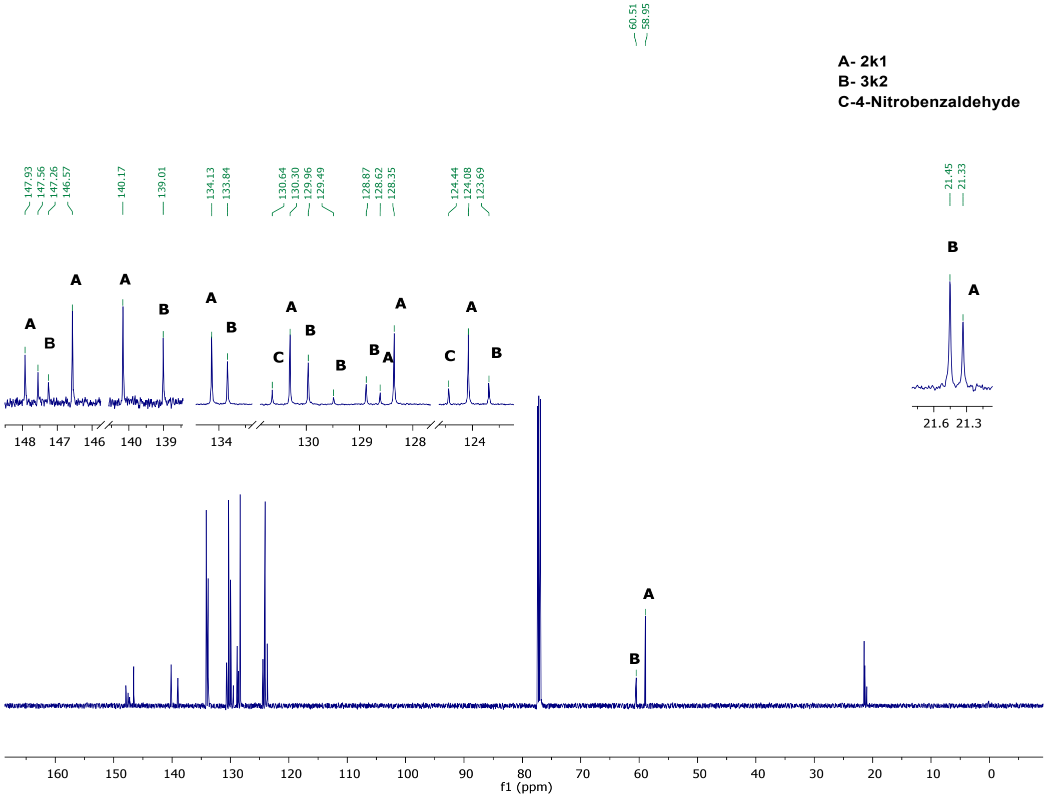

((4-Nitrophenyl)methylene)bis(p-tolylsulfane) (3k1)

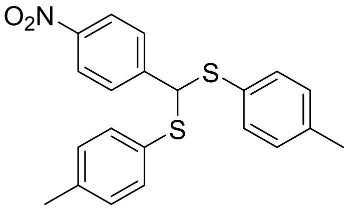

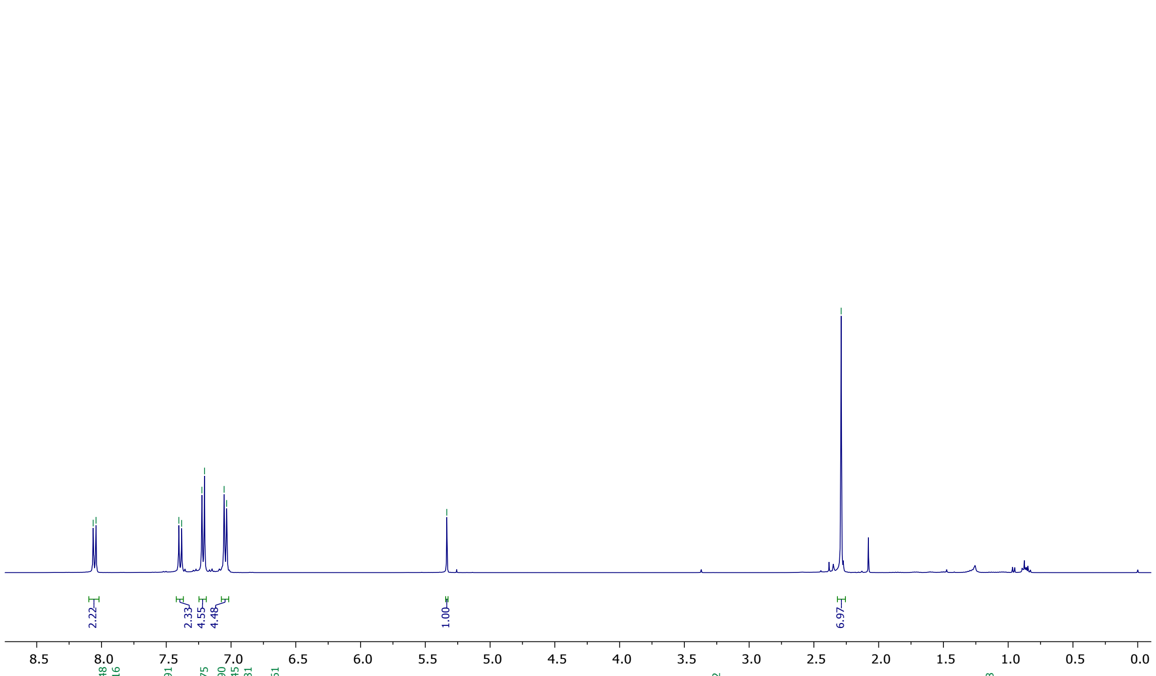

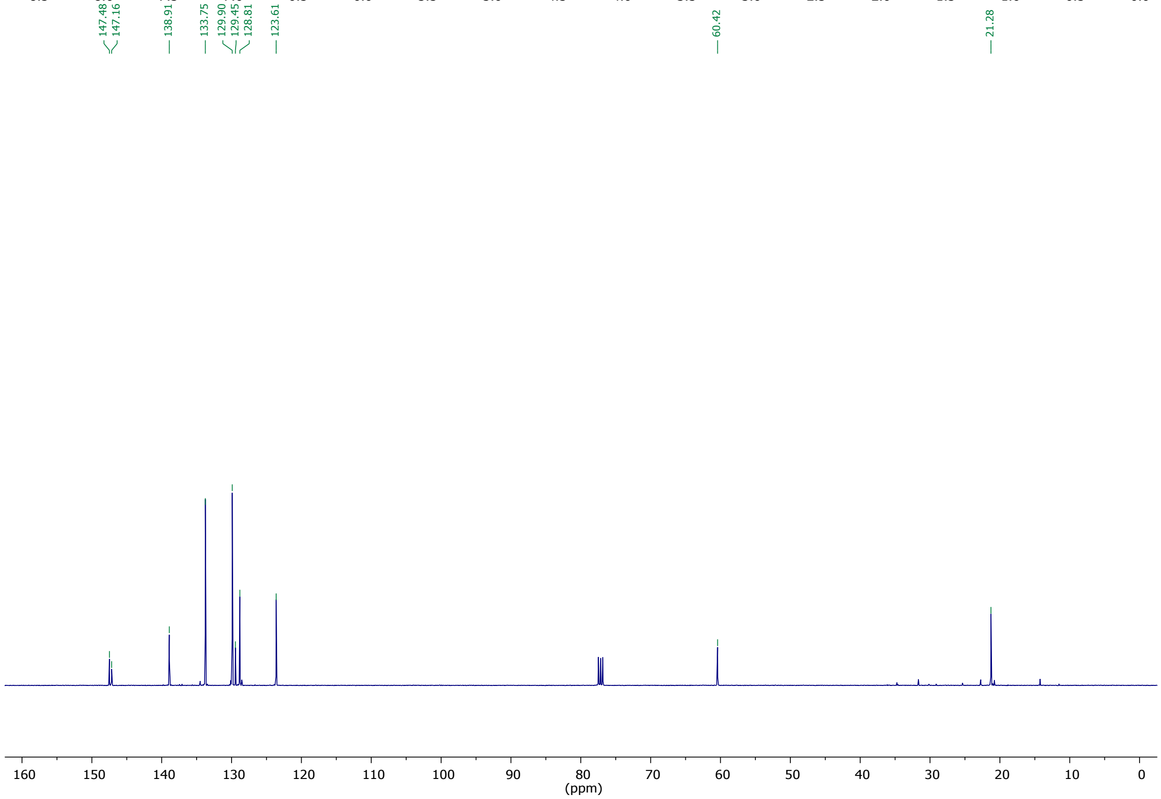

(1-Bromoethyl)(*p*-tolyl)sulfane (2k2)

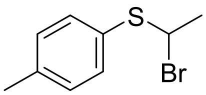

A- 2k2
B- 3k2

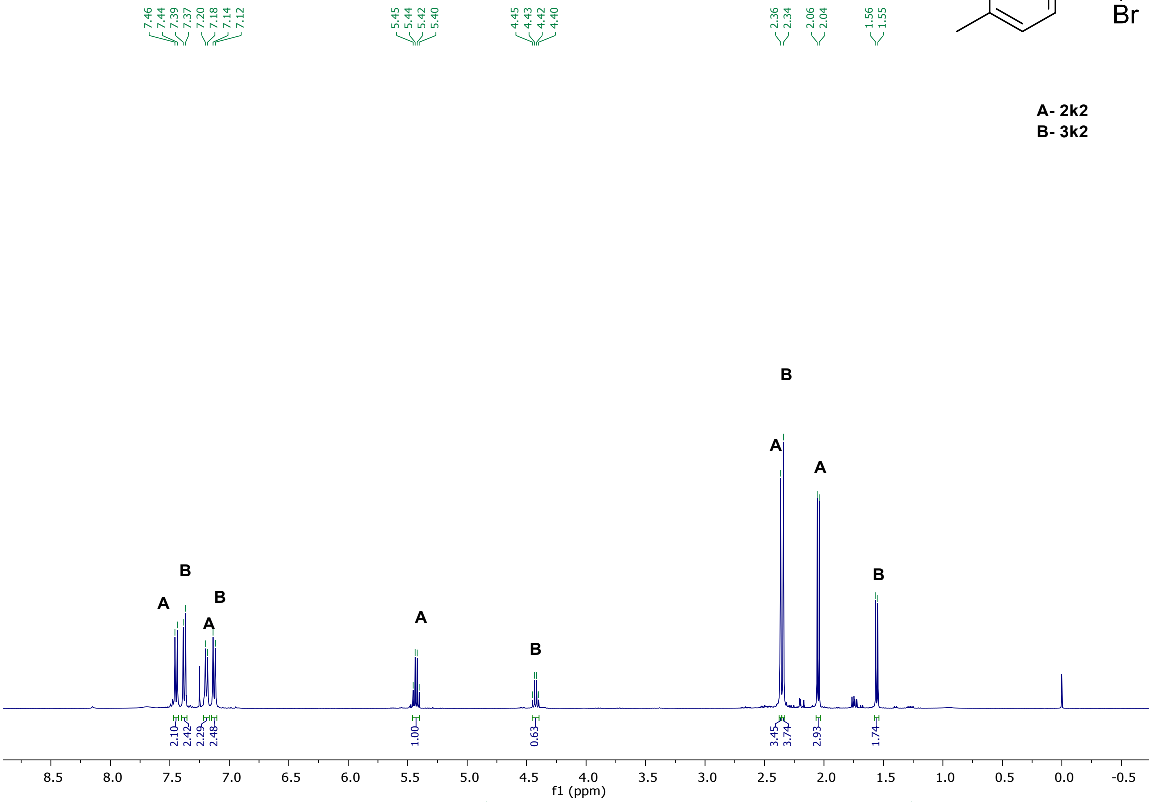

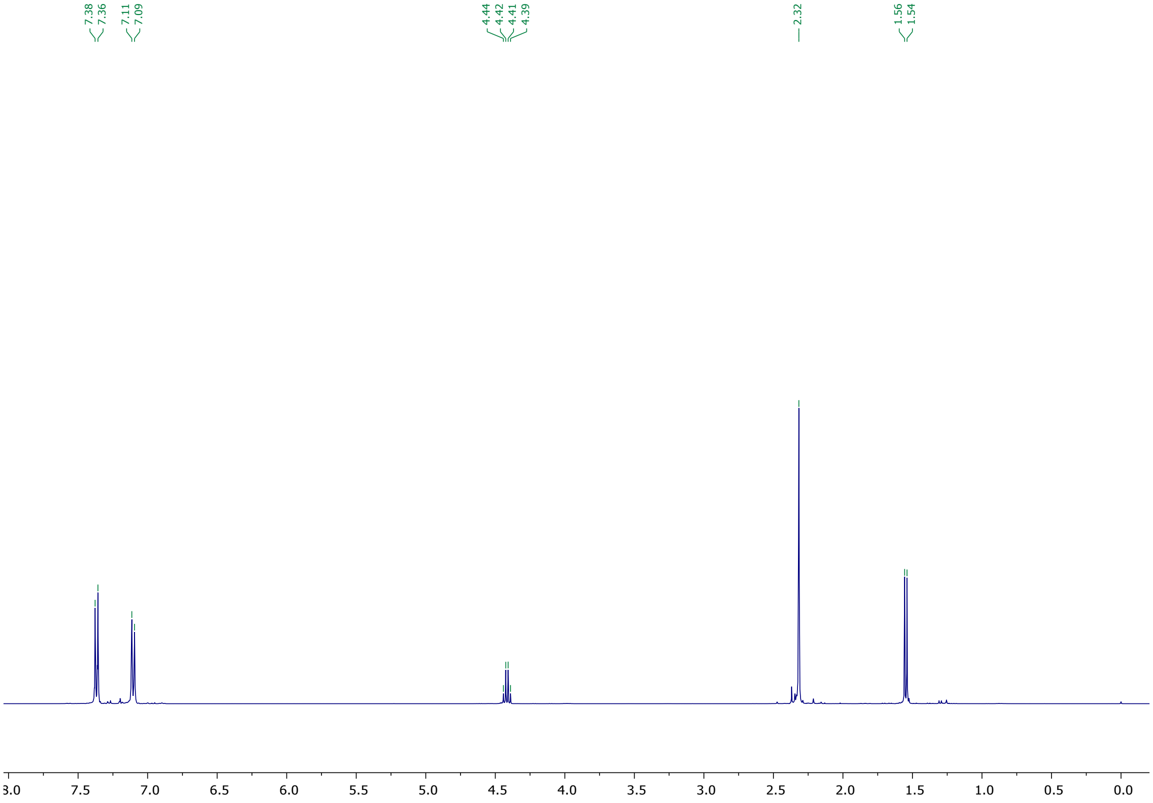

Ethane-1,1-diylbis(*p*-tolylsulfane) (3k2)

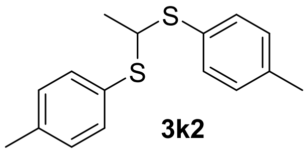

3k2

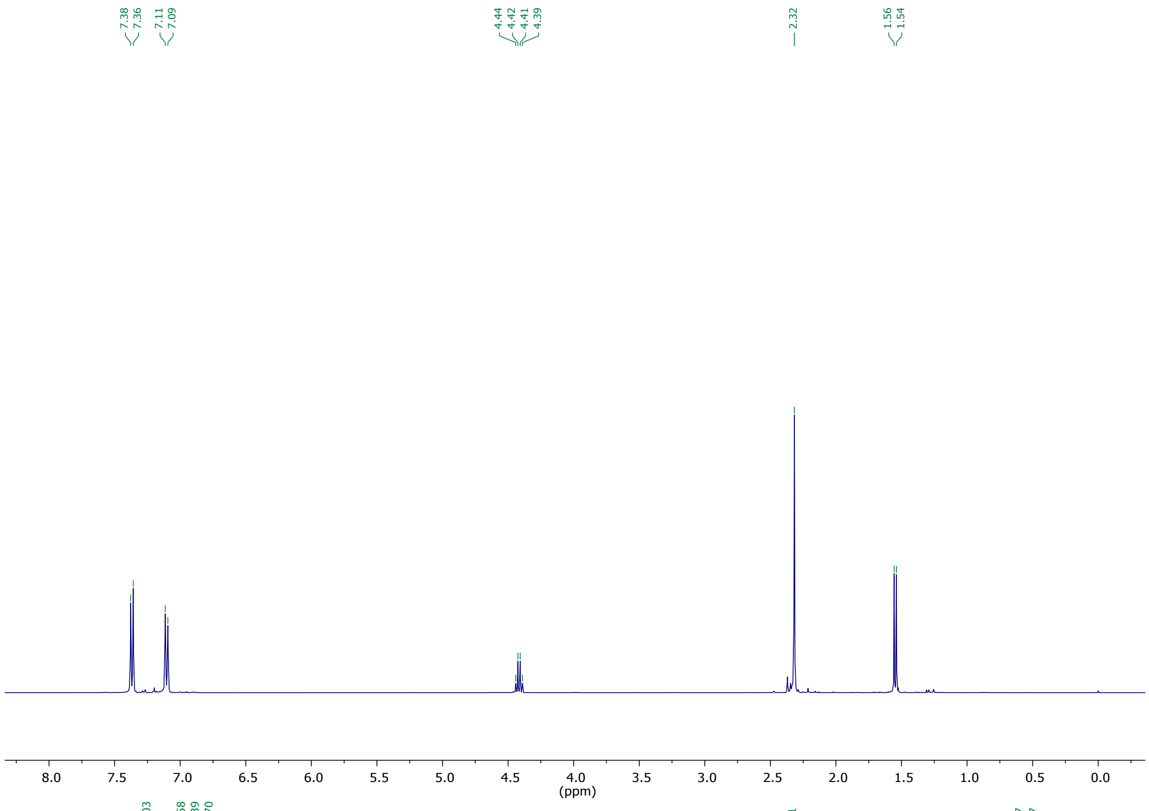
7.38
7.36
7.11
7.09
4.44
4.42
4.41
4.39
2.32
1.56
1.54
8.0 7.5 7.0 6.5 6.0 5.5 5.0 4.5 4.0 3.5 3.0 2.5 2.0 1.5 1.0 0.5 0.0
(ppm)

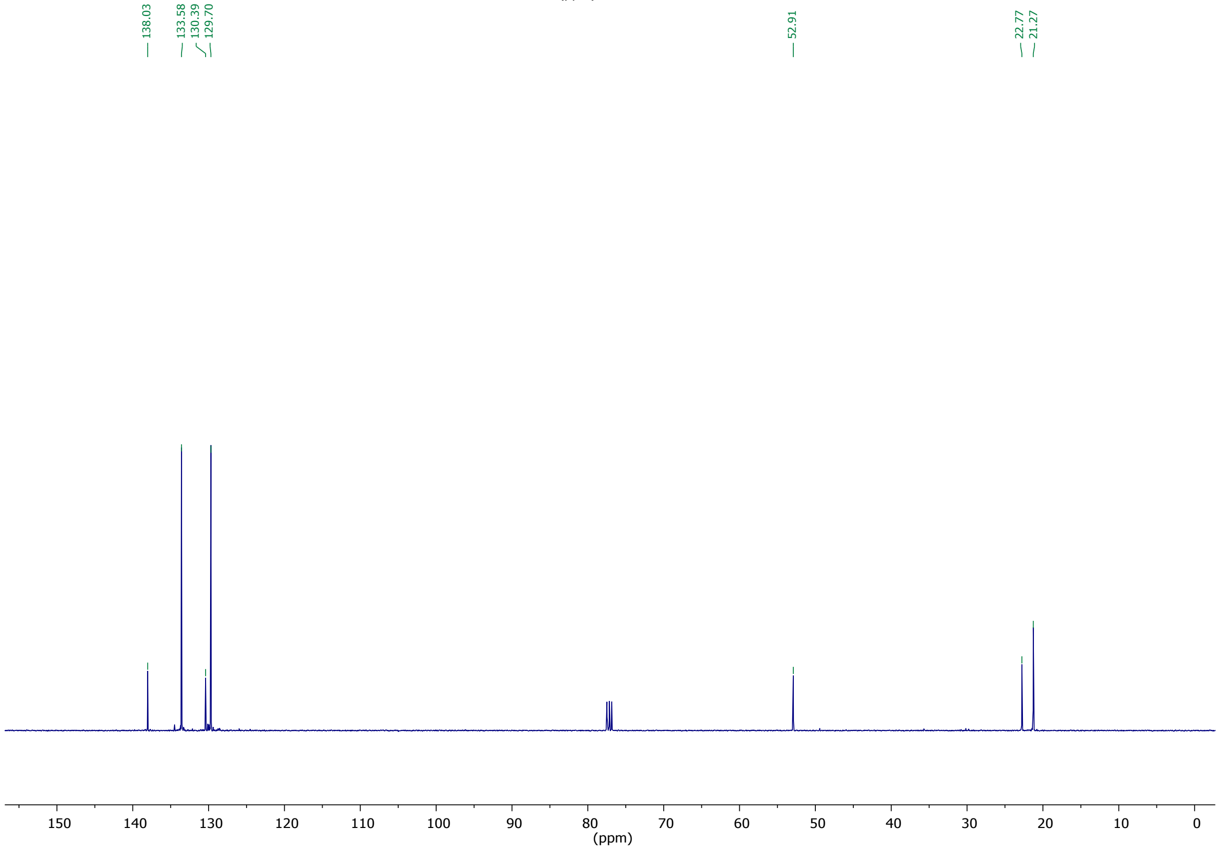
138.03
133.58
130.39
129.70
52.91
22.77
21.27
150 140 130 120 110 100 90 80 70 60 50 40 30 20 10 0
(ppm)

(Bromo(phenyl)methyl)(*p*-tolyl)sulfane (2k3)

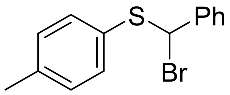

2k3

A- 2k3
B- 3k3
C -Benzaldehyde

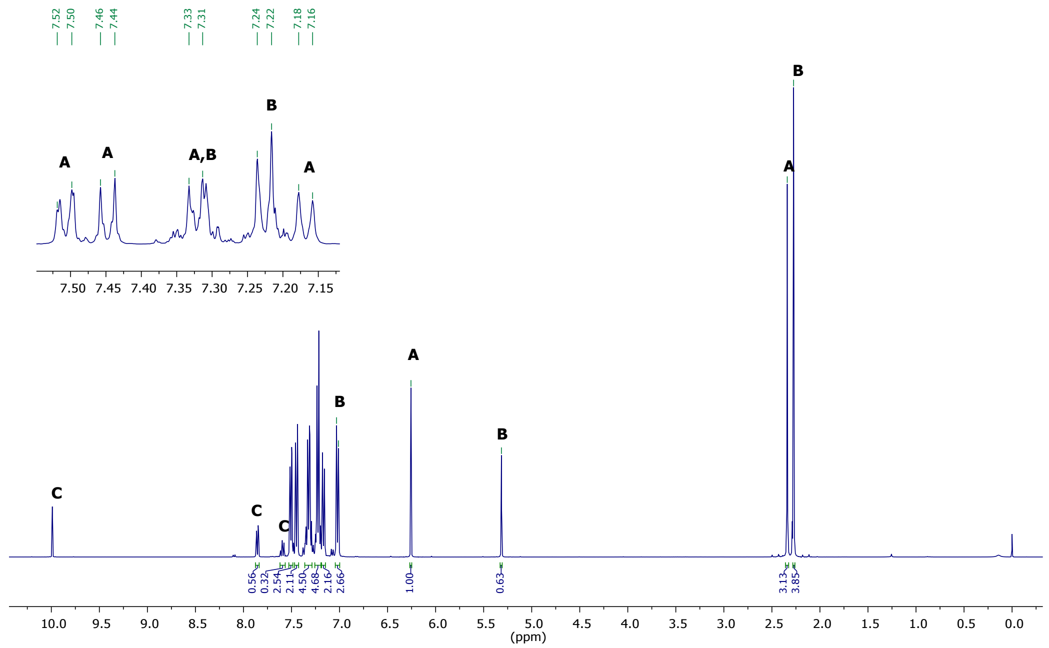

A- 2k3
B- 3k3
C -Benzaldehyde

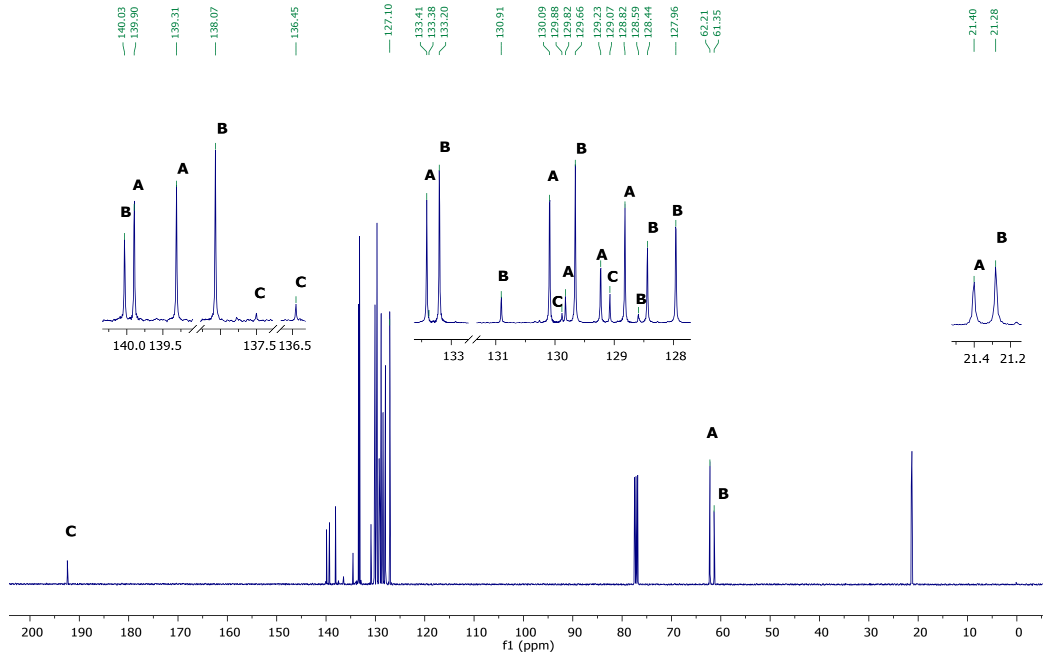

(Phenylmethylene)bis(*p*-tolylsulfane) (3k3)

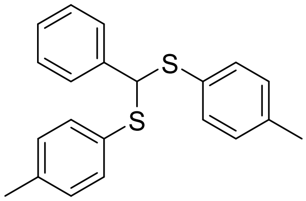

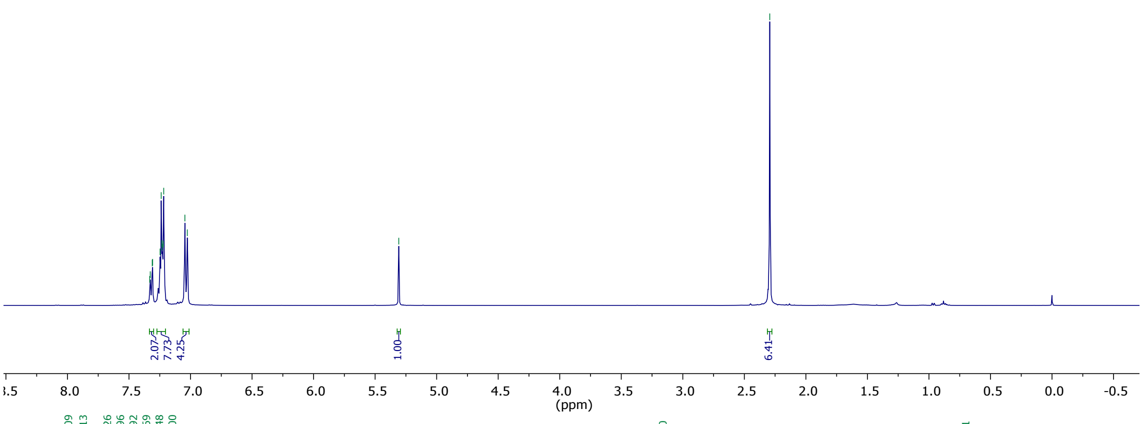

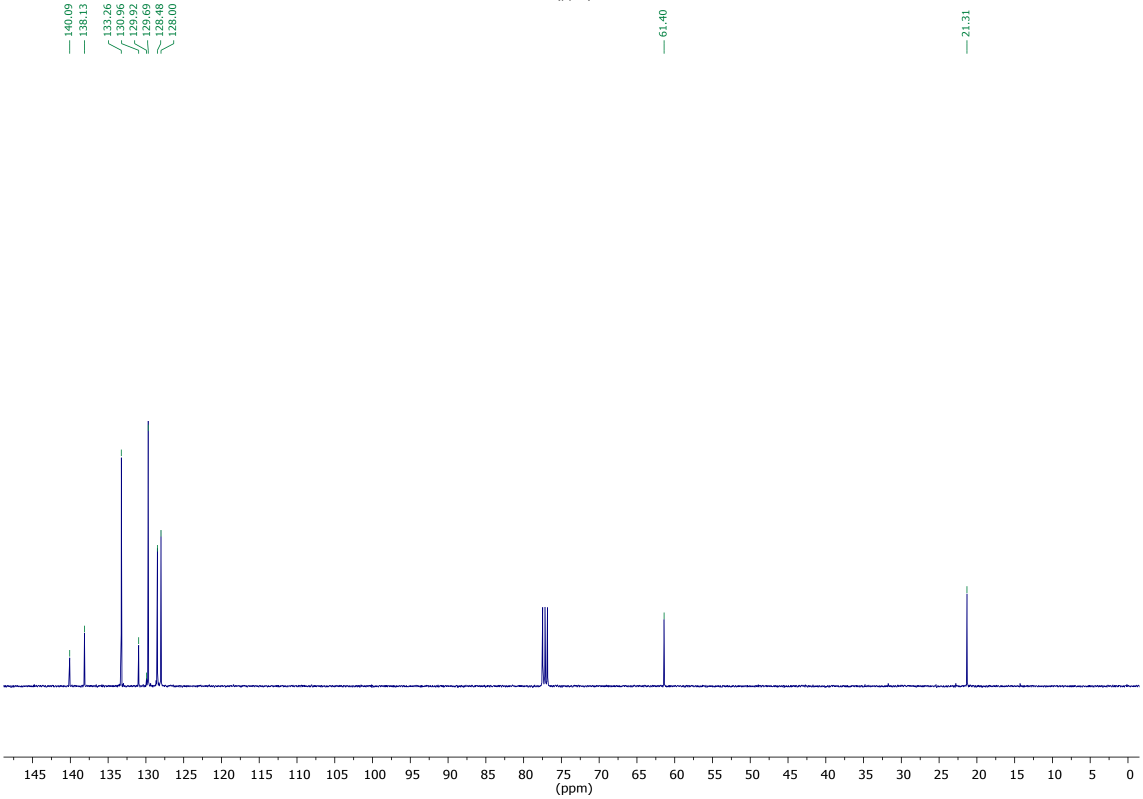

Benzyl(bromo(phenyl)methyl)sulfane (2a3)

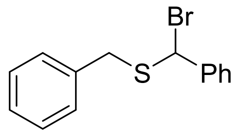

A- 2a3
B- 3a3
C -Benzaldehyde

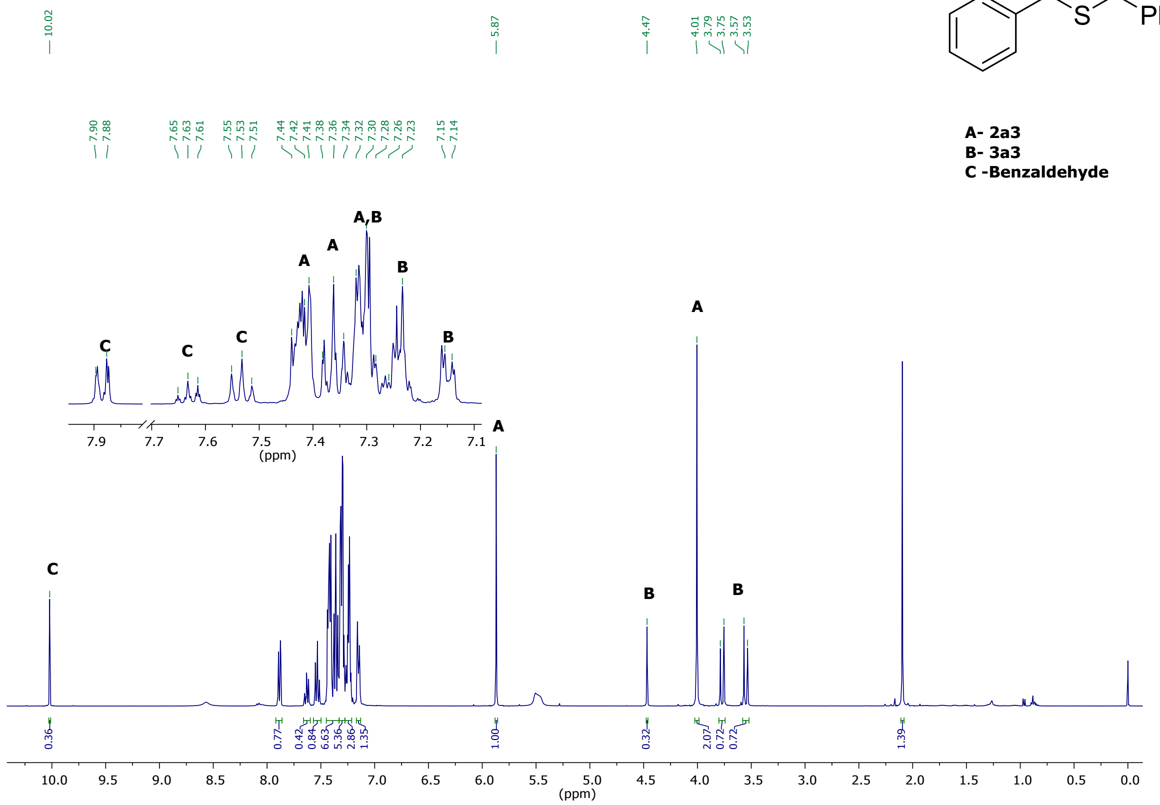

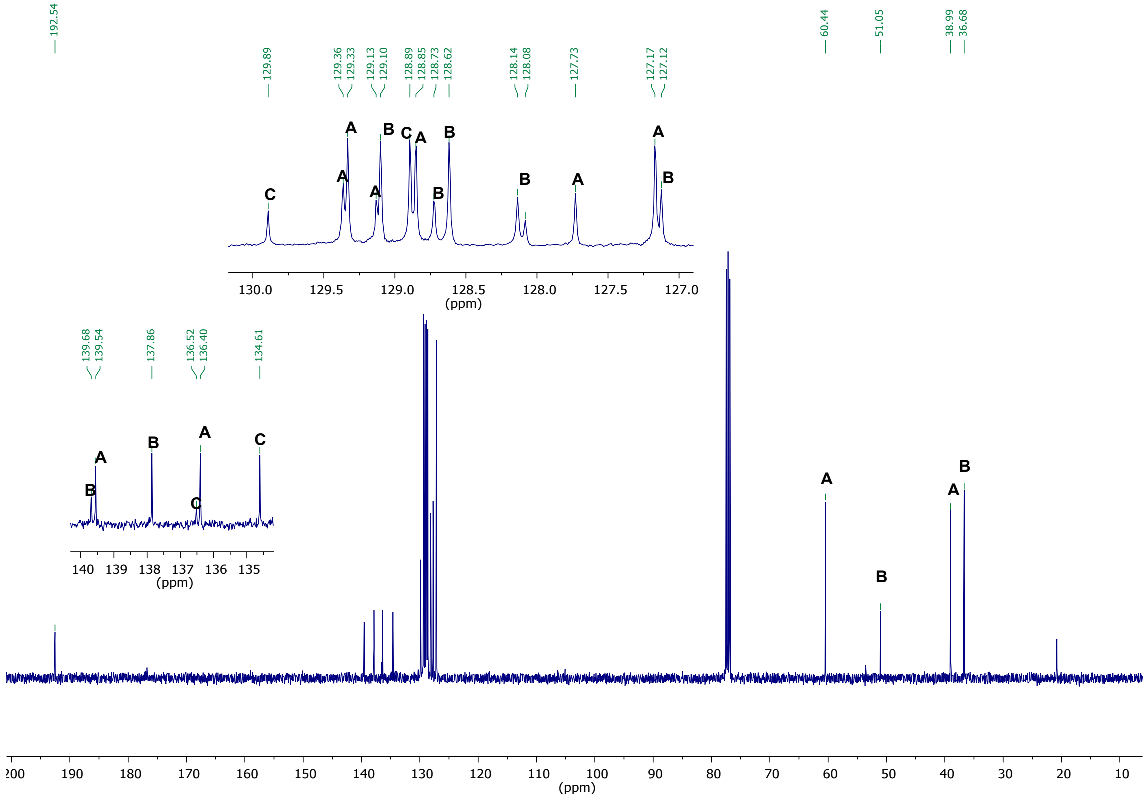

(Phenylmethylene)bis(benzylsulfane) (3a3)

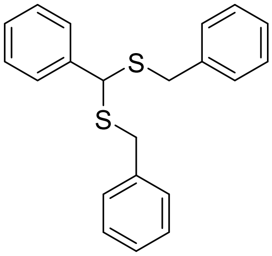

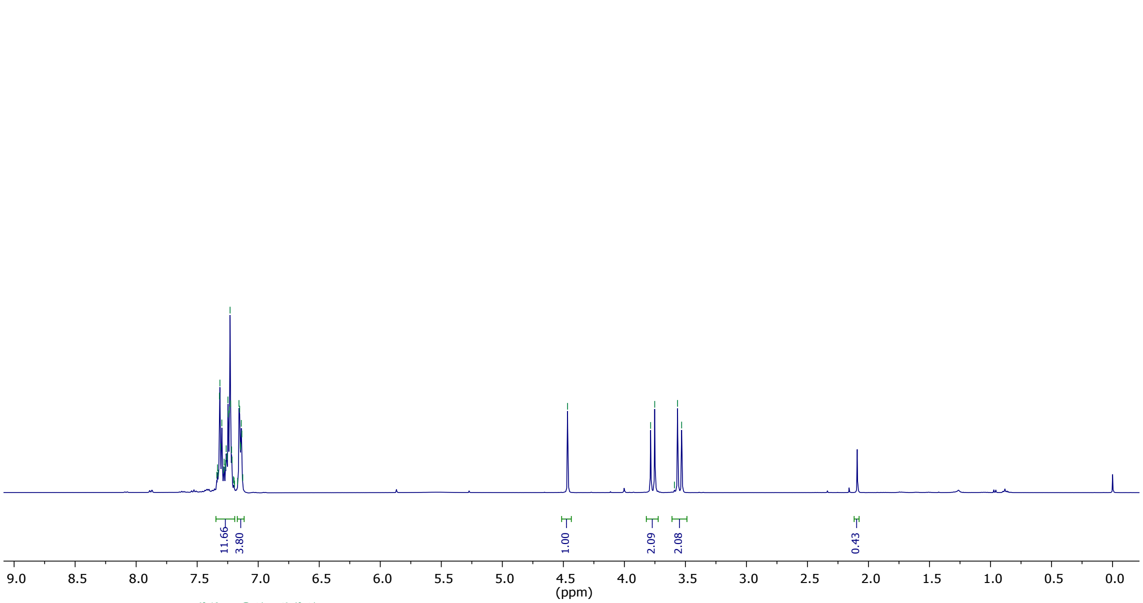

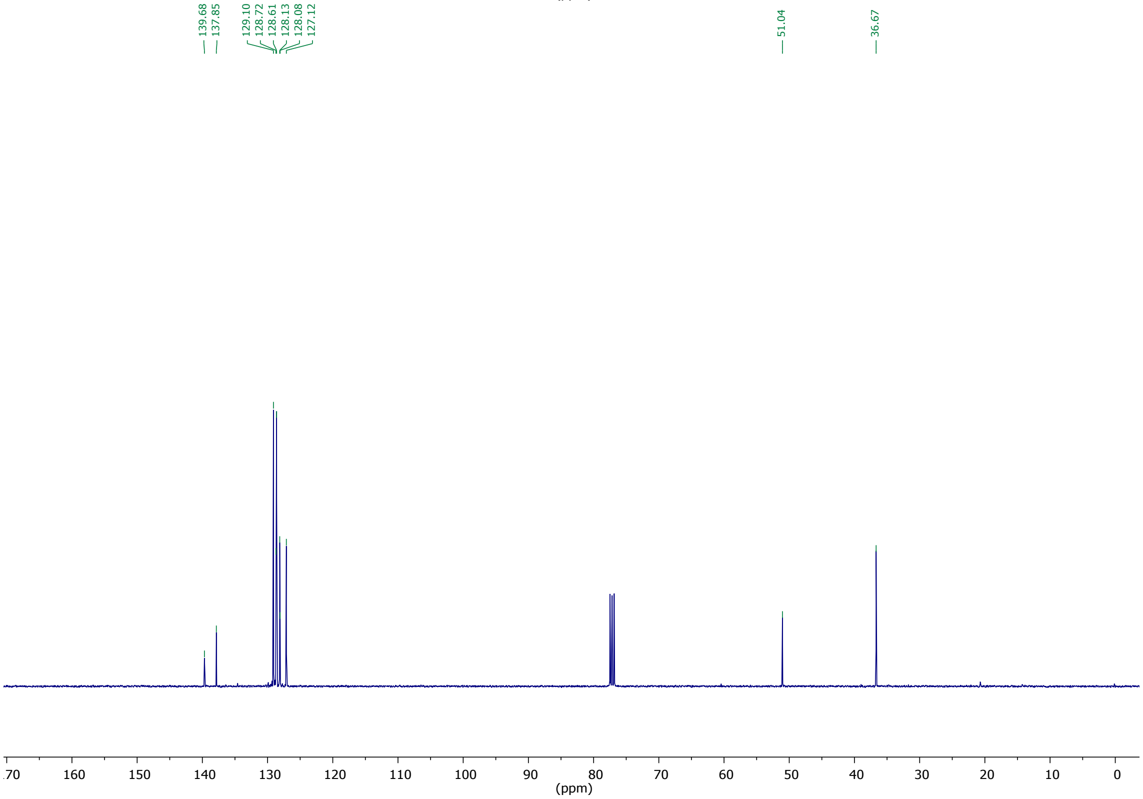

2-(Cyclohexylthio)-1-phenylethan-1-ol (5f)

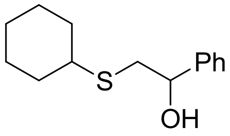

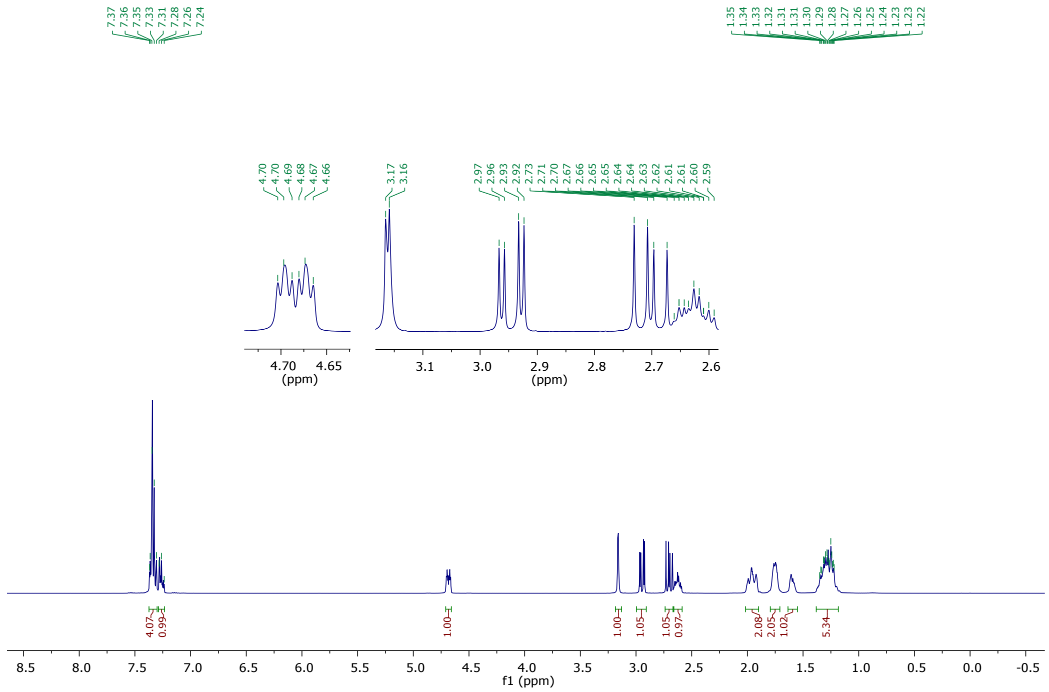

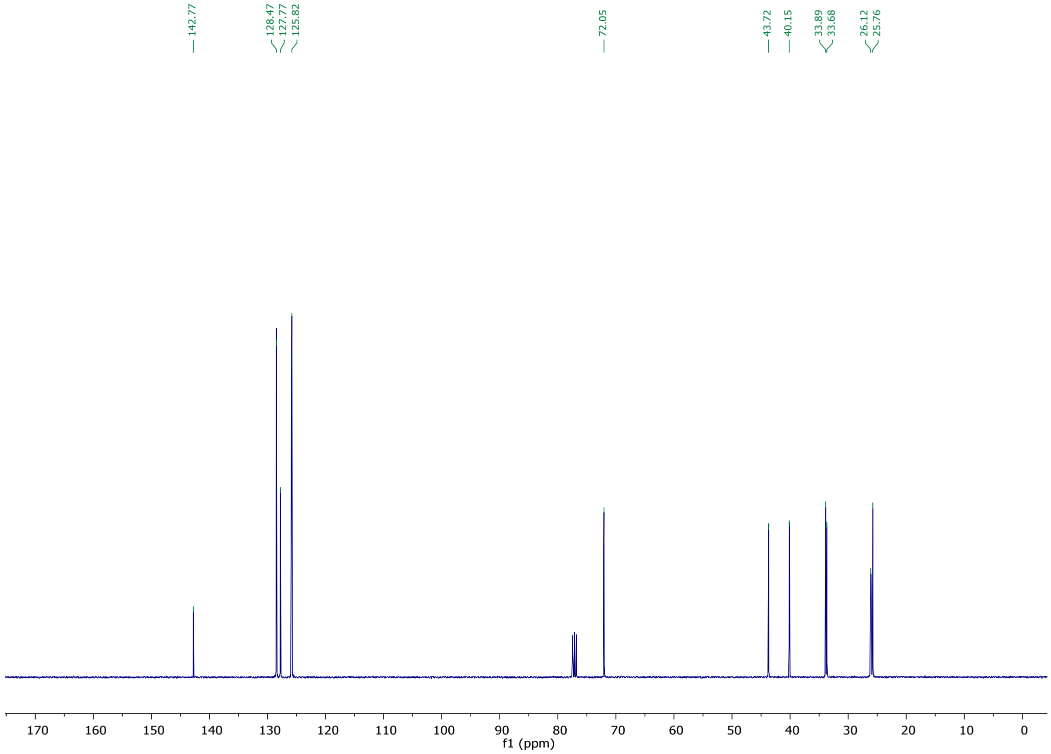

2-(((1*S*,2*S*,5*R*)-2-Isopropyl-5-methylcyclohexyl)thio)-1-phenylethan-1-ol (5h)

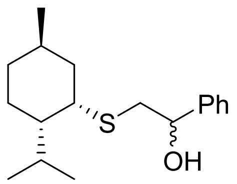

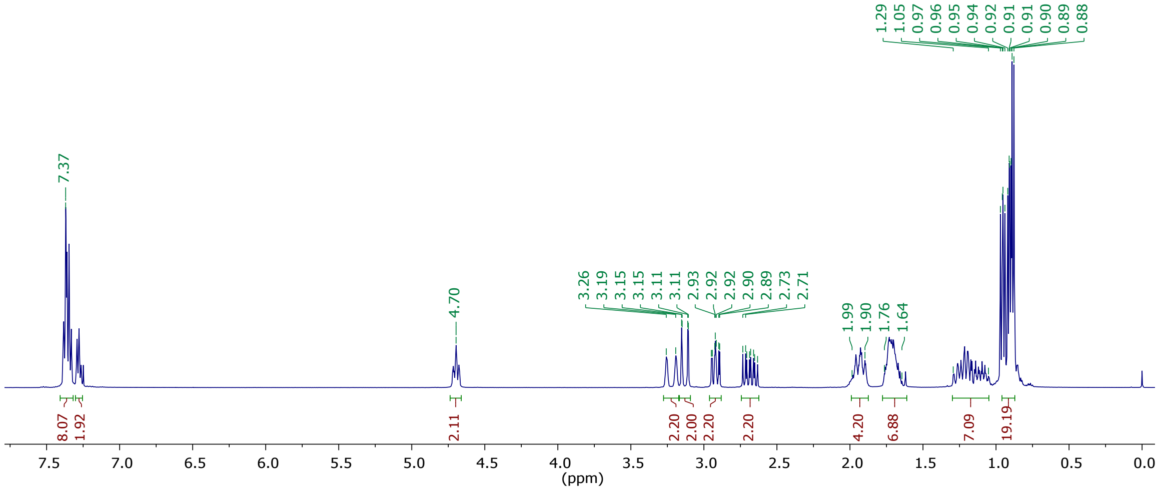

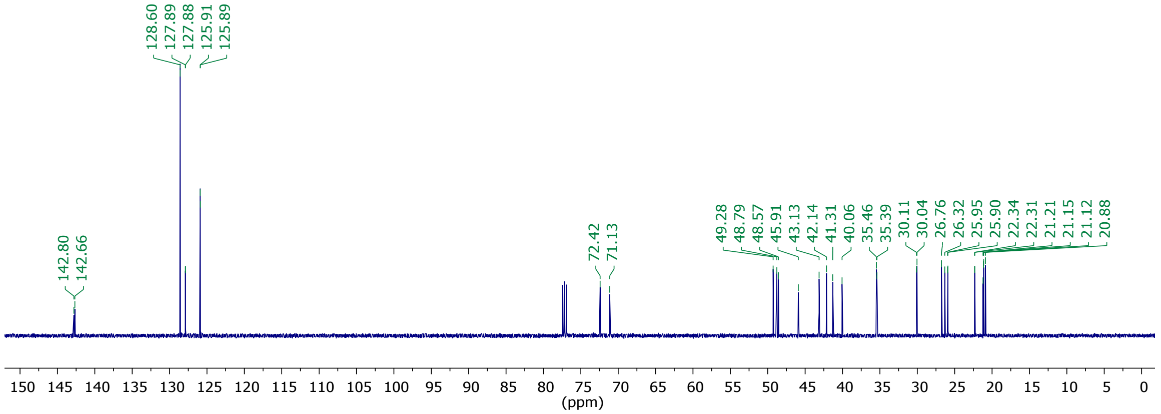

1-Phenyl-2-(*p*-tolylthio)ethan-1-ol (5k)

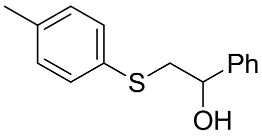

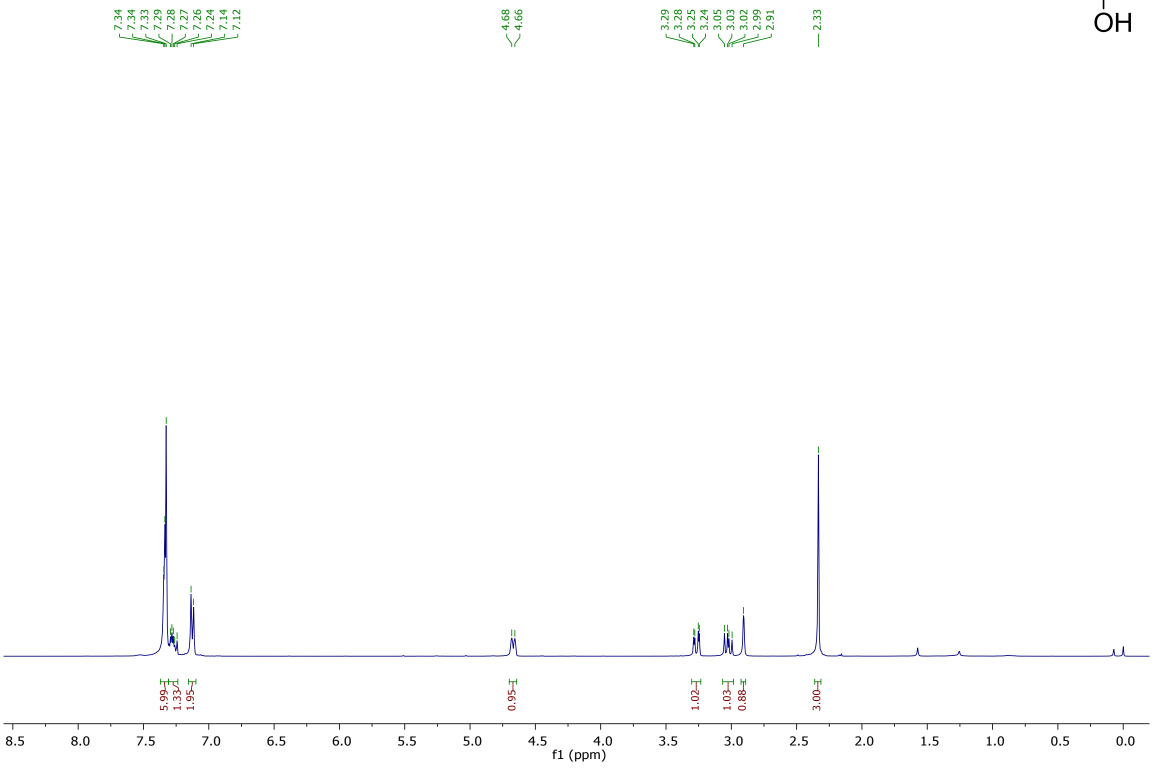

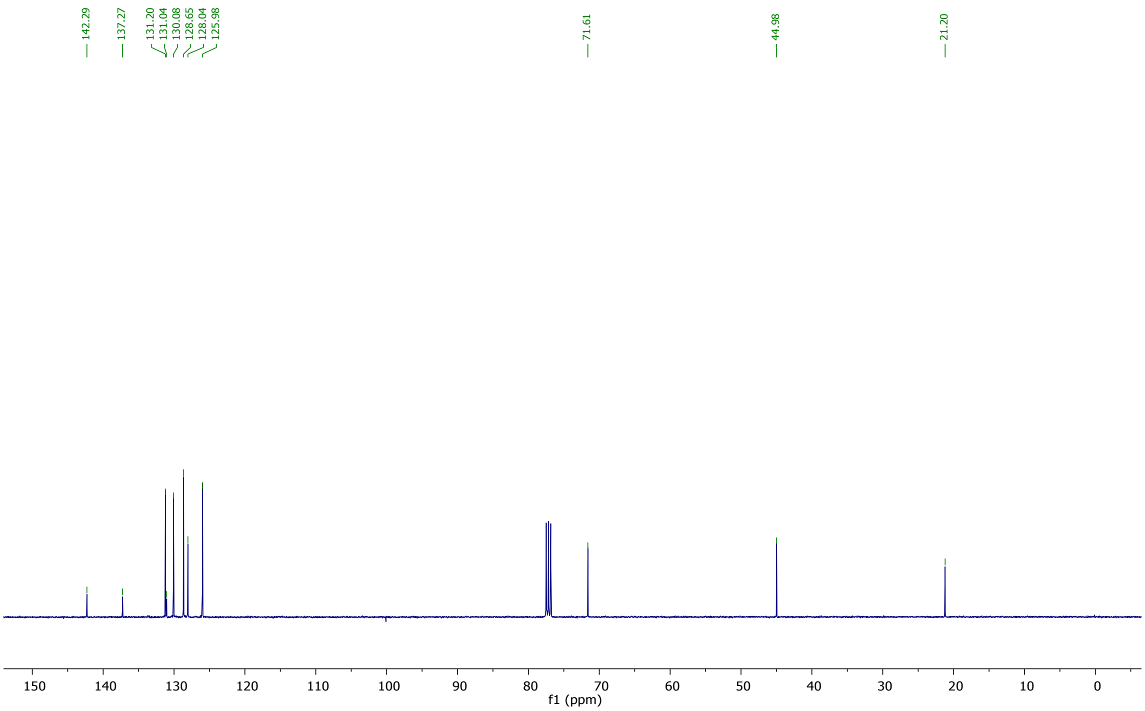

Cyclohexyl((phenylthio)methyl)sulfane (6f):

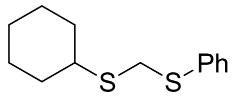

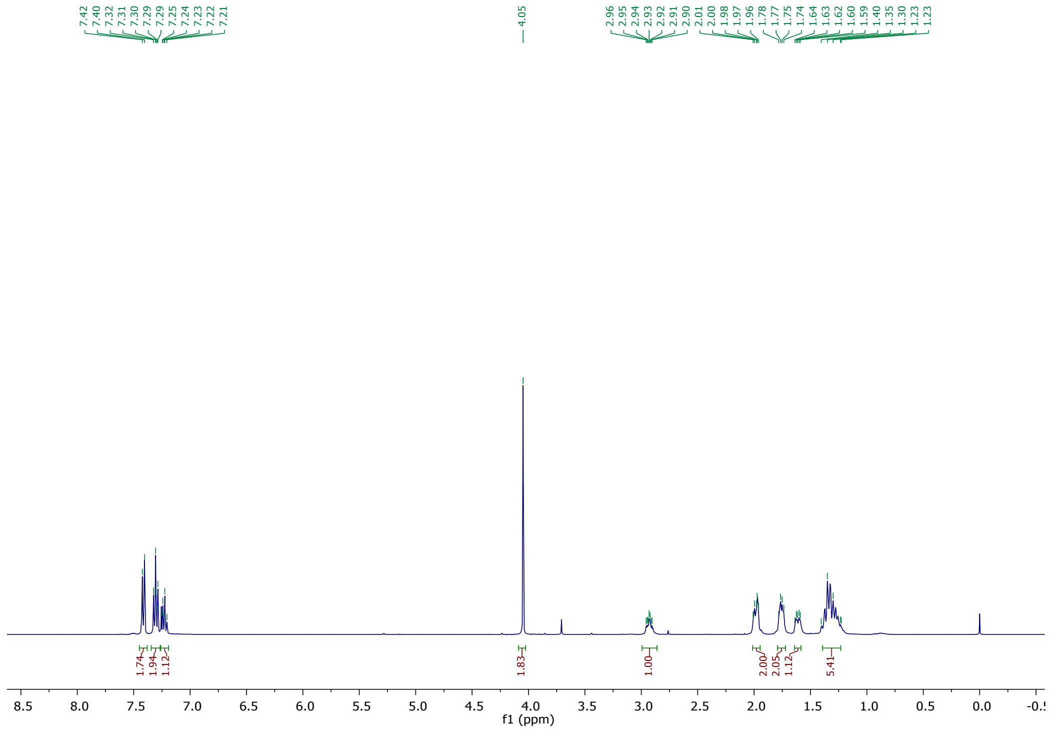

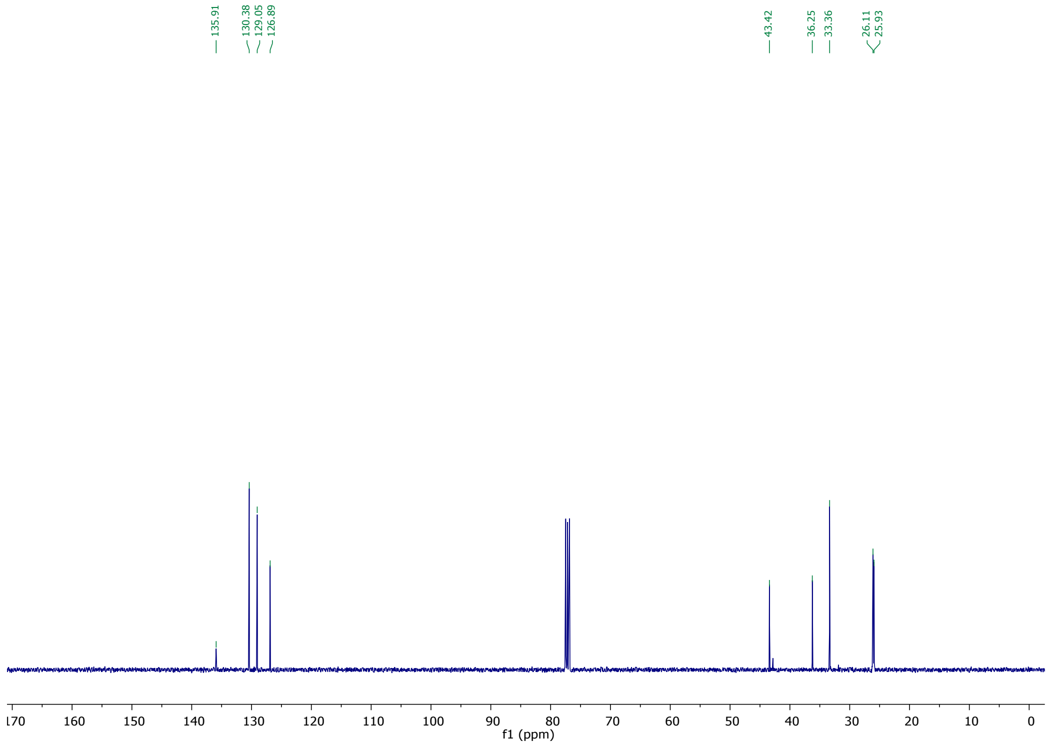

Phenyl((*p*-tolylthio)methyl)sulfane (6k)

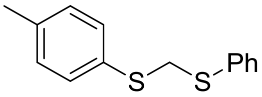

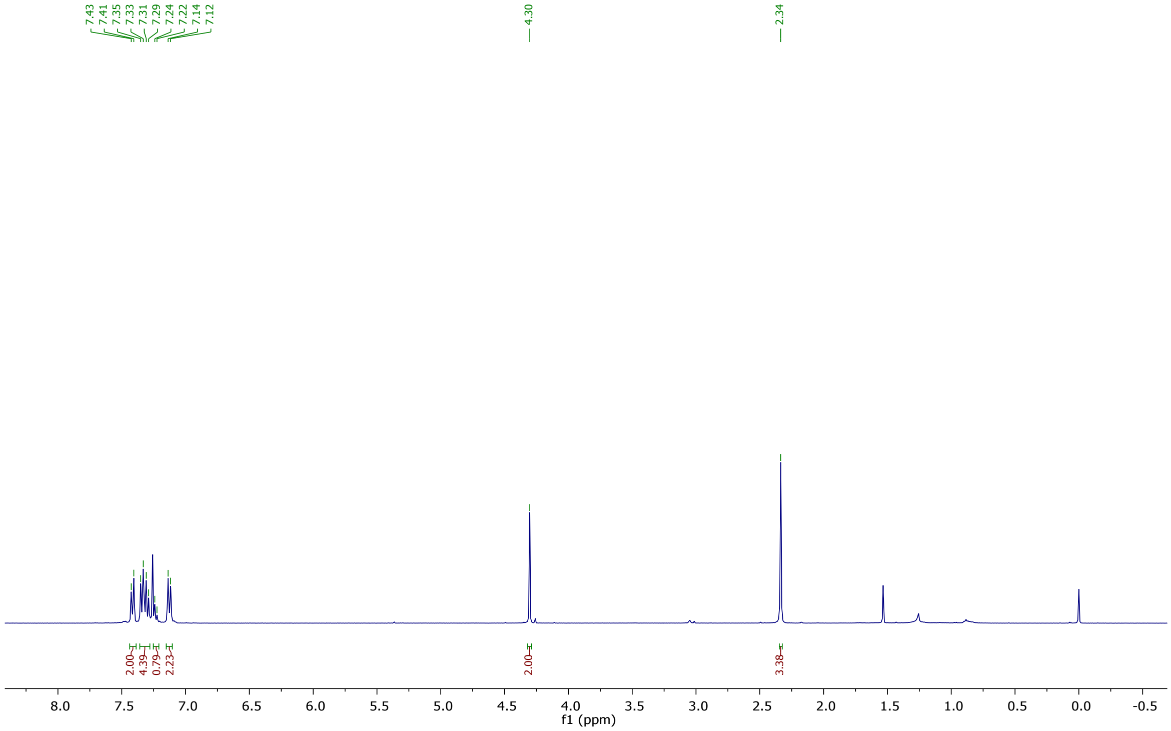

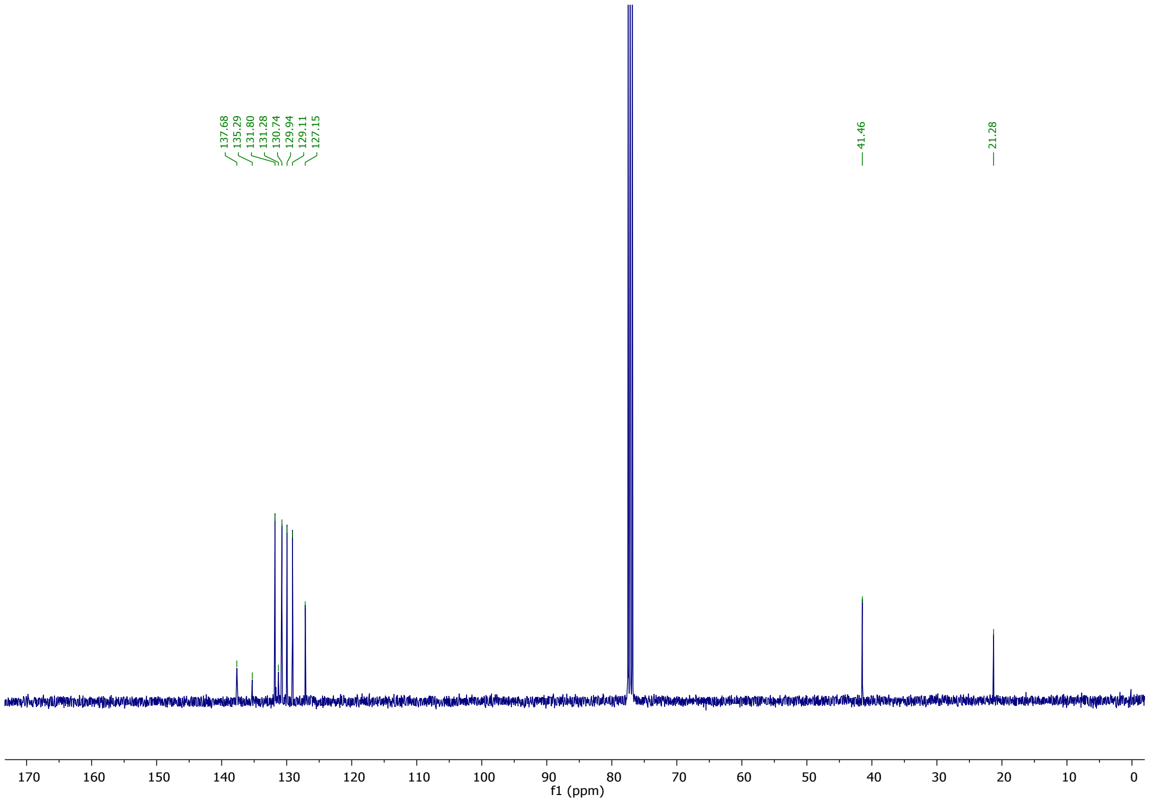

4-(Cyclohexylthio)butanenitrile (9f):

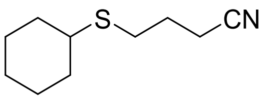

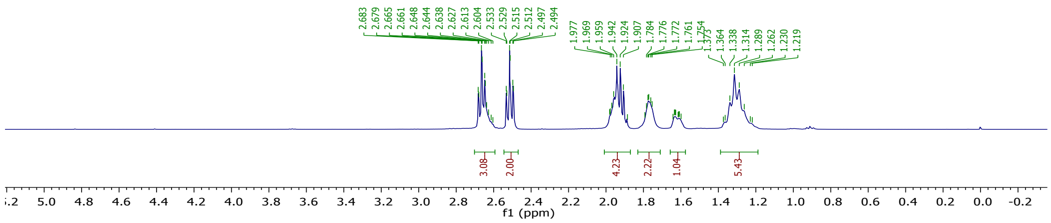

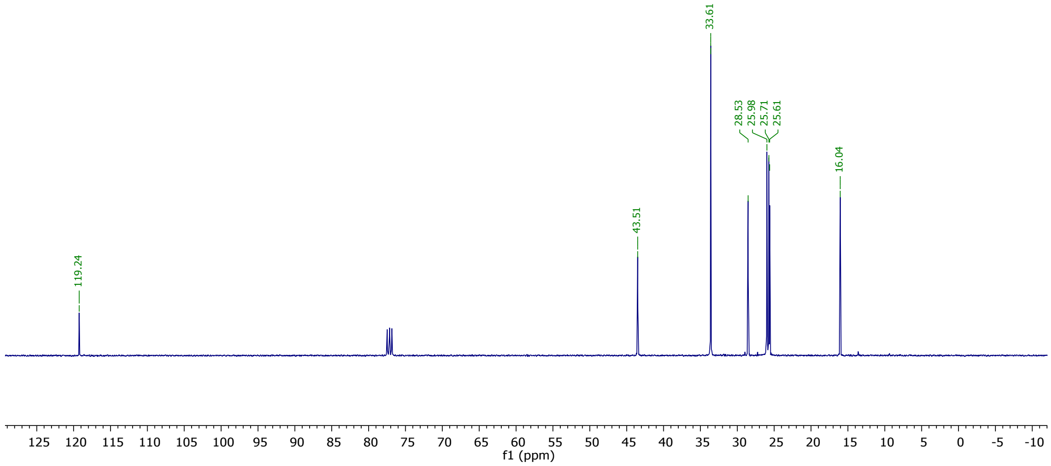

4-(*p*-Tolythio)butanenitrile (9k)

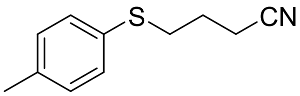

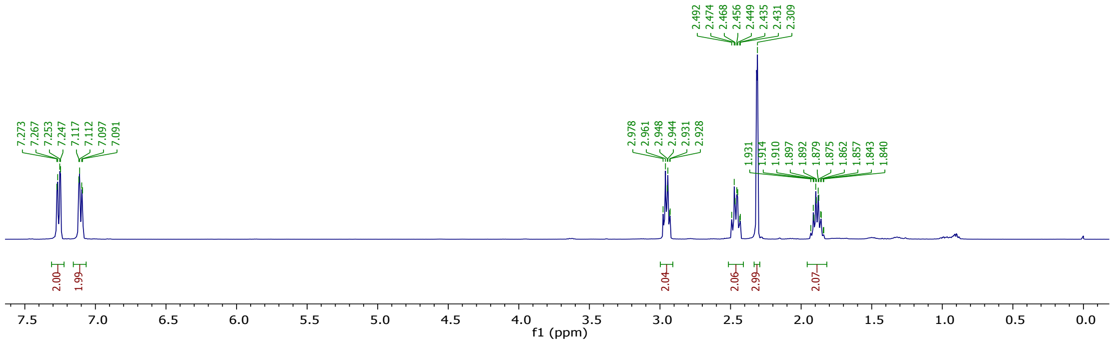

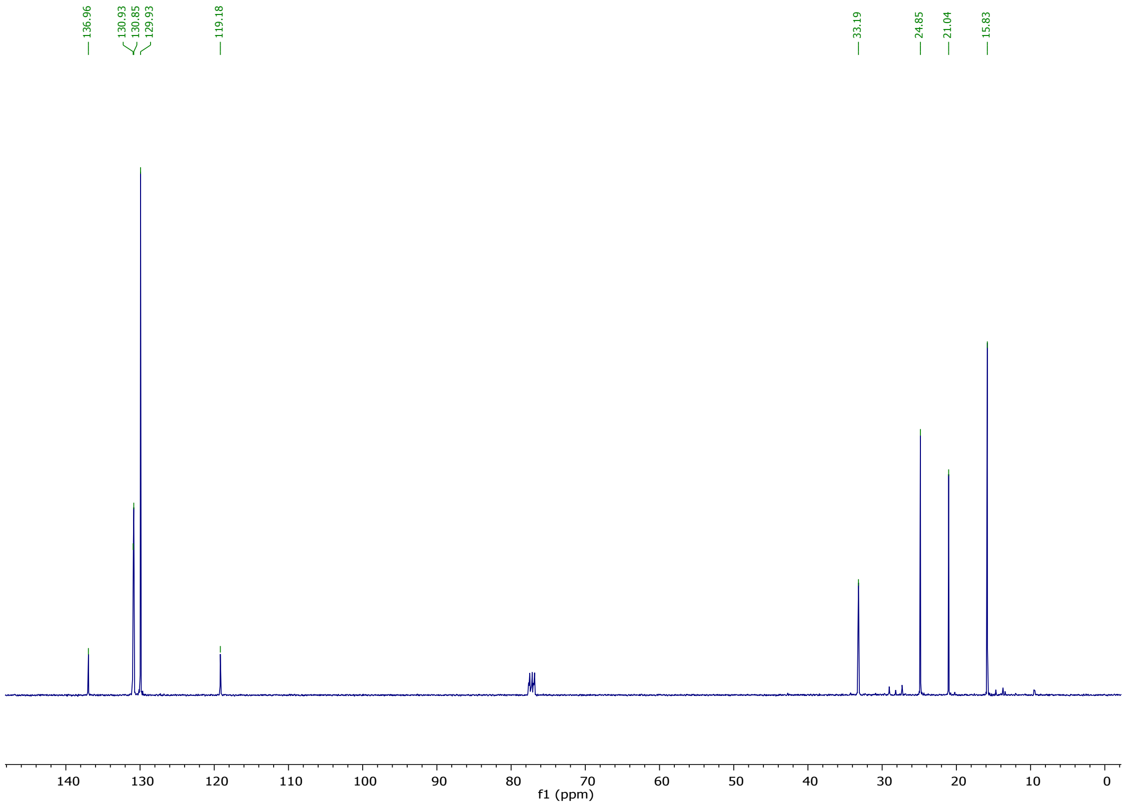

Methyl 4-(cyclohexylthio)butanoate (10f)

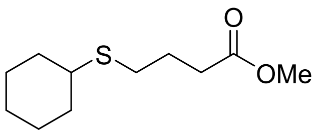

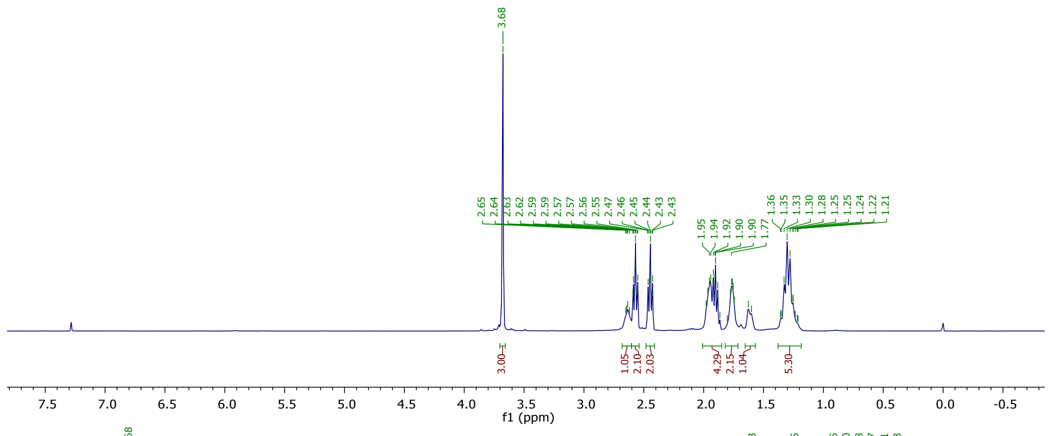

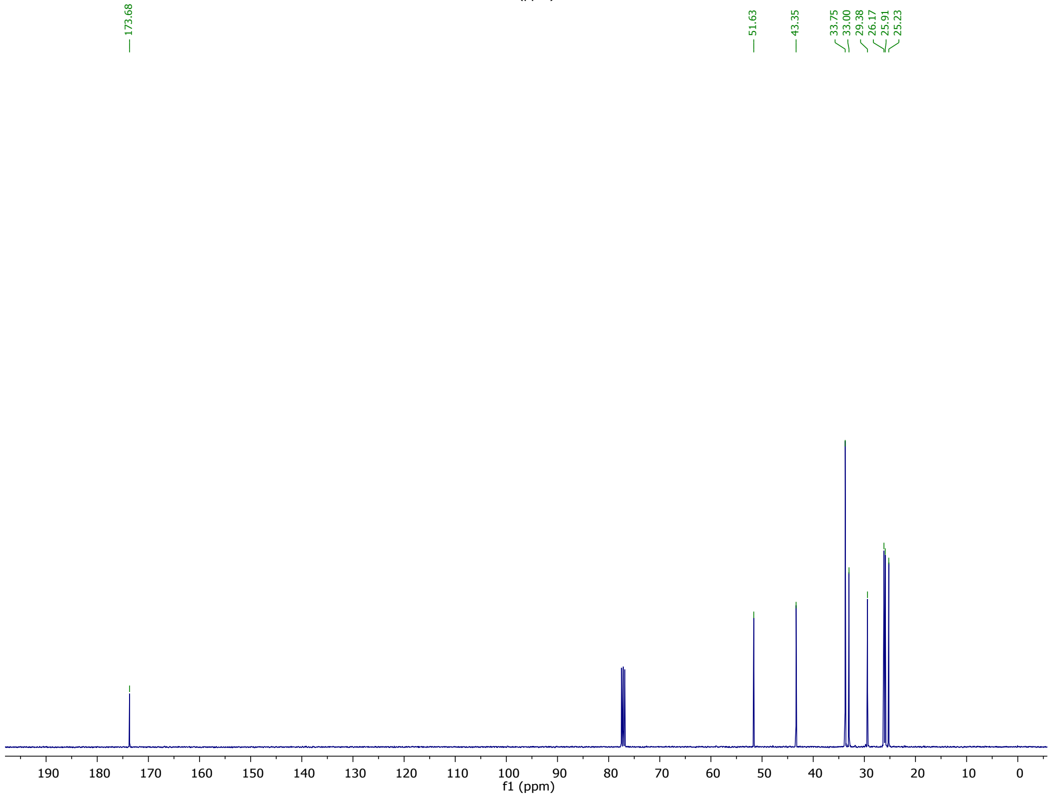

Methyl 4-(*p*-tolylthio)butanoate (10k)

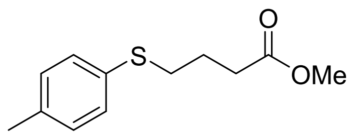

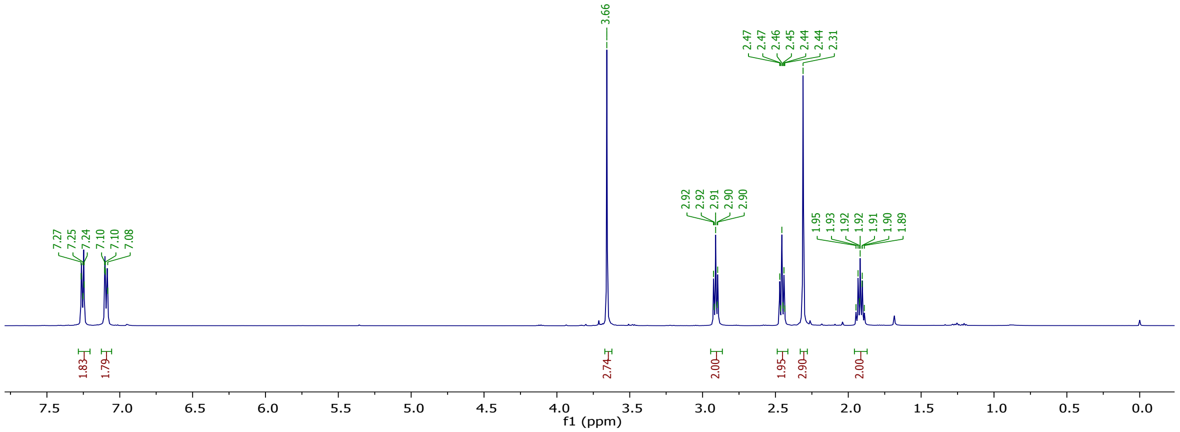

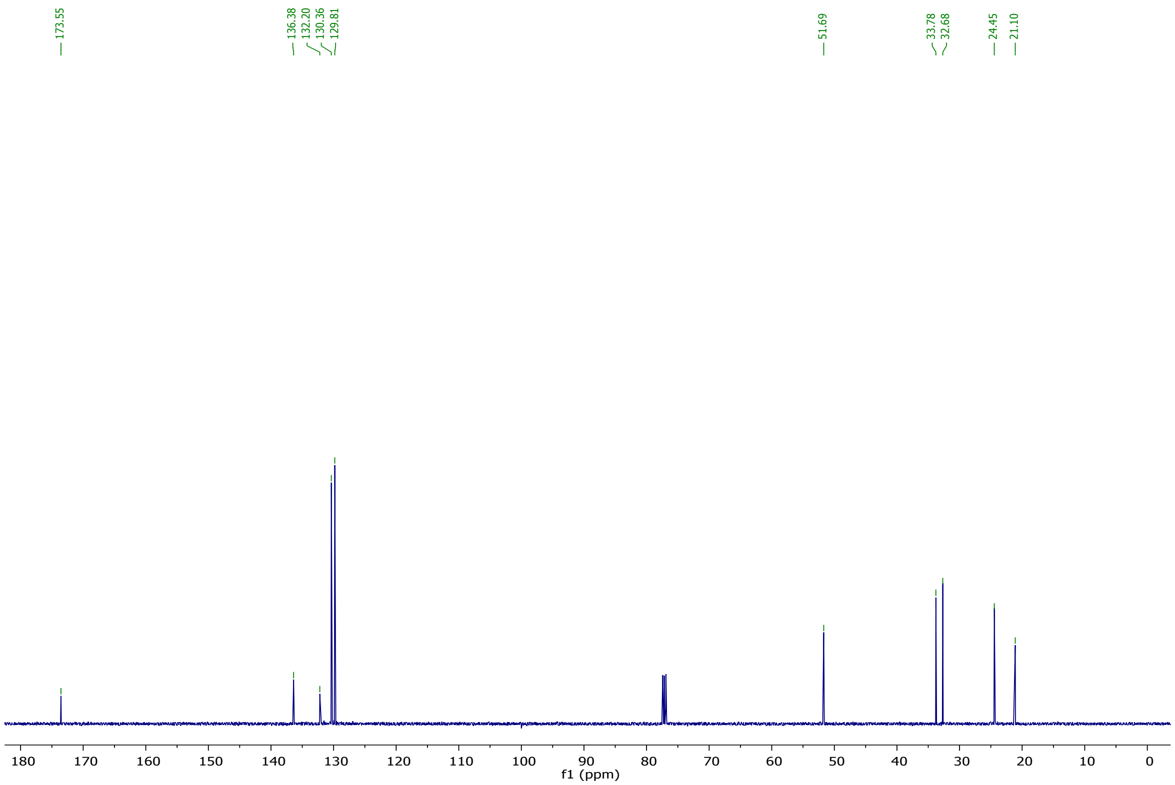

Supplement: RA-008-C8RA04002H-s001 [file RA-008-C8RA04002H-s001.pdf]
